# Imino‐Pyrrole Zn(II) Complexes for the Rapid and Selective Chemical Recycling of Commodity Polymers

**DOI:** 10.1002/anie.202502845

**Published:** 2025-04-02

**Authors:** Jack A. Stewart, Louis T. W. Powell, Matthew J. Cullen, Gabrielle Kociok‐Köhn, Matthew G. Davidson, Matthew D. Jones

**Affiliations:** ^1^ Department of Chemistry University of Bath Claverton Down Bath BA2 7AY UK; ^2^ Institute of Sustainability and Climate Change University of Bath Claverton Down Bath BA2 7AY UK

**Keywords:** Chemical recycling, Depolymerization, Homogeneous catalysis, Mixed plastic, Polymers

## Abstract

Three imino‐pyrrole zinc complexes were prepared and applied to the rapid degradation of polylactic acid (PLA) and the depolymerization of bisphenol A polycarbonate (BPA‐PC) and polyethylene terephthalate (PET). PLA alcoholysis proceeded rapidly at a range of conditions, including reflux in air. Remarkable activity was demonstrated for the solvent‐free methanolysis of PLA at mild conditions with full conversion reached in 11 min at 80 °C. Various conditions were investigated including a range of PLA sources and, importantly, catalyst recycling was demonstrated. The methanolysis of BPA‐PC and the glycolysis of PET were achieved, the latter giving full conversion after 1.5 h for all catalysts. The chemical recycling of mixed plastic feedstocks was investigated, including the selective and sequential degradation of a PLA/BPA‐PC mixture with a single catalyst and solvent.

## Graphical abstract

Three imino‐pyrrole zinc (II) catalysts were prepared and applied to the chemical recycling of PLA, PET, and BPA‐PC. Rapid PLA degradation was accomplished in solution and under solvent‐free conditions, the latter giving full conversion at relatively mild temperatures after 11 min. Mixed plastic feedstock experiments were carried out, including the first example of sequential PLA/BPA‐PC methanolysis using a single catalyst, with selectivity controlled by temperature.

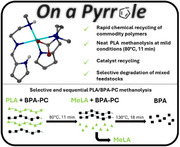

## Introduction

The widespread production of commodity plastics has had a profound influence on every aspect of contemporary life.^[^ ^1^ ^]^ In many cases, plastics have supplanted traditional materials like metals, glass, and wood as a result of cost‐effectiveness and the wide variety of properties that are available.^[^ ^2^ ^]^ However, issues around the reliance on fossil fuels and the catastrophic impact of plastic pollution have become increasingly stark.^[^ ^3^, ^4^, ^5^, ^6^, ^7^ ^]^ In the last 50 years, there has been a 20‐fold increase in global plastic production, reaching an annual output of 400 million tons, and it is estimated that plastic waste leakage into aquatic ecosystems will reach 509 million tons by 2060.^[^ ^8^ ^]^ Today, ≈8% of crude oil and natural gas extracted globally is used as a feedstock for the polymer industry.^[^ ^9^ ^]^ It is clear that these issues must be addressed to ensure a more sustainable future for the plastics industry.

A key component of a sustainable approach to plastics is chemical recycling, where polymers are broken down into small molecules that are value‐added (degradation or chemical upcycling) or can be directly repolymerized to give virgin material (depolymerization).^[^ ^10^, ^11^, ^12^, ^13^, ^14^, ^15^, ^16^ ^]^ This process supports a circular economy by preserving or enhancing the value of the original material through potentially infinite cycles.^[^ ^17^ ^]^ This shift has the potential to mitigate the environmental impact of plastic pollution and create economic incentives for industries to manage their post‐consumer waste responsibly.^[^ ^15^ ^]^ Legislative changes, such as Extended Producer Responsibility (EPR)^[^ ^18^ ^]^ in the UK and EU Directive 2019/904,^[^ ^19^ ^]^ are further promoting this transition.

Polyesters are particularly effective targets for chemical recycling as the ester groups are readily susceptible to nucleophilic attack.^[^ ^20^ ^]^ Polylactic acid (PLA) is a notable bio‐renewable polyester that offers several benefits, including renewable feedstocks, biocompatibility, and material properties that rival those of polyethylene terephthalate (PET) and polystyrene (PS) in some cases.^[^ ^21^, ^22^ ^]^ PLA is increasingly used in packaging and the biomedical industry.^[^ ^23^, ^24^, ^25^, ^26^ ^]^ It is typically synthesized through the ring‐opening polymerization (ROP) of L‐lactide, often catalyzed by Sn(Oct)_2_.^[^ ^27^ ^]^ Research into safer and more active initiators has encompassed metal complexes from different periodic table groups including Group I,^[^ ^28^, ^29^, ^30^ ^]^ Mg/Ca,^[^ ^31^, ^32^, ^33^, ^34^, ^35^ ^]^ Group IV,^[^ ^36^, ^37^, ^38^, ^39^, ^40^ ^]^ Fe,^[^ ^41^, ^42^, ^43^, ^44^, ^45^, ^46^, ^47^, ^48^, ^49^ ^]^ and Group XIII.^[^ ^50^, ^51^, ^52^, ^53^, ^54^, ^55^, ^56^, ^57^ ^]^ Zinc complexes are particularly active for lactide ROP and remain at the forefront of research in this area.^[^ ^42^, ^58^, ^59^, ^60^, ^61^, ^62^, ^63^, ^64^, ^65^, ^66^, ^67^, ^68^, ^69^ ^]^

Zinc complexes have also been shown to effectively catalyze the alcoholysis of polyesters, such as PLA and PET.^[^ ^63^, ^64^, ^65^, ^66^, ^68^, ^70^, ^71^, ^72^ ^]^ The latter is important as it demonstrates the wider applicability of this technology.^[^ ^13^ ^]^ Typically, PLA is converted into lactate esters which are used as solvents, synthetic precursors, or as additives in the food and cosmetic industries.^[^ ^73^ ^]^ The target of PET depolymerization is often bis(2‐hydroxyethyl) terephthalate (BHET) that can be repolymerized to give virgin polymer.^[^ ^74^ ^]^

The Jones group has reported imino‐ and amino‐phenolate zinc complexes with different coordination motifs including {ONN},^[^ ^64^, ^65^, ^71^ ^]^ {ONS},^[^ ^67^ ^]^ and {ONO}.^[^ ^68^ ^]^ A complex based on N‐methyl‐1,3‐propylenediamine with a bis(*t*‐butyl) substituted phenolate gave 81% yield of methyl lactate (MeLA) after 30 min at mild conditions and was also shown to depolymerize PET with benzyl alcohol.^[^ ^64^ ^]^ A half‐salan zinc complex with bromo substituents reported by Payne and co‐workers was shown to degrade PLA to MeLA in 1.5 h at 8 wt% catalyst loading at 50 °C.^[^ ^71^ ^]^ Interestingly, this catalyst could also promote aminolysis of PET to produce monomers suitable for polyester amide synthesis. A smaller analog based on ethylenediamine degraded PLA to MeLA with full conversion after 30 min at 50 °C with 8 wt% catalyst.^[^ ^72^ ^]^ Room temperature degradation was achieved by Lamberti et al. with a pyridyl zinc monophenolate complex which gave a 74% MeLA yield after 2 h.^[^ ^75^ ^]^ Herres‐Pawlis et al. published a {NO} guanidine complex which was capable of PLA and PET alcoholysis.^[^ ^61^ ^]^ They also explored the scope of PLA degradation by producing ethyl, n‐butyl, and t‐butyl lactates, all of which are used industrially. Similar work from the same group used a bisguanidine catalyst to depolymerize PLA, PET, and poly ε‐caprolactone (PCL) with a range of alcohols.^[^ ^76^ ^]^

The separation of plastic waste is a costly and time‐consuming process and is one of the major challenges for mechanical recycling.^[^ ^77^ ^]^ It is therefore crucial for chemical recycling research to focus on selective degradation of mixed plastic waste streams. This field was recently reviewed by Zhang et al. who emphasized the benefits of selective and sequential degradation for making chemical recycling more efficient and economical for industrial applications at large scale.^[^ ^78^ ^]^ Wang and co‐workers highlighted the distinction between sequential and “one pot” depolymerization.^[^ ^79^ ^]^ The former refers to a process wherein polymers are depolymerized sequentially with product removal steps added in between the change of conditions required to degrade the next component. The latter denotes the simultaneous depolymerization of all components with subsequent separation and purification steps required. Mixtures of bisphenol A polycarbonate with PET (BPA‐PC/PET), PLA with poly(butylene succinate) (PLA/PBS), and PLA with polybutylene adipate terephthalate (PLA/PBAT) were degraded sequentially using Zn(HMDS)_2_ with temperature being the key to selectivity in this case.^[^ ^79^ ^]^ Hydrogenolysis of PLA and PET was reported by Klankermeyer using a ruthenium catalyst with selectivity again controlled by reaction temperature.^[^ ^80^ ^]^ The Dove group published the first example of a three‐component sequential glycolysis of PLA, BPA‐PC, and PET using three commercially available catalysts that were used to control selectivity, along with temperature and solvent choice.^[^ ^81^ ^]^ To the best of our knowledge, this is the only reported example of selective and sequential alcoholysis of a PLA/BPA‐PC mixture, although two separate catalysts were required. A one‐pot example with PLA/BPA‐PC was published by Srivastava et al. using a functionalized ionic liquid.^[^ ^82^ ^]^ Individually, BPA‐PC and PLA were converted at 120 °C in 1 and 4 h, respectively. Six hours were required to degrade and depolymerize the mixture of polymers, followed by efficient separation and purification. A recent paper from our group demonstrated PET depolymerization in the presence of high‐density polyethylene (HDPE) and poly(vinyl chloride) (PVC), with minimal loss of activity, in accordance with previous literature.^[^ ^68^, ^83^ ^]^ Selective and sequential degradation of PLA and PET was also demonstrated. The first example of mixed feedstock alcoholysis using polyoxymethylene (POM) was recently reported including BPA‐PC/POM and POM/PET mixtures where selectivity was controlled by temperature and catalyst choice.^[^ ^84^ ^]^ Saito and co‐workers developed the first four‐component sequential polymer recycling system with a TBD:TFA catalyst and selectivity was governed by temperature.^[^ ^83^ ^]^

**Scheme 1:**
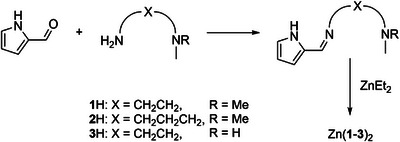
Synthesis of imino‐pyrrole ligands and complexes.

Herein, we present three zinc complexes bearing iminopyrrole ligands with the potential for {NN} or {NNN} coordination (Scheme 1). The complexes vary in the spacing between imine and pendant amine (ethyl or propyl) and amine substitution (NMe_2_ or NHMe). The complexes are characterized through ^1^H NMR, ^13^C{^1^H} NMR, elemental analysis (CHN), and single‐crystal x‐ray diffraction (XRD) where possible. The activity of these complexes toward *L‐*lactide polymerization and the degradation or depolymerization of commodity polymers is explored, including alcoholysis with and without a formal solvent and selective alcoholysis of mixed polymer feedstocks. The aim of this work is to develop robust catalysts for the selective degradation of commodity polymers.

## Results and Discussion

### Catalyst Synthesis and Characterization

Pyrrole carboxaldehyde was stirred with the relevant diamines for 24 h in methanol to afford the condensation products, **1**–**3**H, as dark red oils in good yields (93–98%). Ligands were characterized by ^1^H NMR spectroscopy, ^13^C{^1^H} NMR spectroscopy, and high‐resolution mass spectrometry (HRMS), all of which were consistent with the intended products (Figures S1–S6, Supporting Information).

Ligands **1**–**3**H were reacted with ZnEt_2_ in anhydrous toluene at a 2: 1 ratio with the goal of producing homoleptic ZnL_2_ complexes. Zn(**1**)_2_ and Zn(**2**)_2_ were isolated as dark red crystals suitable for single crystal XRD (Table S1, Supporting Information) and Zn(**3**)_2_ was collected as a pink solid. The ^1^H NMR spectra indicated the target homoleptic structure for each complex with one set of ligand resonances and no evidence of Zn‐Et peaks below 0 ppm (Figures S7–S12, Supporting Information). The spectrum of Zn(**3**)_2_ exhibited broad peaks which could be evidence of ligand fluxionality due to the lability of the pendant amine donor. Elemental analysis (EA) percentages aligned well with the intended values apart from Zn(**3**)_2_ where high levels of carbon and hydrogen could be attributed to traces of toluene in the sample which was visible by ^1^H NMR spectroscopy. Solid state structures were obtained for Zn(**1**)_2_, Zn(**2**)_2_, and Zn(**3**)_2_(CO_2_) and a full discussion of the crystallography and a ^1^H NMR study of air sensitivity can be found in the ESI (**Sections 2.3 and 2.4**).

### Lactide Polymerization

Complexes Zn(**1**–**3**)_2_ were tested as initiators for the polymerization of *L*‐lactide at industrially relevant melt conditions with benzyl alcohol used as a co‐initiator (Table S2, Supporting Information). An initial ratio of [LA]/[Zn]/[BnOH] = 300: 1: 1 was chosen, in line with previous literature on Schiff base zinc complexes.^[^ ^64^, ^67^, ^68^, ^70^, ^71^, ^72^, ^85^ ^]^ Zn(**1**)_2_ was the slowest catalyst at this ratio, taking 13 min to reach high conversion compared to 1 and 2 min, respectively, for Zn(**2**)_2_ and Zn(**3**)_2_. This mirrors the trend seen with analogous phenoxy‐imine initiators where propylenediamine‐based analogs were quicker than ethylenediamine counterparts.^[^ ^64^, ^85^ ^]^ Molecular weights were generally lower than expected, potentially due to serendipitous chain transfer agents in the monomer. At an increased ratio of [LA]/[Zn]/[BnOH] = 3000: 1: 10, Zn(**1**)_2_ was again the slowest initiator, taking 60 min to reach 17% conversion. Zn(**2**)_2_ was again the most active, reaching 71% conversion after 2 min with a TOF value of 63900 h^−1^. Reasonable activity was shown by Zn(**3**)_2_ giving 63% conversion after 6 min. The dispersity and molecular weight control improved significantly at this ratio, as has been previously observed when increasing the monomer to initiator ratio.^[^ ^67^, ^85^ ^]^ The most active initiators, Zn(**2**)_2_ and Zn(**3**)_2_, were subjected to further testing at a ratio of [LA]/[Zn]/[BnOH] = 10000: 1: 30, as there are numerous examples of zinc complexes that are effective at these concentrations.^[^ ^42^, ^64^, ^67^ ^]^ Unfortunately, no significant increase in viscosity was observed after 60 min at 130 °C. Zn(**2**)_2_ did achieve moderate conversion (44%) after this time, with a molecular weight of 28300 gmol^−1^, suggesting some deactivation of the complex leading to fewer chains initiated per metal center. SEC traces (Figures S22–S24, Supporting Information) show a shoulder toward high molecular weight. This is further evidence of inconsistent initiation and could result from multiple initiating species or the formation of cyclic PLA.

### PLA Methanolysis in Solution

Complexes Zn(**1**–**3**)_2_ were tested under an inert atmosphere for the methanolysis of commercial PLA at a range of conditions and analyzed using ^1^H NMR spectroscopy of the methine region where peaks for internal methine (Int), chain ends of small oligomers (CE) and MeLA are visible (Figure S25, Supporting Information). Reaction times were chosen for optimal kinetic data. The metrics *X*_int_, *S*_Me‐LA_, and *Y*_MeLA_ denote the PLA conversion, selectivity to MeLA, and yield of MeLA, respectively. The first tests were performed at 80 °C with 8 wt% catalyst loading in line with previous literature (Table 1).^[^ ^63^, ^67^, ^70^, ^71^ ^]^ In contrast to results obtained with analogous imino‐phenolate complexes,^[^ ^64^, ^65^ ^]^ the N,N‐dimethylethylenediamine‐based complex, Zn(**1**)_2_, outperformed the propylene‐bridged complex, Zn(**2**)_2_, in terms of PLA conversion and MeLA yield (Zn(**1**)_2_: *X*_int _= 96%, *Y*_MeLA_ = 71%; Zn(**2**)_2_: *X*_int _= 66%, *Y*_MeLA_ = 18%). Zn(**3**)_2_, however, was the most active catalyst by far, suggesting an active role for the pendant secondary amine group, potentially involving hydrogen bonding between the nitrogen proton and incoming polymer, as hypothesized with zinc half‐salan analogs.^[^ ^71^ ^]^ Activity was somewhat decreased for Zn(**3**)_2_ when the catalyst loading was lowered to 4 wt% giving 100% conversion and 83% yield after 1 h. A similar drop in activity was observed for the other two catalysts.

**Table 1:** Degradation of PLLA cup to methyl lactate via methanolysis using Zn(1–3)_2_ at 80 °C.^a)^

Cat.	Time [min]	Loading [wt%]	*Y*_MeLA_ [%]^b)^	*S*_MeLA_ [%]^b)^	*X*_int_ [%]^b)^	*k*_app_ [min^−1^]
Zn(**1**)_2_	30	8	71	74	96	0.064
Zn(**2**)_2_	30	8	18	27	66	0.029
Zn(**3**)_2_	30	8	98	98	100	0.365
Zn(**1**)_2_	60	4	43	51	84	0.020
Zn(**2**)_2_	60	4	15	25	61	0.011
Zn(**3**)_2_	60	4	83	83	100	0.210

^a)^ Reaction conditions: 0.25 g of PLLA cup (*M*_n_ = 45510 g mol^−1^), *V*_THF_:*V*_MeOH_ = 4:1, *n*_MeOH_:*n*_ester_ = 7:1, 4 – 8 wt% cat. loading (10–20 mg, 0.68–1.6 mol% relative to ester linkages).

^b)^ ^1^H NMR (400 MHz, 298 K, CDCl_3_) spectroscopy used to calculate *Y*_MeLA_, *S*_MeLA_, and *X*_Int_.

The reaction temperature was subsequently lowered to 50 °C to assess the activity of Zn(**1**–**3**)_2_ at milder conditions (Table 2). At 8 wt% catalyst loading, reactions were run for 30 min, and a sharp decline in activity was noted for Zn(**1**)_2_ compared with the 80 °C reaction. At the same time point, MeLA yield fell from 71% to 18%. Conversely, the conversion and yield achieved by Zn(**2**)_2_ increased from 66% and 18% to 73% and 24%, respectively, as the temperature was lowered. This is unusual and could be explained by a deactivation process at higher temperatures. Zn(**3**)_2_ still reached full conversion under these conditions with a MeLA yield of 76%. The reactions at 50 °C and 4 wt% showed a similar trend. A significant decrease in activity was noted for Zn(**1**)_2_ whereas Zn(**2**)_2_ and Zn(**3**)_2_ were relatively unchanged from the equivalent 80 °C reactions. Zn(**3**)_2_ attained full conversion and a 79% MeLA yield after 60 min, competitive with the most active phenoxy‐imine catalysts at the same conditions.^[^ ^64^, ^65^, ^71^ ^]^ Zn(**3**)_2_ was also shown to be somewhat active at 50 °C with a catalyst loading of 2 wt%, further demonstrating the efficacy of this catalyst under mild conditions.

**Table 2:** Degradation of PLLA cup to methyl lactate via methanolysis using Zn(1–3)_2_ at 50 °C.^a)^

Cat.	Time [min]	Loading [wt%]	*Y*_MeLA_ [%]^b)^	*S*_MeLA_ [%]^b)^	*X*_int_ [%]^b)^	*k*_app_ [min^−1^]
Zn(**1**)_2_	30	8	18	31	59	0.023
Zn(**2**)_2_	30	8	24	33	73	0.031
Zn(**3**)_2_	30	8	76	76	100	0.098
Zn(**1**)_2_	60	4	22	32	68	0.015
Zn(**2**)_2_	60	4	20	30	66	0.008
Zn(**3**)_2_	60	4	79	79	100	0.056
Zn(**3**)_2_	180	2	42	48	87	0.010

^a)^ Reaction conditions: 0.25 g of PLLA cup (*M*_n_ = 45510 g mol^−1^), *V*_THF_:*V*_MeOH_ = 4:1, *n*_MeOH_:*n*_ester_ = 7:1, 4–8 wt% cat. loading (10–20 mg, 0.68–1.6 mol% relative to ester linkages).

^b)^ ^1^H NMR (400 MHz, 298 K, CDCl_3_) spectroscopy used to calculate *Y*_MeLA_, *S*_MeLA_, and *X*_Int_.

Using the initial conditions of 8 wt% catalyst loading at 80 °C, Zn(**1**–**3**)_2_ were tested for PLA methanolysis in air (Table 3). Reactions were prepared in a round bottom flask and fitted with a reflux condenser prior to the reaction. Zn(**1**)_2_ proved to be most sensitive to atmospheric conditions with conversion and yield reduced from 96% and 71% to 54% and 12%, respectively. Zn(**2**)_2_ and Zn(**3**)_2_ gave relatively unchanged results. Zn(**3**)_2_ still reached full conversion after 30 min with 94% yield. The stability of Zn(**3**)_2_ in air could result from the formation of the carbamate complex, which may have a protective effect against deactivation (Figure S15, Supporting Information). Air stability is an important factor when considering the industrial viability of a catalyst, as air‐sensitive conditions can be difficult to enact at scale.

**Table 3:** Degradation of PLLA cup to methyl lactate via methanolysis using Zn(1–3)_2_ in air at 80 °C.^a)^

Cat.	Time [min]	Loading [wt%]	*Y*_MeLA_ [%]^b)^	*S*_MeLA_ [%]^b)^	*X*_int_ [%]^b)^
Zn(**1**)_2_	30	8	12	22	54
Zn(**2**)_2_	30	8	19	25	64
Zn(**3**)_2_	30	8	94	94	100

^a)^ Reaction conditions: 0.25 g of PLLA cup (*M*_n_ = 45510 g mol^−1^), *V*_THF_:*V*_MeOH_ = 4:1, *n*_MeOH_:*n*_ester_ = 7:1, 8 wt% cat. loading (20 mg, 1.4–1.6 mol% relative to ester linkages).

^b)^ ^1^H NMR (400 MHz, 298 K, CDCl_3_) spectroscopy used to calculate *Y*_MeLA_, *S*_MeLA_, and *X*_Int_.

### Kinetics of PLA Methanolysis

**Figure 1:**
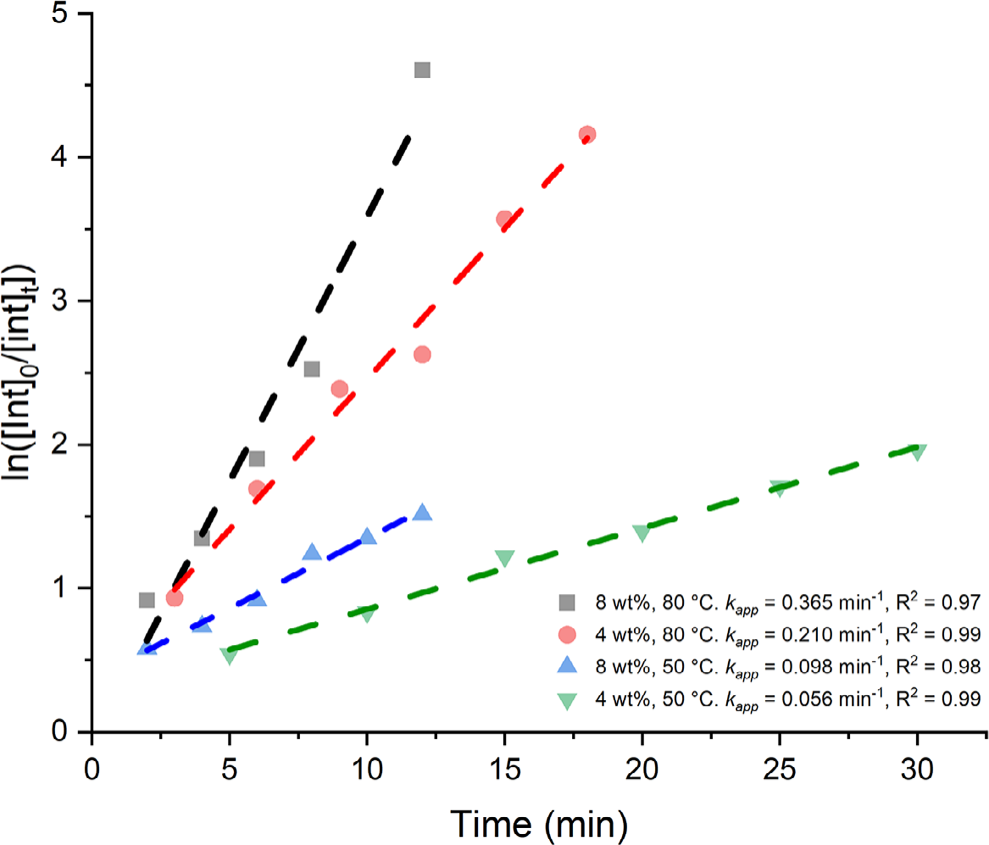
Semi‐logarithmic plot for PLA degradation with Zn(3)_2_ at various conditions.

A simple kinetic analysis was performed on the three catalysts using semi‐logarithmic plots and assuming first‐order kinetics based on previous literature (Tables 1 and 2; Figure S26, Supporting Information).^[^ ^64^, ^65^, ^68^ ^]^ This data can also be visualized as plots of conversion versus time in Figure S27 (Supporting Information). Zn(**3**)_2_ was the most active catalyst throughout, and the rate of this catalyst at various conditions can be seen in Figure 1. At 80 °C, Zn(**3**)_2_ was by far the most active catalyst at 8 wt% loading (*k*_app_ = 0.365 min^−1^); this is an order of magnitude higher than Zn(**1**)_2_ (*k*_app_ = 0.064 min^−1^) and Zn(**2**)_2_ (*k*_app_ = 0.029 min^−1^) (Figure S26A, Supporting Information). The activity of Zn(**3**)_2_ significantly outperforms recently published {ONS} and {ONO} complexes at the same conditions.^[^ ^67^, ^68^ ^]^ The trend is repeated at 4 wt% (Figure S26B, Supporting Information), with Zn(**3**)_2_ achieving an apparent rate constant of 0.210 min^−1^.

At 50 °C and 8 wt% loading (Figure S26C, Supporting Information), Zn(**2**)_2_ (*k*_app_ = 0.031 min^−1^) was more active than Zn(**1**)_2_ (*k*_app_ = 0.023 min^−1^), although the rate of Zn(**3**)_2_ was three times higher (*k*_app_ = 0.098 min^−1^). At the same conditions, Zn(**3**)_2_ is more active than previously reported half‐salan and ethylenediamine‐based phenoxy‐imine complexes.^[^ ^65^, ^71^ ^]^ A bisguanidine catalyst reported by Herres‐Pawlis and co‐workers gave similar results with roughly comparable conditions (1 mol%, 9.4 wt%, 60 °C; *k*_app_ = 0.073 min^−1^).^[^ ^76^ ^]^

When the catalyst loading was reduced by half to 4 wt% (Figure S26D, Supporting Information), the apparent rate constant for Zn(**3**)_2_ was approximately halved to 0.056 min^−1^. Although still relatively rapid, this is significantly exceeded by a propylene‐diamine‐based phenoxy imine complex (*k*_app_ = 0.20 min^−1^).^[^ ^64^ ^]^ Although not a perfect comparison, Zn(**3**)_2_ appears to be more active than a guanidine‐based, TMGeech complex for PLA methanolysis at similar conditions (1 mol%, 5.6 wt%, 60 °C; *k*_app_ = 0.0165 min^−1^).^[^ ^61^ ^]^

### Ethanolysis and Butanolysis of PLA

The most active catalysts, Zn(**1**)_2_ and Zn(**3**)_2_, were further tested for the alcoholysis of PLA to ethyl lactate and n‐butyl lactate, two of the most industrially relevant lactate esters (Table S3 and Figures S28–S30, Supporting Information).^[^ ^86^, ^87^ ^]^ As expected from the steric profiles of the alcohols, activity decreased with size (MeOH > EtOH > n‐BuOH) in accordance with previous literature.^[^ ^68^, ^76^ ^]^ This trend is supported by kinetic data with the fastest catalyst, Zn(**3**)_2_, which gave rate constants of 0.365, 0.127, and 0.073 min^−1^ when using methanol, ethanol, and n‐butanol respectively.

### Methanolysis of BPA‐PC

**Figure 2:**
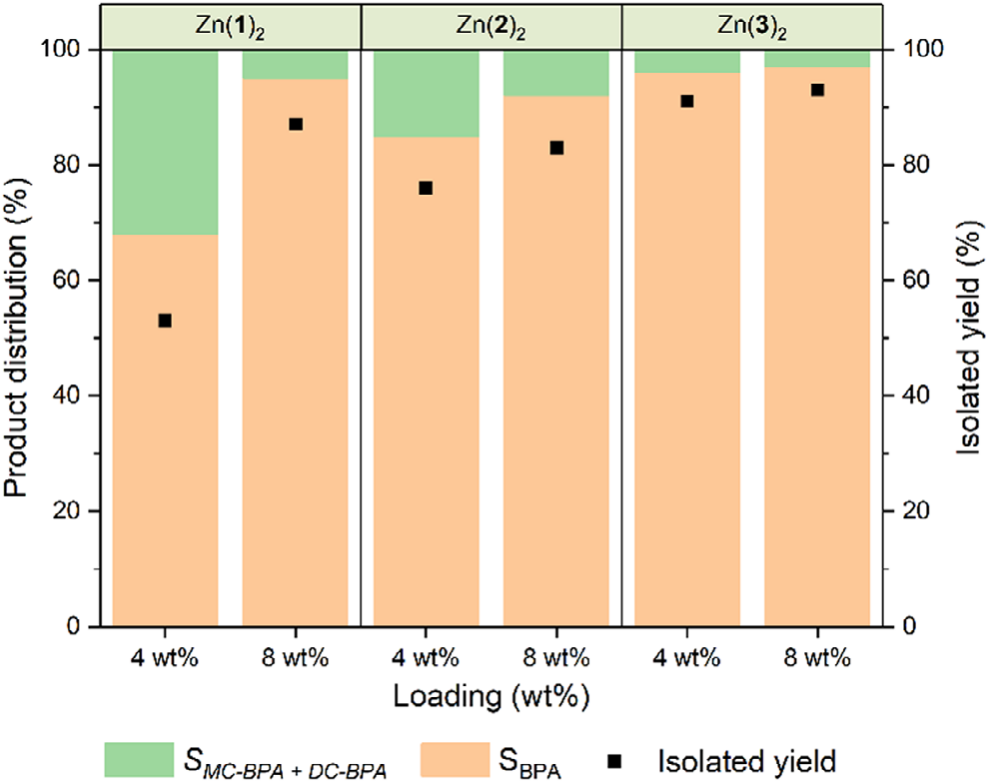
Methanolysis of BPA‐PC with Zn(**1**–**3**)_2_. Reaction conditions: 75 °C, 1 h, 0.25 g BPA‐PC pellets (*M*_n_ ≈ 45000 gmol^−1^), 17.5 equivalents of MeOH relative to carbonate linkages, 4 – 8 wt% cat. loading (10 – 20 mg, 2.4 – 5.5 mol% based on carbonate linkages). Selectivities based on ^1^H NMR spectroscopy. BPA was recrystallized from water and dried to constant weight.

The methanolysis of BPA‐PC was attempted with Zn(**1**–**3**)_2_ (Figure 2; Table S4, Supporting Information) using initial conditions optimized by Payne and co‐workers (17.5 eq. methanol, 75 °C, 1 h).^[^ ^71^ ^]^ Previous work of this kind has employed the green solvent 2‐methyl tetrahydrofuran (2‐MeTHF). However, in this case it was important to align conditions with PLA degradation for the mixed plastics experiments, and the ^1^H NMR peaks of 2‐MeTHF can interfere with PLA degradation products. The product distribution was quantified through ^1^H NMR spectroscopy using literature assignments for the methyl region of bisphenol A (BPA) and the side products: monocarbonate bisphenol A (MC‐BPA) and dicarbonate bisphenol A (DC‐BPA).^[^ ^74^ ^]^ The consumption of BPA‐PC is often quoted as a measure of activity for polycarbonate depolymerization. This can be a useful proxy when the polymer is insoluble in the reaction medium, however, the solubility of the polymer in THF suggests this might be a misleading metric. To determine whether the polymer had indeed been consumed, a final reaction mixture was spiked with a small amount of dissolved BPA‐PC (Figure S31, Supporting Information). The position of the methyl peak showed that a negligible quantity of BPA‐PC was present at the end of reaction.

Initial testing at 4 wt% loading showed that Zn(**1**)_2_ was the least selective catalyst after 1 h (*S_BPA_* = 68%) and that Zn(**3**)_2_ was the most selective (*S_BPA_* = 96%), in keeping with the degradation results for PLA. The selectivity of all catalysts increased with catalyst loading from 4 wt% to 8 wt%, with Zn(**1**)_2_, Zn(**2**)_2_, and Zn(**3**)_2_ giving BPA selectivities of 92%, 95%, and 97% respectively. BPA was recovered through crystallization from water at the end of each experiment and the same trend was observed, although yields were consistently lower than those measured spectroscopically, as expected. The activity of Zn(**3**)_2_ is similar to that reported with the most active zinc monophenolates.^[^ ^71^ ^]^ A further experiment was conducted in THF with 4 wt% Zn(**3**)_2_ at 75 °C and aliquots were taken for ^1^H NMR analysis with the goal of extracting kinetic data for comparison with the PLA methanolysis results. From the stacked spectra in Figure S34 (Supporting Information), the polymer is consumed within a few minutes, meaning that comparable kinetics could not be reliably obtained. Figure S35 (Supporting Information) shows the formation of BPA over the course of the reaction. BPA is produced rapidly up to around 85% in 15 min before plateauing at ≈95%. These results show that, during BPA‐PC methanolysis, polymer is consumed rapidly into a mixture of products which are then converted to BPA relatively slowly.

### Solvent‐Free PLA Methanolysis

A series of PLA methanolysis experiments were conducted in neat methanol using Zn(**3**)_2_. The term solvent‐free is used due to PLA, BPA‐PC, and PET being insoluble in the alcoholysis reagents. This could be advantageous as it removes the need for excessive amounts of solvent to be used and satisfies many principles of green chemistry by reducing the unnecessary use of large amounts of solvent.^[^ ^88^ ^]^ However, this approach typically requires high temperatures due to the insolubility of PLA in methanol. The reaction times were based on the disappearance of PLA; full conversion usually coincides with full solubilization of the reaction mixture. A series of control experiments were conducted, without the addition of a catalyst (Table S5, Supporting Information). No conversion of PLA was observed after 1 h at 80, 100, and 130 °C. Interestingly, PLA appeared to dissolve in methanol at 130 °C after 30 min.

The effect of temperature was initially explored using 4 wt% (0.79 mol%) catalyst (Table 4, **entry 1**–**5**). At 130 °C, full conversion was achieved in 1 min. This compares favorably to literature examples of iron and zinc guanidine catalysts from the Herres‐Pawlis group which took 1 h to reach full conversion at 150 °C and 1 mol% loading.^[^ ^89^, ^90^ ^]^ A hybrid guanidine {NO} complex from the same group was capable of 98% MeLA yield after 10 min and could be recycled four times without significant loss of activity.^[^ ^61^ ^]^ A propylene‐bridged zinc monophenolate complex also required a full hour to reach full PLA conversion; however, this experiment was conducted with a much lower loading of catalyst (1 wt%, 0.11 mol%). PLA degradation with Zn(**3**)_2_ also proceeded rapidly at 100 and 80 °C, reaching high conversion and yield in two and 11 min, respectively. The latter is significantly quicker than the equivalent reaction in THF which took 1 h to reach high conversion (Table 1). Activity was dramatically reduced at 50 and 25 °C and 24 h was required to attain moderate to high conversions. This is similar to the results seen with the aforementioned zinc carboxy guanidine which gave no conversion after 24 h at room temperature.^[^ ^90^ ^]^

**Table 4:** Solvent‐free degradation of PLLA cup to methyl lactate via methanolysis using Zn(**3**)_2_.^a)^

Entry	Atm.	Scale [g]	Loading [wt%]	Temp. [°C]	Time [min]^b)^	*Y_MeLA_* [%]^c)^	*S_MeLA_* [%]^c)^	*X_int_* [%]^c)^
1	Argon	0.25	4	130	1	100	100	100
2	Argon	0.25	4	100	2	94	94	100
3	Argon	0.25	4	80	11	98	98	100
4	Argon	0.25	4	50	24 h	96	98	98
5	Argon	0.25	4	25	24 h	30	82	36
6	Air	0.25	4	80	25	98	98	100
7	Argon	0.25	2	80	14	91	91	100
8	Argon	0.25	1	80	170	39	61	65
9	Air	10	4	80	60	99	99	100

^a)^ Reaction conditions: 0.25 g of PLLA cup (*M*_n_ = 45510 g mol^−1^), MeOH (2 mL, *n*_MeOH_ *:n*_ester_ = 14: 1), 1–4 wt% cat. loading (2.5–50 mg, 0.20–0.78 mol% relative to ester linkages).

^b)^ Times are given in minutes unless otherwise stated.

^c)^ ^1^H NMR (400 MHz, 298 K, CDCl_3_) spectroscopy used to calculate *Y*_MeLA_, *S*_MeLA_, and *X*_Int_.

Further reactions were conducted at 80 °C as it is the lowest temperature capable of high activity (Table 4 **, entry 6–9**). Conducting the reaction in air with 4 wt% catalyst resulted in full conversion after an increased reaction time of 25 min. Decreasing the loading to 2 wt% had only a small effect on activity, giving 91% MeLA yield after 14 min; however, further reducing the loading to 1 wt% resulted in PLA consumption after 170 min giving a 39% yield of MeLA.

The methanolysis reaction was scaled up to 10 g in air to further test the scalability of this process (Table 4 **, entry 9**). A longer reaction time was required (60 min) but full conversion and a 99% yield of MeLA was achieved.

The general applicability of this system was tested with a number of PLA waste sources (Table S6 and Figure S36, Supporting Information). There was very little difference in reactivity between a clear PLA cup, PLA fabric, and high molecular weight PLA pellets. This demonstrates that the rate of methanolysis is mostly independent of sample morphology. When a white 3D printing filament of PLA was tested, the reaction time increased to 150 min. To determine if this was due to its stiffness or TiO_2_, additional experiments were conducted. This effect was reduced by solvent casting the sample, at which point reaction time decreased to 40 min. However, it was difficult to tell the difference between clumps of TiO_2_ and residual polymer in the reaction mixture, so the reaction may have been quicker than the noted time. A sample of the same filament was dissolved and filtered to remove TiO_2_ and solvent cast. This resulted in full conversion after 13 min, in keeping with the other PLA sources. This suggests that a pre‐treatment step may be beneficial for the alcoholysis of crystalline PLA waste. Finally, a PLA cup was dissolved, filtered and solvent cast as a comparison with the filament. This process did not change the results. Interestingly, the methanolysis of two 3D‐printed products, a globe and a dragon, proceeded significantly faster than the unused filament, taking 27 and 13 min, respectively, to reach the high conversion. This is likely a result of reduced particle size and *M_n_* from the printing process in addition to an increased ratio of surface area to volume.

### Catalyst Reuse

**Figure 3:**
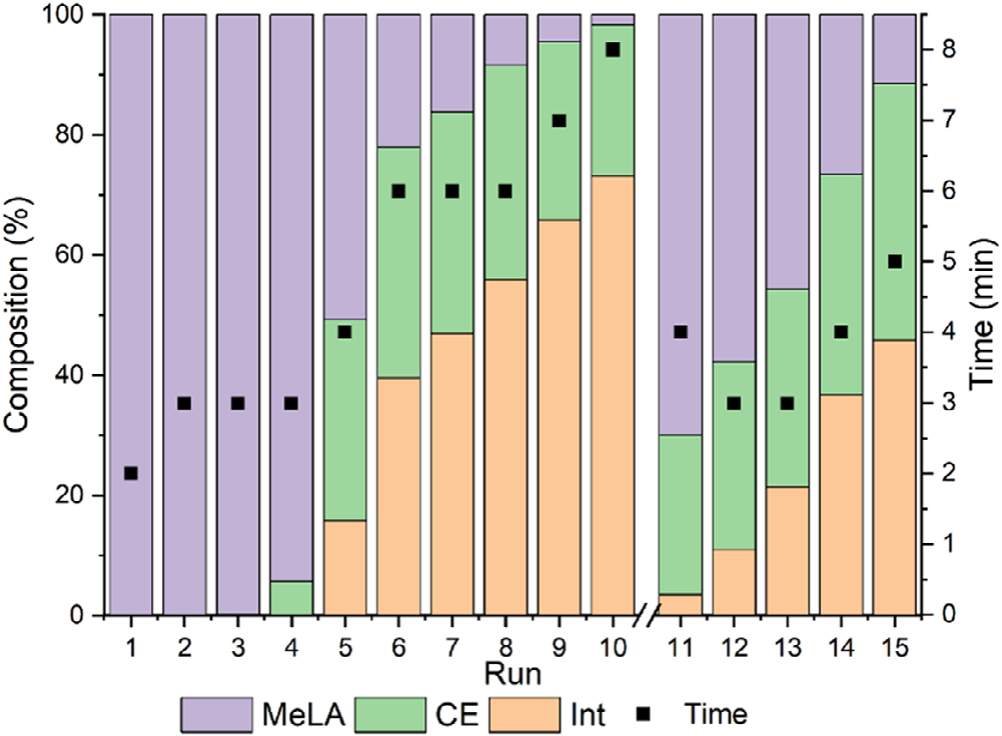
Catalyst reuse test. Reaction conditions: 0.25 g of PLLA cup (*M*_n_ = 45510 g mol^−1^), MeOH (2 mL, *n*_MeOH/_ *n*_ester_ = 14: 1), 4 wt% cat. loading (10 mg, 0.78 mol% relative to ester linkages). ^1^H NMR (400 MHz, 298 K, CDCl_3_) spectroscopy used to calculate *Y*_MeLA_, *S*_MeLA_ and *X*_Int_. After each run, volatiles were removed and a fresh portion of PLLA cup (0.25 g) and methanol (2 mL) were added. Zn(**3**)_2_ (10 mg) was added after run 10.

The recyclability of the catalyst was tested through a series of solvent‐free PLA methanolysis experiments (Figure 3 **;** Table S7, Supporting Information). A reaction flask was charged with PLA, Zn(**3**)_2_, and methanol and heated to 130 °C. Upon the disappearance of the PLA, volatiles were removed in vacuo and fresh portions of PLA and methanol were added to the flask. Initially, this was repeated ten times, followed by the addition of fresh catalyst and a further five runs.

During the first four runs, there was minimal deactivation of the catalyst giving 100% conversion in 2–3 min. Runs 5–10 showed a steady reduction in conversion and yield and a corresponding increase in reaction time. As the PLA fragments were always seen to disappear, this suggests a buildup of soluble oligomers in the reaction mixture, which could have hampered reactivity and decreased the effective catalyst loading. The chain end peaks that are a proxy for oligomer concentration only account for relatively small molecules and the increasing presence of internal methine will include oligomeric species as well as unreacted PLA. Catalyst recycling in a solvent‐free PLA ethanolysis experiment has been reported by Herres‐Pawlis and coworkers who ran four consecutive runs at 150 °C with a zinc bisguanidine catalyst.^[^ ^76^ ^]^ The activity was initially high but dropped off quickly and reached around an 80% conversion and 50% MeLA yield after four runs.

Upon addition of fresh catalyst, activity was mostly restored, giving 97% conversion and 70% yield after 4 min. This again was reduced over subsequent runs. This demonstrates that the reduction in activity cannot be solely a result of catalyst deactivation and must be influenced by the products of PLA degradation. In addition to issues posed by oligomers, there could also be an effect of residual MeLA building up over time as it has a high boiling point. This would push the equilibrium toward oligomers in the reversible second step of the established mechanism.^[^ ^91^ ^]^

Catalyst recycling was also attempted with Zn(**3**)_2_ at 80 °C (Table S8, Supporting Information). The deactivation of the system was more apparent under these conditions, and the time taken to achieve full PLA dissolution increased from 20 to 120 min in four cycles. This suggests that the catalyst is more affected by prolonged exposure to the reaction mixture than it is by temperature or the number of catalytic turnovers.

A further experiment was conducted to assess the viability of solvent reclamation and MeLA isolation (Table S9 and Figures S37–S39, Supporting Information). Due to the difficulty of distillation using small volumes, a reaction using 25 g of PLA cup with 4 wt% Zn(**3**)_2_ and 200 mL methanol was carried out and reached full conversion and 99% MeLA yield after 30 min. 119.4 g of MeOH, containing < 1% MeLA, was collected through rotary evaporation, representing 81% reclamation. A small‐scale methanolysis was carried out using this methanol and no difference in activity was observed from experiments using fresh methanol. MeLA was distilled through a Schlenk line and collected in a cold trap. A total of 26.7 g of MeLA was collected, although this contained methanol in a 3: 1 (MeLA: MeOH) molar ratio.

### Solvent‐Free BPA‐PC Methanolysis

The solvent‐free methanolysis of BPA‐PC was carried out at a range of conditions with Zn(**3**)_2_ (Table 5). A catalyst‐free control experiment at 130 °C resulted in a 62% reduction in the mass of the polymer and a 93% selectivity to BPA, giving an overall yield of 60%. The addition of 4 wt% Zn(**3**)_2_ drastically improved these results giving a 93% yield of BPA after 10 min. This is half the reaction time required for La(acac)_3_, which was shown to depolymerize BPA‐PC in 20 min at 135 °C in methanol.^[^ ^92^ ^]^ In contrast to the PLA results, reducing the temperature had a significant impact on reactivity with 50 and 120 min required for full conversion at 100 and 80 °C, respectively. The latter provides a comparison with BPA‐PC depolymerization in THF that gave high conversion and selectivity in 1 h, quicker than the solvent‐free examples, in contrast to PLA. However, the system was relatively tolerant to low catalyst loadings. Full conversion was achieved after 22 min at 2 wt% and 28 min at 1 wt%. Entries 7 and 8 show the effectiveness of Zn(**3**)_2_ with two different sources of BPA‐PC: the bottom layer of a CD and a pair of lab safety glasses. Both achieved full conversion after 9 and 6 min, demonstrating the applicability of this technique to mixed BPA‐PC waste streams. There are very few further examples of neat BPA‐PC depolymerization in the literature, including Kim et al. who achieved full BPA‐PC conversion after 6 h in a ball mill with no solvent or catalyst present.^[^ ^93^ ^]^

**Table 5:** Depolymerization of BPA‐PC to BPA via methanolysis using Zn(**3**)_2_ in neat methanol.^a)^

Entry	Source	Time [min]	Temp. [°C]	Loading [wt%]	Mass loss [%]^c)^	*S*_BPA_ [%]^b)^	*S*_DC‐BPA+MC‐BPA_ [%]^b)^	*Y_BPA_* [%]	*Y*_BPA_ (Isolated) [%]^d)^
1	Pellets	120	130	0	62	93	7	60	41
2	Pellets	10	130	4	100	97	3	97	93
3	Pellets	50	100	4	100	95	5	95	84
4	Pellets	240	80	4	100	95	5	95	90
5	Pellets	22	130	2	100	94	6	94	90
6	Pellets	28	130	1	100	96	4	96	89
7	CD	9	130	4	100	98	2	98	89
8	Goggles	6	130	4	100	99	1	99	93

^a)^ Reaction conditions: 0.25 g BPA‐PC (Pellets: *M*_n_ ≈ 45000 gmol^−1^), 2 mL MeOH. 50 equivalents of MeOH (relative to carbonate linkages), 1–4 wt% catalyst loading (2.5–10 mg, 0.70–2.9 mol% based on carbonate linkages.

^b)^ Selectivities based on ^1^H NMR spectroscopy.

^c)^ Residual BPA‐PC was collected by filtration and dried in vacuo.

^d)^ BPA was recrystallized from water and dried to constant weight.

### PET Glycolysis

All three catalysts were tested for the glycolysis of commercial PET waste to produce BHET, a common monomer for the production of virgin PET (Table 6).^[^ ^12^, ^15^ ^]^ The reactions were run with no additional solvent using literature conditions (180 °C, 20.6 eq. EG (ethylene glycol)) until the complete disappearance of the polymer was observed.^[^ ^67^, ^68^, ^71^ ^]^ An aliquot was taken and analyzed with an internal standard to give the spectroscopic BHET yield (Figure S40, Supporting Information) and the remainder was recrystallized from water to give the isolated yield of BHET (Figure S41, Supporting Information), which is typically low due to product loss during the workup procedure.^[^ ^68^ ^]^ For all catalysts, no remaining polymer was observed after 1.5 h, and the isolated yields were all below 50%. This is in line with previous reports from our group using identical conditions with monophenolate zinc complexes.^[^ ^67^, ^68^, ^71^, ^72^ ^]^ The internal standard method shows that, despite the low isolated yields, an almost complete conversion to BHET was achieved. This highlights the need for a more rigorous isolation process and could enable a kinetic analysis of PET depolymerization more easily.

**Table 6:** Depolymerization of bottle grade PET to BHET via glycolysis using Zn(**1**–**3)**_2_ at 180 °C.^a)^

Cat.	Time [h]	Loading [wt%]	*Y*_BHET_ (Isolated) [g] [(%)]^b)^	*Y*_BHET_ (^1^H NMR) [%]^c)^
Zn(**1**)_2_	1.5	8	0.15 (45%)	95
Zn(**2**)_2_	1.5	8	0.16 (49%)	94
Zn(**3**)_2_	1.5	8	0.14 (42%)	99

^a)^ Reaction conditions: 0.25 g of bottle‐grade PET (*M*_n_ ≈ 40 000 g mol^−1^), 20.6 equivalents of EG (relative to ester linkages), 8 wt% cat. loading (20 mg, 3.5–4.1 mol% relative to ester linkages).

^b)^ BHET recrystallized from water and dried in vacuo.

^c)^ Calculated using trimethoxybenzene internal standard.

### Mixed Polymer Degradation in Solution

The degradation of mixed plastic waste is a much more realistic model of the commercial applicability of a catalyst. Zn(**3**)_2_ was tested for the sequential degradation of two‐component mixtures of PLA, BPA‐PC, and PET (Table 7). Conditions were initially selected based on previous literature and the conditions optimized for this catalyst.^[^ ^68^ ^]^ A PLA/PET mixture was sequentially degraded with PLA reaching full conversion after 30 min in THF followed by the removal of volatiles and full PET consumption after 1 h under glycolysis conditions. This is significantly quicker than the monophenolate zinc {ONO} complex reported by the Jones group.^[^ ^68^ ^]^

**Table 7:** Selective and sequential degradation of mixed polymers with Zn(3)_2_.

	Polymer		Conditions		PET		PLA			BPA‐PC	
	A	B	T [°C]	T [min]	*X_PET_* [%]^d)^	Y_BHET_ [%]^e)^	*X*_int_ [%]^f)^	*S*_Me‐LA_ [%]^f)^	*Y*_MeLA_ [%]^f)^	*X*_BPA‐PC_ [%]^d)^	*Y_BPA_* [g]^g)^
1^a)^	PLA	PET	A) 80 B) 180	30 60	‐ 100	‐ 92	100 ‐	88 ‐	88 ‐		
2^b)^	BPA‐PC	PET	A) 80 B) 180	60 60	‐ 100	‐ 75				100 ‐	93 ‐
3^c)^	PLA	BPA‐PC	A) 50 B) ‐	30 ‐			78 ‐	40 ‐	31 ‐	100 ‐	40 ‐

^a)^ Reaction conditions: 0.25 g of PLLA cup (*M*_n_ = 45 510 g mol^−1^), 0.25 g of bottle‐grade PET (*M*_n_ ≈ 40 000 g mol^−1^), THF (4 mL), MeOH (0.5 mL), 4 wt% Zn(**3**)_2_ loading relative to PLA (10 mg). After 1A, volatiles were removed, and an aliquot taken for ^1^H NMR analysis before applying conditions 1B with EG (2 mL).

^b)^ Reaction conditions: 0.25 g BPA‐PC pellets (*M*_n_ ≈ 45 000 gmol^−1^), 0.25 g of bottle‐grade PET (*M*_n_ ≈ 40 000 g mol^−1^), THF (4 mL) MeOH (0.5 mL), 4 wt% Zn(**3**)_2_ loading relative to BPA‐PC (10 mg). After 3A, an aliquot was taken for ^1^H NMR analysis before applying conditions 3B.

^c)^ Reaction conditions: 0.25 g of PLLA cup (*M*_n_ = 45 510 g mol^−1^), 0.25 BPA‐PC pellets (*M*_n_ ≈ 45 000 gmol^−1^), THF (4 mL), MeOH (0.5 mL), 4 wt% Zn(**3**)_2_ loading relative to PLA (10 mg).

^d)^ Mass loss of original polymer.

^e)^ Spectroscopic yield of BHET using trimethoxybenzene internal standard.

^f)^ Determined by ^1^H NMR analysis of methine protons.

^g)^ Calculated from ^1^H NMR spectroscopy.

BPA‐PC depolymerization gave a 93% BPA yield after 1 h in THF in the presence of PET. After the removal of volatiles, full PET consumption was observed in 1 h. Wang and coworkers removed BPA from the reaction through filtration in methanol.^[^ ^79^ ^]^ One issue with this approach is it necessitates the addition of fresh catalyst and so BPA was left in the flask during glycolysis in this case.

The sequential degradation of PLA and BPA‐PC is challenging as they have similar reactivity in these conditions. Some approaches to overcoming this issue include changing the catalyst for each component or using a “one pot” strategy with a product separation step.^[^ ^81^, ^93^ ^]^ A systematic series of experiments was carried out with the goal of attaining selectivity between the methanolysis of each component (Table S10, Supporting Information). However, after optimization, there was not sufficient selectivity to make this a viable approach to selective PLA/BPA‐PC degradation in solution.

### Solvent‐Free Mixed Polymer Degradation

The solution‐based mixed plastic experiments worked well for mixtures containing PET but were not able to resolve PLA and BPA‐PC to give selective catalysis. Therefore, a series of mixed polymer experiments were conducted with no solvent (Table 8) using the conditions previously optimized for Zn(**3**)_2_ (Tables 4 and 5). These results showed that PLA was fully converted after 11 min at 80 °C, whereas BPA‐PC required 4 h at the same temperature. This suggested that sequential PLA/BPA‐PC degradation should be possible. The first polymer combination tested was PLA/PET. Methanolysis was chosen for PET to reduce the number of chemicals required, and there is the potential to recycle the solvent between steps. PLA was converted at 80 °C in 15 min, all volatiles were removed, fresh methanol was added, and PET was completely consumed in 2 h.

**Table 8:** Solvent‐free selective and sequential degradation of mixed polymers with Zn(**3**)_2_.

	Polymer		Conditions		PET		PLA			BPA‐PC	
	A	B	T [°C]	t [min]	*X_PET_* [%]^e)^	*Y*_DMT_ [%]^f)^	*X*_int_ [%]^g)^	*S*_Me‐LA_ [%]^g)^	*Y*_MeLA_ [%]^g)^	*X*_BPA‐PC_ [%]^e)^	*Y_BPA_* [g]^h)^
1^a)^	PLA	PET	A) 80 B) 130	15 120	‐ 100	‐ 78	98 ‐	70 ‐	69 ‐		
2^b)^	BPA‐PC	PET	A) 130 B) 130	11 150	‐ 52	‐ 29				100 ‐	96 ‐
3^c)^	PLA	BPA‐PC	A) 80 B) 130	11 15			100 ‐	100 ‐	100 ‐	n.d.^i)^ 100	7 97
4^d)^	PLA	BPA‐PC	A) 80 B) 130	12 18			100 ‐	97 ‐	97 ‐	n.d.^i)^ 100	6 99

^a)^ Reaction conditions: 0.25 g of PLLA cup (*M*_n_ = 45 510 g mol^−1^), 0.25 g of bottle‐grade PET (*M*_n_ ≈ 40 000 g mol^−1^), MeOH (2 mL), 4 wt% Zn(**3**)_2_ loading relative to PLA (10 mg). After 1A, volatiles were removed, and an aliquot taken for ^1^H NMR analysis before applying conditions 1B with fresh methanol (2 mL).

^b)^ Reaction conditions: 0.25 g BPA‐PC pellets (*M*_n_ ≈ 45 000 gmol^−1^), 0.25 g of bottle‐grade PET (*M*_n_ ≈ 40 000 g mol^−1^), MeOH (2 mL), 4 wt% Zn(**3**)_2_ loading relative to BPA‐PC (10 mg). After 3A, an aliquot was taken for ^1^H NMR analysis before applying conditions 3C.

^c)^ Reaction conditions: 0.25 g of PLLA cup (*M*_n_ = 45510 g mol^−1^), 0.25 BPA‐PC pellets (*M*_n_ ≈ 45 000 gmol^−1^), MeOH (2 mL), 4 wt% Zn(**3**)_2_ loading relative to PLA (10 mg). After 2A, volatiles were removed, and an aliquot taken for ^1^H NMR analysis before applying conditions 2B with fresh methanol (2 mL).

^d)^ Reaction conditions: 5 g of PLLA cup (*M*_n_ = 45 510 g mol^−1^), 5 g BPA‐PC safety glasses, MeOH (40 mL), 4 wt% Zn(**3**)_2_ loading relative to PLA (200 mg). After 4A, volatiles were removed, and an aliquot taken for ^1^H NMR analysis before applying conditions 2B with fresh methanol (40 mL).

^e)^ Mass loss of original polymer.

^f)^ Isolated yield of DMT.

^g)^ Determined by ^1^H NMR analysis of methine protons.

^h)^ Calculated from ^1^H NMR spectroscopy.

^i)^ Unable to calculate conversion by mass loss.

Although the PLA reaction was quicker than in solution, PET methanolysis took twice as long as the equivalent glycolysis, most likely due to reaction temperature. Similar results were obtained with a BPA‐PC/PET mixture, although only 52% conversion of PET was possible in 2.5 h. This indicates an inhibitive effect of the residual BPA. Selectivity in this case was based only on reaction time due to safety concerns around increasing the temperature of methanol beyond 130 °C.

**Figure 4:**
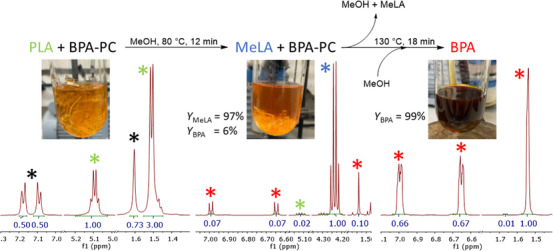
Selective and sequential methanolysis of PLA/BPA‐PC with Zn(3)_2_ in methanol.

The combination of BPA‐PC and PLA was selectively degraded in sequence through modulation of temperature. PLA reached full conversion in 11 min with a small amount of either BPA or BPA‐PC dissolved in the reaction mixture (6%). Upon removal of volatiles and the addition of fresh methanol, BPA‐PC was converted to 97% BPA in 15 min at 130 °C. This reaction was scaled up to ten grams of total polymer (5 g PLA cup, 5 g BPA‐PC safety glasses) with only a small reduction in activity, likely related to less optimal stirring (Figure 4). This result is quicker and more selective than recently published methods using ionic liquids and mechanochemical apparatus,^[^ ^82^, ^93^ ^]^ while requiring a single, reusable catalyst with an easy separation step.

## Conclusion

Three imino‐pyrrole {NNN} zinc catalysts were prepared and characterized in solution and in the solid state. Zn(**1**–**3**)_2_ were highly active for the methanolysis of PLA in THF, with apparent rates as high as 0.365 min^−1^, competitive with the most active literature examples. The rapid methanolysis of PLA was also demonstrated in neat methanol under mild conditions. Zn(**3**)_2_ was shown to work in air with minimal loss of activity, and this was linked to the crystal structure of a CO_2_ adduct. BPA‐PC was also depolymerized in solvated and solvent‐free conditions giving high yields of BPA. PET glycolysis reached high conversion in 1.5 h for all catalysts. The degradation of mixed polymers was investigated, including a rare example of the selective and sequential degradation of PLA and BPA‐PC using a single catalyst and solvent, up to a 10 g scale. This work represents a new class of highly active catalysts for chemical recycling without the requirement of extra solvents and the selective and sequential degradation of a challenging mixed polymer sample.

## Author Contributions

J.A.S. performed conceptualization, supervision, validation, investigation, writing — original draft, writing — review and editing. L.T.W.P. performed investigation, writing — original draft. M.J.C. performed investigation, supervision, writing — review and editing. G.K.‐K. performed formal analysis. M.G.D. performed supervision and acquired resources. M.D.J. performed conceptualization, resources, formal analysis, supervision, funding acquisition, project administration, and writing — review and editing.

## Conflict of Interests

The authors declare no conflict of interest.

## Supporting information



Supporting Information

## Data Availability

The data that support the findings of this study are available in the supplementary material of this article.
