# Supplementary material for: Imino‐Pyrrole Zn(II) Complexes for the Rapid and Selective Chemical Recycling of Commodity Polymers
Source: Angew Chem Int Ed Engl. 2025 Apr 2;64(22):e202502845. doi: 10.1002/anie.202502845 (PMC12105715; doi:10.1002/anie.202502845)
Supplement: Supplementary file 1 — Supporting Information [file ANIE-64-e202502845-s001.pdf]

# Supporting information

## Imino-Pyrrole Zn(II) Complexes for the Rapid and Selective Chemical Recycling of Commodity Polymers

Jack A. Stewart,[a] Louis T. W. Powell,[a] Matthew J. Cullen,[a,b] Gabrielle Kociok-Köhn,[a] Matthew G. Davidson[a,b] and Matthew D. Jones*[a,b]

a Department of Chemistry, University of Bath, Claverton Down, Bath, United Kingdom, BA2 7AY. E-mail: mj205@bath.ac.uk
b Institute of Sustainability and Climate Change, University of Bath, Claverton Down, Bath, United Kingdom, BA27AY

1. General Considerations and Procedures ........ 3

1.1 Chemicals and equipment ........ 3

1.2 General procedures ........ 3

2. Synthesis and Characterisation ........ 6

2.1 Ligand synthesis and characterisation ........ 6

2.2 Complex synthesis and characterisation ........ 10

2.3 Crystallography Discussion ........ 15

2.4 Air sensitiviy tests ........ 17

3. Lactide Polymerisation ........ 19

3.1 Solvent-free lactide ROP ........ 19

3.2 Selected lactide conversion examples ........ 19

3.3 Selected GPC traces ........ 20

4. Solution alcoholysis of PLA and BPA-PC ........ 21

4.1 PLA methanolysis – conversion, selectivity and yield ........ 21

4.2 PLA methanolysis – pseudo-First Order Kinetics and Conversion vs. Time plot ........ 23

4.3 Ethanolysis and butanolysis of PLA ........ 24

4.4 Methanolysis of BPA-PC ........ 26

5. Solvent-free alcoholysis of polymers ........ 30

5.1 Solvent-free PLA methanolysis ........ 30

5.2 Catalyst recycling ........ 31

5.3 Solvent and Product Isolation ........ 32

5.3 PET glycolysis ........ 34

6 Mixed polymer degradation ........ 35

6.1 Solution methanolysis of PLA/BPA-PC ........ 35

6.2 Example mixed polymer $^{1}$H NMR sepctra ........ 35

7. Crystallography data ........ 39

8. References ........ 39

## 1. General Considerations and Procedures

*1.1 Chemicals and equipment*

All chemicals were obtained commercially from Sigma-Aldrich and used as received. An exception was the *L*-lactide, which was singly recrystallised from anhydrous toluene prior to use. All ligands were prepared using standard literature procedures in air. All metal complexes were synthesised under an inert atmosphere (argon) using standard Schlenk line techniques, dry solvents, and oven-dried glassware. Complexes were stored in a MBraun LABmaster dp glovebox (argon) prior to use or transport.

$^{1}H$ and $^{13}C\{^{1}H\}$ NMR spectra of ligands, complexes, polymerisations, and degradations were obtained on a Bruker 400 MHz or 500 MHz instrument. Coupling constants (*J*) are provided in Hertz (Hz) and the following abbreviations were used to report the spectra: (s) singlet, (d) doublet, (dd) doublet of doublets, (t) triplet, (p) pentet, (m) multiplet. Characterisation of ligands and metal complexes used $C_6D_6$ or $CDCl_3$ as the NMR solvent. Quantification of methine regions for polymerisations and PLA degradations used $CDCl_3$ or $C_6D_6$ as the NMR solvent. NMR solvent DMSO-$d_6$ was used to characterise BHET.

Electrospray ionisation mass spectrometry (ESI-MS) was recorded for all ligands using Agilent 6545 ESI quadrupole time-of-flight, fitted with liquid chromatography module. Each spectrum was recorded in positive loop injection mode. Sample was dissolved in methanol at approximately 1 µg $mL^{-1}$.

Crystallographic data for three complexes were collected on a SuperNova or Excalibur, EOS detector diffractometer using Cu-K$\alpha$ (λ = 1.54184 Å) or Mo-K$\alpha$ (λ = 0.71073 Å) radiation, all recorded at 150(2) K. Both structures were solved by direct methods and refined on all $F^2$ data using the SHELXL-2014 suite of programs.

GPC was carried out on an Agilent 1260 Infinity series instrument at 1 mL $min^{-1}$ at 35 °C with a THF eluent using a PL gel 5 µm MIXED-D 300 x 7.5 mm column. Detection was carried out using a differential refractive index detector (referenced to 11 polystyrene standards of narrow molecular weight, ranging from $M_w$ 615 – 568000 Da).

*1.2 General procedures*

Typical lactide polymerisation procedure: *L*-Lactide (1.0 g, 6.94 × $10^{-3}$ mol) was added to a Young's ampoule with initiator (2.31 × $10^{-5}$ mol) and benzyl alcohol (2.31 × $10^{-5}$) in a glovebox. An oil bath was heated to the appropriate temperature and the polymerisation ran for one hour or until stirring was significantly slowed. Once complete, a suitable solvent was added to dissolve the solid reaction mixture. The solvent was removed *in vacuo* and the crude product analysed via $^{1}H$ NMR. The pure polymer was obtained by washing with > 30 mL methanol and used for GPC analysis.

Typical PLA alcoholysis in THF procedure: A Young's ampoule containing PLA (0.25 g, Vegware™, PLLA cup, $M_n$ = 45,510 g $mol^{-1}$), was taken into a glovebox and loaded with metal complex (4 – 8 wt%, 0.01 – 0.02 g). The polymer was then dissolved in THF (4 mL) with heating and stirring assisting dissolution. The flask was then submerged in a preheated oil bath (50 – 80 °C) to which MeOH (1 mL, 7 eq. with respect to ester group) was added. Aliquots were taken for $^{1}H$ NMR ($CDCl_3$) analysis of the methine region.
For reactions using ethanol and n-butanol, the 7 : 1 molar ratio of alcohol to ester group and the 4 : 1 volume ratio of THF to alcohol were maintained.
Reactions in air were performed in a round bottom flask with a reflux condenser attached using the conditions described above.
For kinetics experiments, aliquots were taken at the appropriate timepoints and analysed as described above.

Typical BPA-PC methanolysis in THF procedure: A Young's ampoule containing BPA-PC pellets (0.25 g) was taken into a glovebox and loaded with metal complex (4 – 8 wt%, 0.01 – 0.02 g). The polymer was then dissolved in THF (4 mL) with heating and stirring assisting dissolution. The flask was then submerged in a preheated oil bath (75 °C) to which MeOH (17.5 eq. with respect to carbonate groups) was added. Aliquots were taken for $^1H$ NMR ($CDCl_3$) of the methyl region. BPA was recrystallised from water and dried to a constant weight to determine isolated yields.

Typical solvent-free PLA methanolysis procedure: A Young's ampoule containing PLA (0.25 g, Vegware™, PLLA cup, $M_n$ = 45,510 g mol$^{-1}$), was taken into a glovebox and loaded with metal complex (1 – 4 wt%, 0.0025 – 0.01 g). The flask was then submerged in a preheated oil bath (25 – 130 °C) to which MeOH (2 mL, 14 eq. with respect to ester group) was added. When the PLA had been fully consumed, a suitable solvent was added to ensure dissolution of the mixture, and an aliquot was taken for $^1H$ NMR ($CDCl_3$) analysis of the methine region.
Reactions in air were performed in a round bottom flask with a reflux condenser attached using the conditions described above.

Typical solvent-free BPA-PC methanolysis procedure: A Young's ampoule containing BPA-PC pellets (0.25 g) was taken into a glovebox and loaded with metal complex (1 – 4 wt%, 0.0025 – 0.01 g). The flask was then submerged in a preheated oil bath (80 – 130 °C) to which MeOH (2 mL, 50 eq. with respect to carbonate group) was added. When the PLA had been fully consumed, a suitable solvent was added to ensure dissolution of the mixture and an aliquot was taken for $^1H$ NMR ($CDCl_3$) analysis of the methine region. Aliquots were taken for $^1H$ NMR ($CDCl_3$) of the methyl region. BPA was recrystallised from water and dried to a constant weight to determine isolated yields.

Typical PET glycolysis procedure: A Young's ampoule containing PET (0.25 g of carbonated drinks bottle), was taken into a glovebox and loaded with metal complex (8 wt%, 0.02 g). Ethylene glycol (EG) (27.5 eq., 1.5 mL) was added, and the flask was submerged in a pre-heated oil bath at 180 °C. When full disappearance of the PET was observed, water was added, and the mixture was filtered. BHET crystallised from the mixture and was collected, dried at 100°C *in vacuo* for 4 hours and weighed to obtain isolated yields. When required, an aliquot was taken and analysed with a 1,3,5-trimethoxybenzene internal standard to obtain spectroscopic BHET yield.

Typical mixed-polymer degradation in THF procedure: PLA/PET: A Young's ampoule containing 0.25 g each of the relevant polymers was taken into the glovebox and loaded with metal complex (4 wt% with respect to one component, 10 mg). PLA was then dissolved in THF (4 mL) with heating and stirring assisting dissolution. The ampoule was submerged in a pre-heated oil bath (80 °C) to which MeOH (2 mL, 14 eq. with respect to ester group) was added. An aliquot was taken for $^1H$ NMR analysis and volatiles were removed. EG (2 mL) was added, and the reaction was heated to 180 °C until PET had been consumed.

PLA/BPA-PC: A Young's ampoule containing 0.25 g each of the relevant polymers was taken into the glovebox and loaded with metal complex (2 – 8 wt% with respect to one component, 5 – 20 mg). The polymers were then dissolved in THF (4 mL) with heating and stirring assisting dissolution. The ampoule was submerged in a pre-heated oil bath (30 – 80 °C) to which MeOH (5 – 15 eq. with respect to ester + carbonate groups. An aliquot was taken for $^1H$ NMR analysis.

BPA-PC/PET: A Young's ampoule containing 0.25 g each of the relevant polymers was taken into the glovebox and loaded with metal complex (4 wt% with respect to one component, 10 mg). BPA-PC was then dissolved in THF (4 mL) with heating and stirring assisting dissolution. The ampoule was submerged in a pre-heated oil bath (80 °C) for 60 minutes. An aliquot was taken for $^1H$ NMR analysis and volatiles were removed. EG (2 mL) was added, and the reaction was heated to (180 °C) until PET had been consumed.

Typical solvent-free mixed-polymer degradation procedure:

PLA/PET: A Young's ampoule containing 0.25 g each of the relevant polymers was taken into the glovebox and loaded with metal complex (4 wt% with respect to one component, 10 mg). The ampoule was submerged in a pre-heated oil bath (80 °C) to which MeOH (2 mL, 14 eq. with respect to PLA ester group) was added. An aliquot was taken for $^1$H NMR analysis and volatiles were removed. MeOH (2 mL) was added, and the reaction was heated to 130 °C until PET had been consumed.

PLA/BPA-PC: A Young's ampoule containing 0.25 g each of the relevant polymers was taken into the glovebox and loaded with metal complex (4 wt% with respect to one component, 10 mg). The ampoule was submerged in a pre-heated oil bath (80 °C) to which MeOH (2 mL, 14 eq. with respect to PLA ester group) was added. Upon disappearance of PLA, an aliquot was taken for $^1$H NMR analysis and volatiles were removed. MeOH (2 mL) was added, and the reaction was heated to 130 °C until BPA-PC had been consumed. A further aliquot was taken for $^1$H NMR analysis.

BPA-PC/PET: A Young's ampoule containing 0.25 g each of the relevant polymers was taken into the glovebox and loaded with metal complex (4 wt% with respect to one component, 10 mg). The ampoule was submerged in a pre-heated oil bath (130 °C) to which MeOH (2 mL)was added. An aliquot was taken for $^1$H NMR analysis and volatiles were removed. MeOH (2 mL) was added, and the reaction was heated to 130 °C until PET had been consumed.

## 2. Synthesis and Characterisation

### *2.1 Ligand synthesis and characterisation*

### **1**H

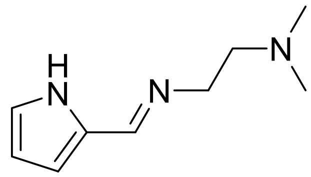

Pyrrole-2-carboxaldehyde (0.95 g, 10 mmol) and N, N-dimethyl ethylenediamine (1.09 mL, 10 mmol) were added to a round bottomed flask with MeOH (30 mL). The reaction was stirred at room temperature for 24 h and the solution went from colourless to orange. The solvent was then removed *in vacuo* leaving a dark red oil as the product, **1**H (1.55 g, 94%).

$^{1}$H NMR (400 MHz, $C_6D_6$) δ 7.79 (s, 1H, **H**C=N), 6.39 (dd, *J* = 3.6, 1.4 Hz, 1H, **H**Ar), 6.34 (t, *J* = 1.8 Hz, z1H, **H**Ar), 6.18 (dd, *J* = 3.5, 2.7 Hz, 1H, **H**Ar), 3.52 (td, *J* = 6.8, 1.3 Hz, 2H, C**H**$_2$), 2.53 (t, *J* = 6.8 Hz, 2H, C**H**$_2$), 2.14 (s, 6H, N(C**H**$_3$)$_2$).

$^{13}$C{$^{1}$H} NMR (101 MHz, $C_6D_6$) δ 151.7 (**C**=N), 130.6 (Ar**C**), 121.2 (Ar**C**), 113.5 (Ar**C**), 109.4 (Ar**C**), 60.5 (**C**H$_2$), 59.0 (**C**H$_2$), 45.5 (N(**C**H$_3$)$_2$).

HRMS (ESI$^+$) *m/z*: [M+H]$^+$ calc. for $C_9H_{15}N_3$ 166.1344, found 166.1338.

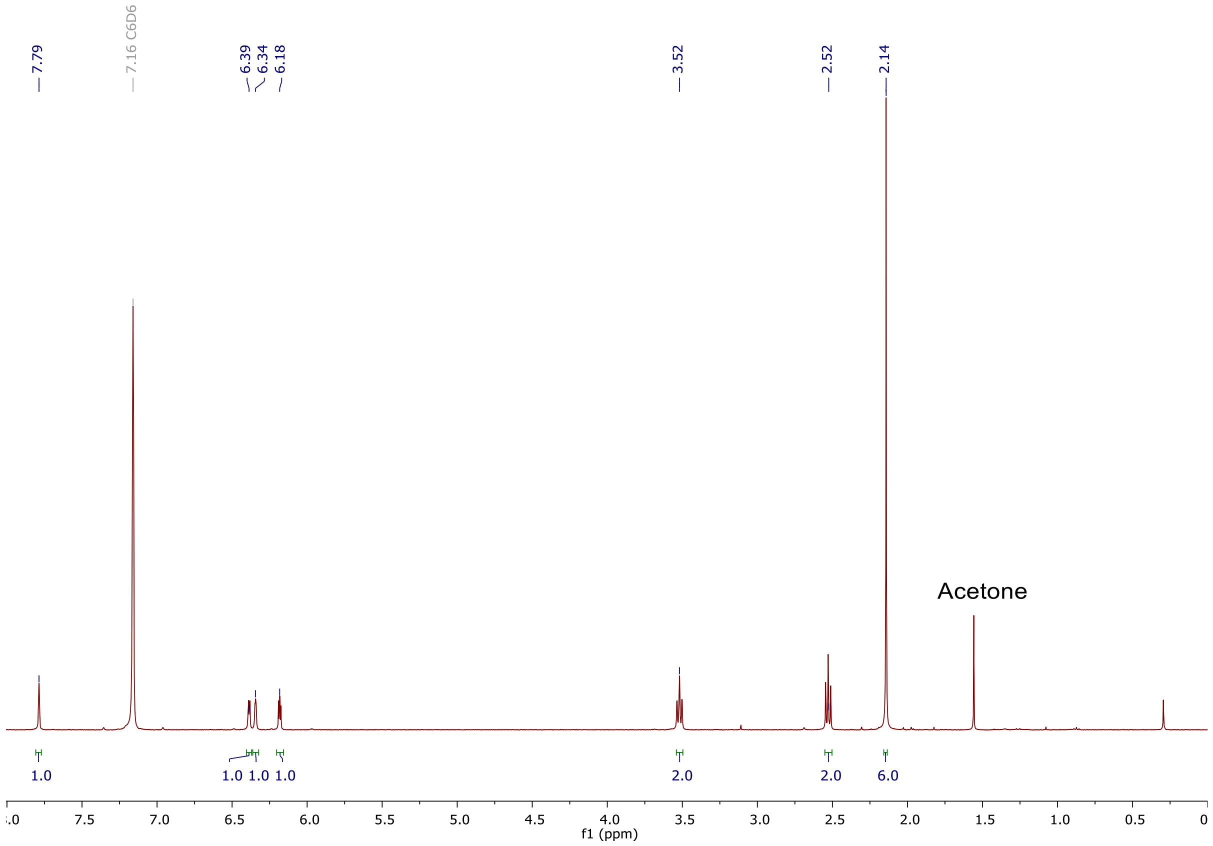


**Figure S1** $^{1}$H NMR (400 MHz, $C_6D_6$) spectrum of **1**H.

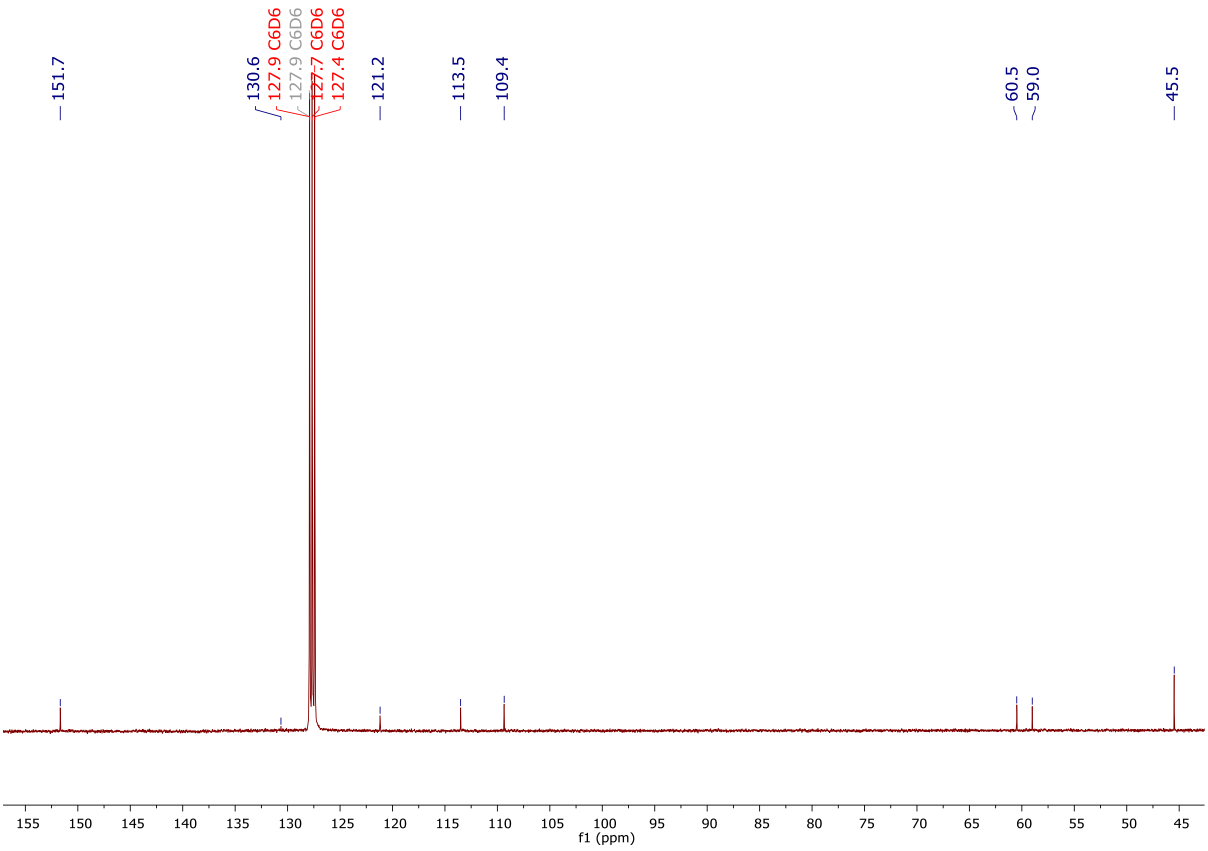


**Figure S2** $^{13}C\{^{1}H\}$ NMR (101 MHz, $C_6D_6$) spectrum of **1**H.

## **2**H

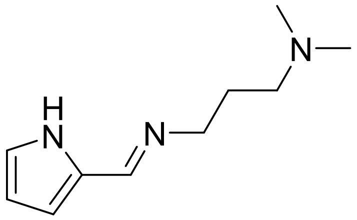

Pyrrole-2-carboxaldehyde (951 mg, 1 equiv, 10 mmol) and 3-(Dimethylamino)-1-propylamine (1.25 mL, 1 equiv, 10 mmol) were added to a round bottomed flask with MeOH (30 mL). The reaction was stirred at room temperature for 24 h and the solution went from colourless to green. The solvent was then removed *in vacuo* leaving a dark brown oil as the product, **2**H (1.66 g, 93%).

$^{1}H$ NMR (500 MHz, $CDCl_3$) δ 7.95 (s, 1H, **H**C=N), 7.96 – 7.95 (m, 1H, **H**Ar), 6.94 – 6.63 (m, 1H, **H**Ar), 6.36 (dd, *J* = 3.6, 1.4 Hz, 1H, **H**Ar), 6.14 (dd, *J* = 3.6, 2.6 Hz, 1H, **H**Ar), 3.44 (td, *J* = 6.9, 1.3 Hz, 2H, C**H**$_2$), 2.36 – 2.16 (m, 2H, C**H**$_2$), 2.13 (s, 6H, N(C**H**$_3$)$_2$), 1.71 (p, *J* = 7.1 Hz, 2H, C**H**$_2$).

$^{13}C$ NMR (126 MHz, $CDCl_3$) δ 202.4 (**C**=N), 181.1 (Ar**C**), 172.3 (Ar**C**), 164.6 (Ar**C**), 160.52 (Ar**C**), 109.7 (**C**H$_2$), 108.4 (**C**H$_2$), 96.4 (N(**C**H$_3$)$_2$), 80.1 (**C**H$_2$).

HRMS (ESI$^+$) *m/z*: $[M+H]^+$ calc. for $C_{10}H_{17}N_3$ 180.1501, found 180.1495.

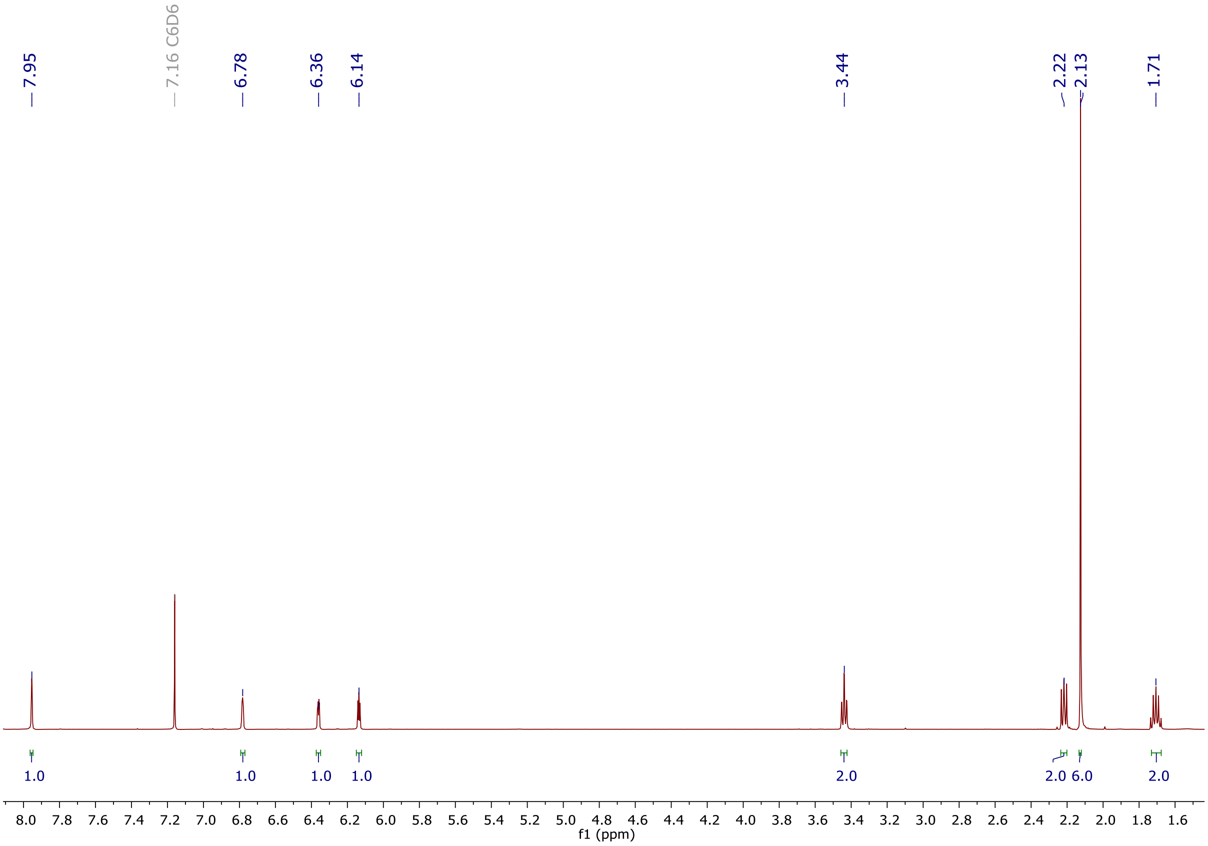


**Figure S3** $^{1}$H NMR (500 MHz, $C_6D_6$) spectrum for **2**H.

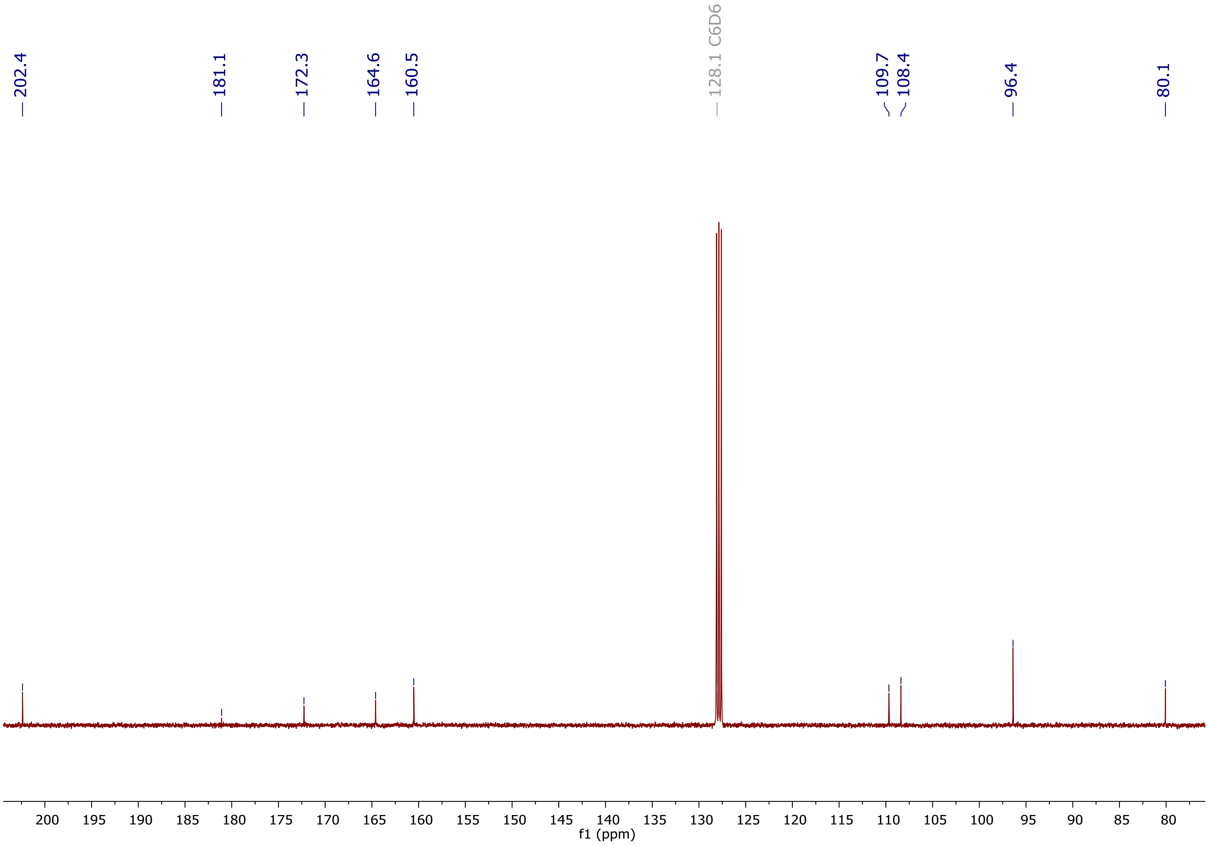


**Figure S4** $^{13}$C{$^{1}$H} NMR (126 MHz, $C_6D_6$) spectrum for **2**H.

**3**H

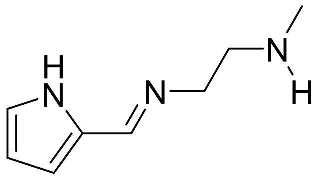

Pyrrole-2-carboxaldehyde (951 mg, 1 equiv, 10 mmol) and *N*-methyl ethylenediamine (0.871 mL, 1 equiv, 10 mmol) were added to a round bottomed flask in MeOH (30 mL). The reaction was stirred at room temperature for 24 h and the solution went from colourless to orange. The solvent was then removed *in vacuo* leaving a red oil as the product, **3**H (1.48 g, 98%).

$^1$H NMR (400 MHz, $CDCl_3$) δ 7.98 (s, 1H, **H**C=N), 6.85 – 6.71 (m, 1H, **H**Ar), 6.47 – 6.31 (m, 1H, **H**Ar), 6.13 (m, 1H, **H**Ar)), 3.60 (m, 2H, C**H**$_2$), 3.55 – 3.31 (m, 2H ,C**H**$_2$), 3.27 (s, 1H), 3.27 (d, *J* = 0.7 Hz, 3H C**H**$_3$).

$^{13}$C{$^1$H} NMR (126 MHz, $C_6D_6$) δ 152.6 (**C**=N), 130.5 (Ar**C**), 121.8 (Ar**C**), 114.5 (Ar**C**), 109.4 (Ar**C**), 60.0 (**C**H$_2$), 52.5 (**C**H$_2$), 35.7(N**C**H$_3$).

HRMS (ESI)$^+$ *m/z*: [M+H]$^+$ calc. for $C_9H_{13}N_3$ 152.1188, found 152.1180.

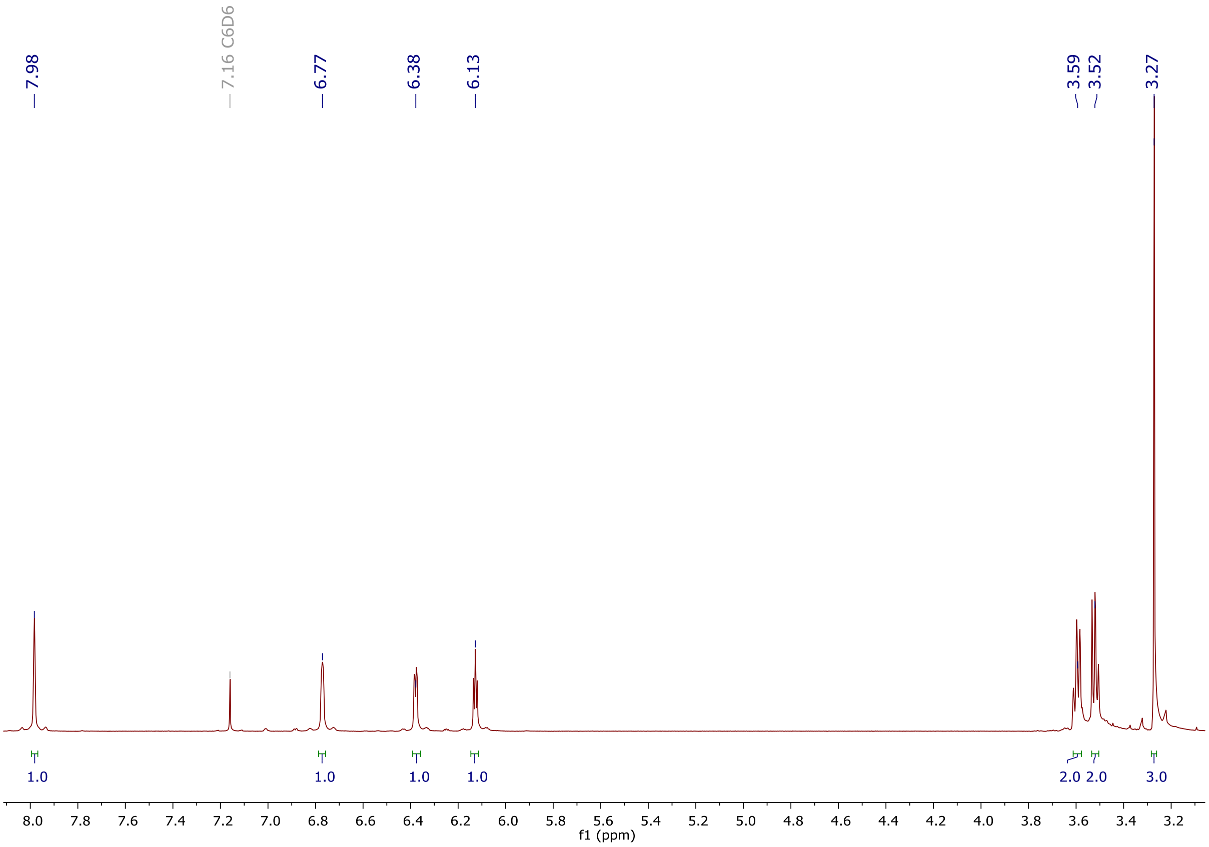

**Figure S5** $^1$H NMR (400 MHz, $C_6D_6$) spectrum for **3**H.

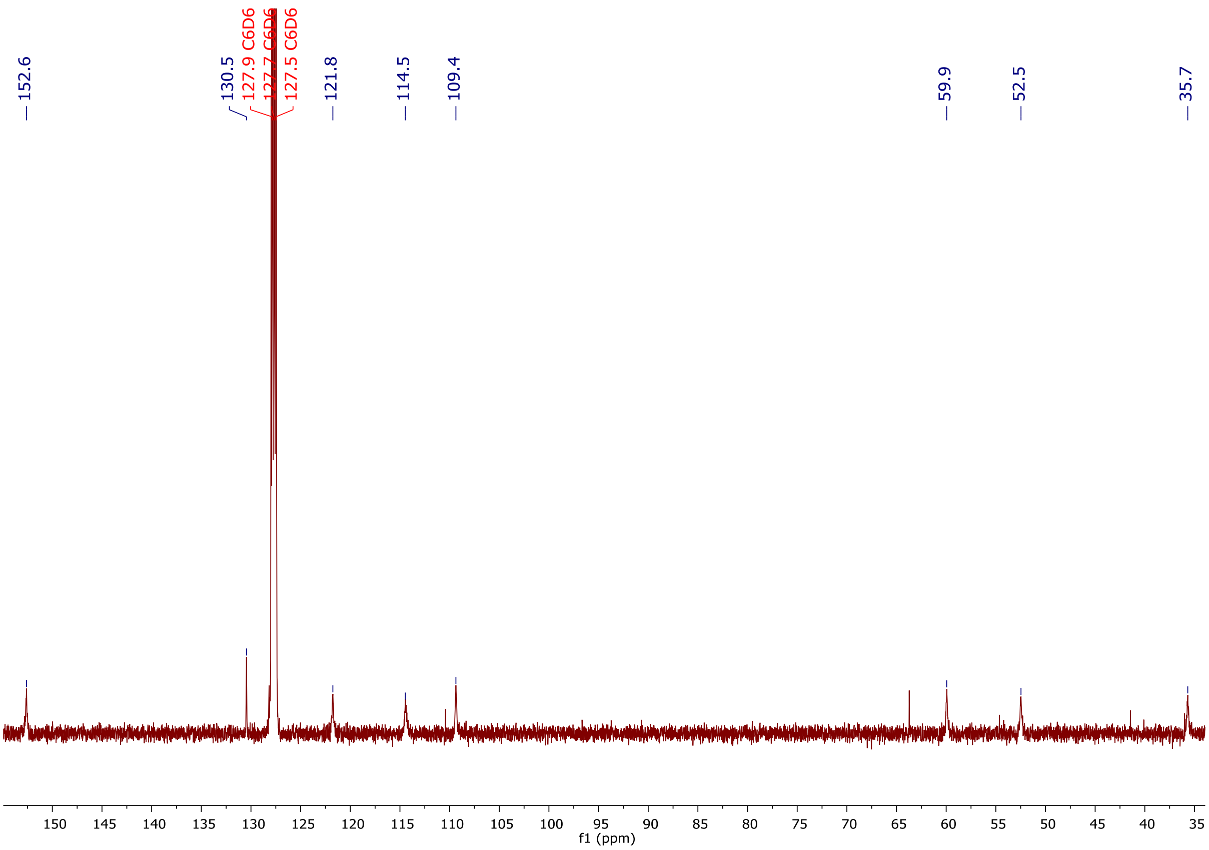

**Figure S6** $^{13}C$ {$^1H$} NMR (126 MHz, $C_6D_6$) spectrum for **3**H.

*2.2 Complex synthesis and characterisation*

Zn(**1**)$_2$

Ligand 1**H** (330 mg, 2 equiv, 2 mmol) was dried *in vacuo* for two hours and dissolved in anhydrous toluene (10 mL) in a Schlenk flask. Zn(Et)$_2$ (1 mL, 1 equiv, 1 mmol). The solution was stirred overnight before being concentrated. Pentane was added to induce crystallisation and a light brown product was collected, Zn(**1**)$_2$ (122 mg, 31%).

$^1H$ NMR (500 MHz, $C_6D_6$) δ 7.54 – 7.48 (m, 1H, **H**C=N), 7.18 (d, *J* = 1.6 Hz, 1H, **H**Ar), 6.87 (dd, *J* = 3.5, 1.0 Hz, 1H, **H**Ar), 6.63 (dd, *J* = 3.5, 1.7 Hz, 1H, **H**Ar), 3.05 (t, *J* = 6.2, 2H, C**H**$_2$), 2.08 (t, *J* = 6.1 Hz, 2H, C**H**$_2$), 1.76 (s, 6H, N(CH$_3$)$_2$).

$^{13}C$\{$^1H$\} NMR (126 MHz, $C_6D_6$) δ 158.7 (**C**=N), 136.4 (Ar**C**), 135.2 (Ar**C**), 116.5 (Ar**C**), 112.5 (Ar**C**), 59.2 (C**H**$_2$), 52.2 (C**H**$_2$), 44.4 (N(**C**H$_3$)$_2$).

Elemental analysis ($C_{18}H_{28}N_6Zn$) requires C, 54.89; H, 7.17; N, 21.34 %; found C, 54.59; H, 7.12; N, 20.72 %.

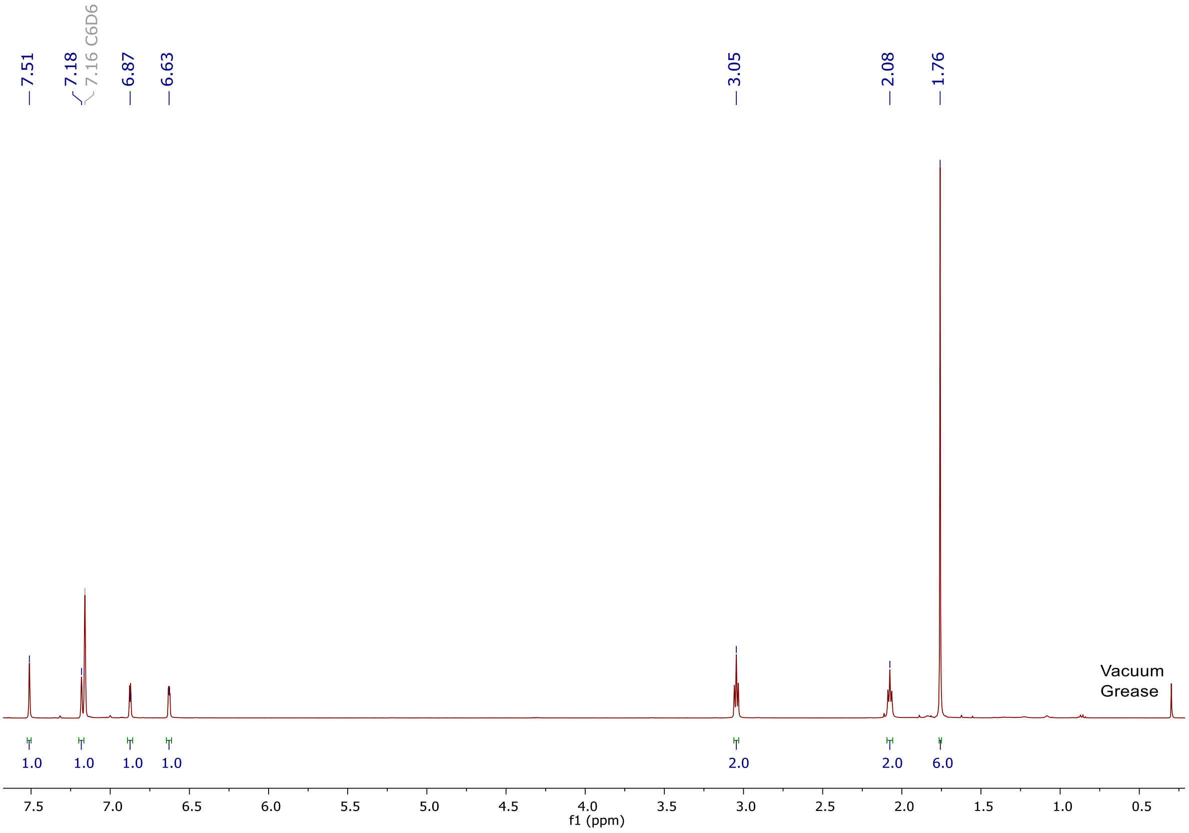


**Figure S7** $^{1}H$ NMR (500 MHz, $C_6D_6$) spectrum of Zn(**1**)$_2$.

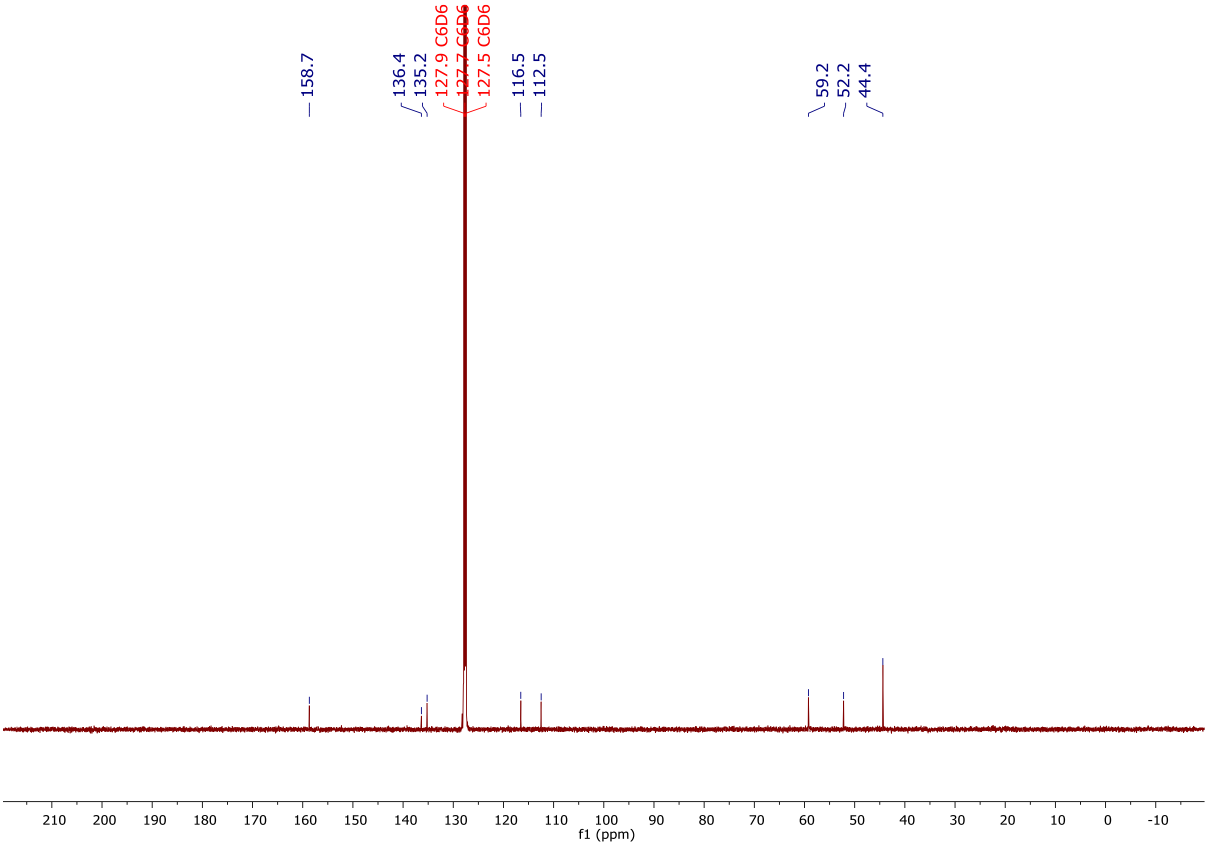


**Figure S8** $^{13}C\{^{1}H\}$ NMR (126 MHz, $C_6D_6$) spectrum of Zn(**1**)$_2$.

Zn(**2**)$_2$

Ligand 2**H** (360 mg, 2 equiv, 2 mmol) was dried *in vacuo* for two hours and dissolved in anhydrous toluene (10 mL) in a Schlenk flask. Zn(Et)$_2$ (1 mL, 1 equiv, 1 mmol). The solution was stirred overnight before being concentrated. Pentane was added to induce crystallisation and a light grey product was collected, Zn(**2**)$_2$ (140 mg, 33%).

$^1$H NMR (500 MHz, $C_6D_6$) δ 7.61 (s, 1H, **H**C=N)), 7.23 (s, 1H, **H**Ar), 6.89 (d, *J* = 3.5 Hz, 1H, **H**Ar), 6.68 (dd, *J* = 3.5, 1.7 Hz, 1H, **H**Ar), 3.25 (t, *J* = 6.8 Hz, 2H, C**H**$_2$), 1.96 (t, *J* = 6.4 Hz, 2H, C**H**$_2$), 1.89 (s, 6H, N(C**H**$_3$)$_2$), 1.40 (p, *J* = 6.6 Hz, 2H, C**H**$_2$).

$^{13}$C{$^1$H} NMR (126 MHz, $C_6D_6$) δ 159.0 (**C**=N), 136.3 (Ar**C**), 134.9 (Ar**C**), 116.5 (Ar**C**), 112.6 (Ar**C**), 57.2 (**C**H$_2$), 54.8 (**C**H$_2$), 45.1(N(**C**H$_3$)$_2$, 28.5 (**C**H$_2$).

Elemental analysis ($C_{20}H_{32}N_6Zn$) requires C, 56.94; H, 7.65; N, 19.92 %; found C, 54.85; H, 7.48; N, 18.64 %.

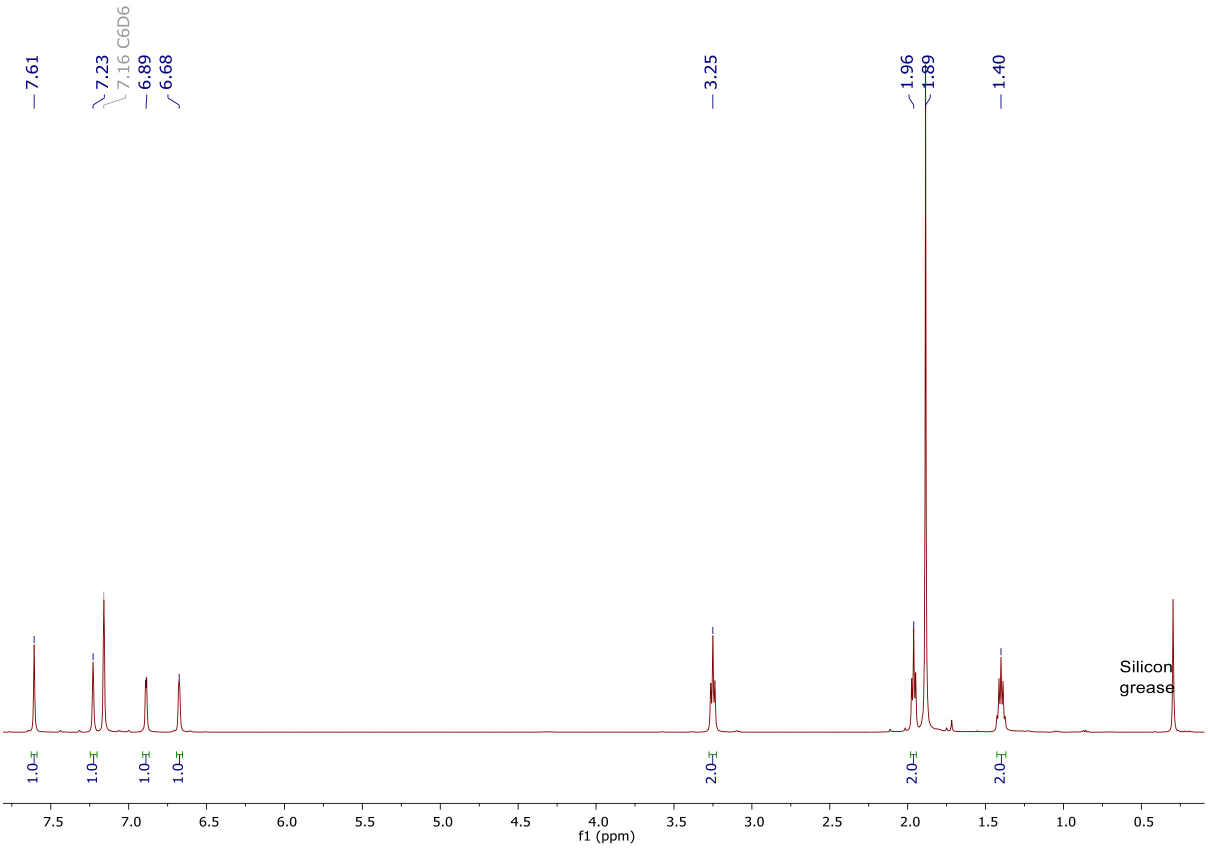


**Figure S9** $^1$H NMR (500 MHz, $C_6D_6$) spectrum of Zn(**2**)$_2$.

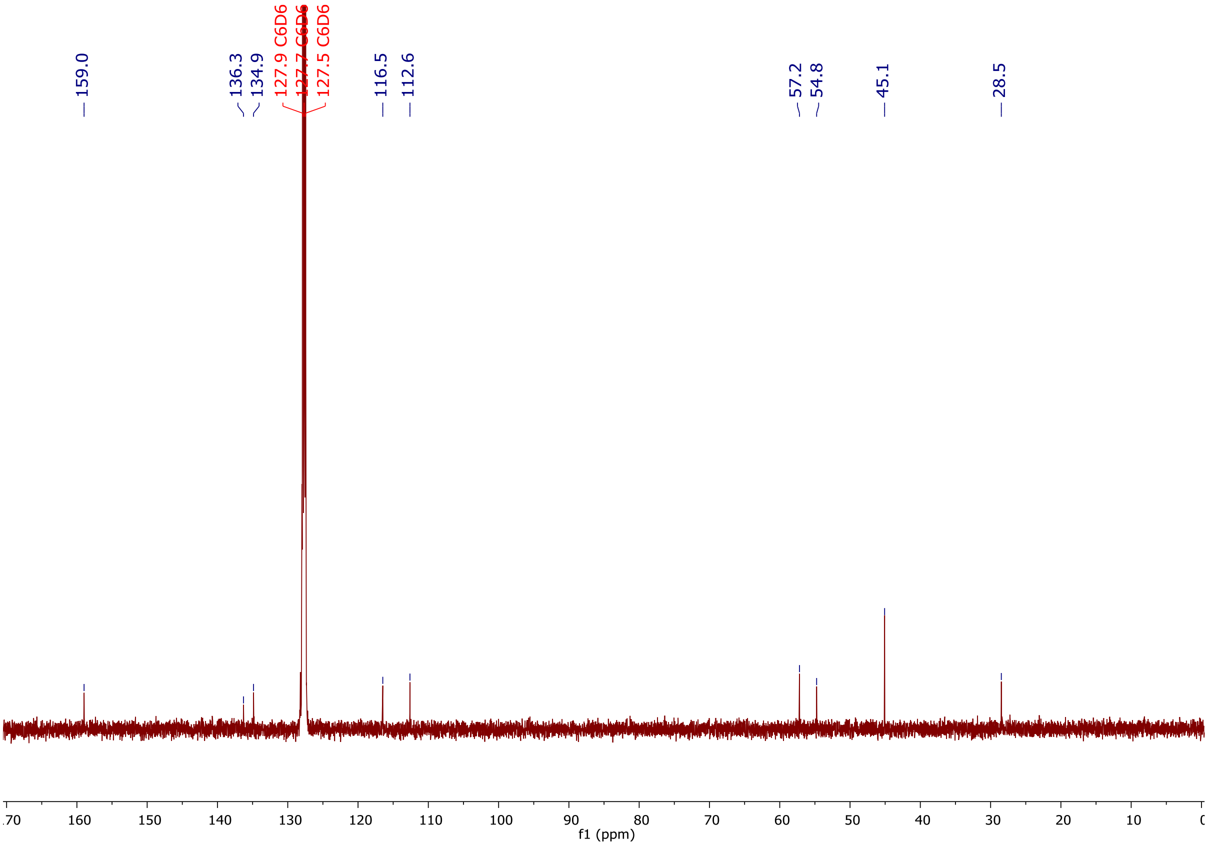


**Figure S10** $^{13}C\{^{1}H\}$ NMR (126 MHz, $C_6D_6$) spectrum of Zn(**2**)$_2$.

Zn(**3**)$_2$

Ligand 3**H** (304 mg, 2 equiv, 2 mmol) was dried *in vacuo* for two hours and dissolved in anhydrous toluene (10 mL) in a Schlenk flask. Zn(Et)$_2$ (1 mL, 1 equiv, 1 mmol). The solution was stirred overnight before being concentrated. Pentane was added to induce crystallisation and a light grey product was collected, Zn(**2**)$_2$ (295 mg, 81%).

$^{1}H$ NMR (500 MHz, $C_6D_6$) δ 7.51 (s, 1H, **H**C=N), 7.12 (d, $J$ = 3.3 Hz, 1H, **H**Ar), 6.87 (d, $J$ = 3.4 Hz, 1H, **H**Ar), 6.63 (d, $J$ = 3.1 Hz, 1H, **H**Ar), 3.00 (t, $J$ = 5.5 Hz, 2H, C**H**$_2$), 2.23 (d, $J$ = 6.0 Hz, 2H, C**H**$_2$), 1.84 (d, $J$ = 5.5 Hz, 3H, HNC**H**$_3$).

$^{13}C$ NMR (126 MHz, $C_6D_6$) δ 158.9 (**C**=N), 135.2 (Ar**C**), 125.5 (Ar**C**), 116.8 (Ar**C**), 112.8 (Ar**C**), 54.5 (**C**H$_2$), 51.9 (**C**H$_2$), 35.4 (**C**H$_3$).

Elemental analysis ($C_{20}H_{32}N_6Zn$) requires C, 52.54; H, 6.61; N, 22.98 %; found C, 57.09; H, 6.81; N, 18.97 %.

Increased carbon and hydrogen content represents toluene remaining in the sample, as evidenced by $^{1}H$ and $^{13}C\{^{1}H\}$ NMR spectroscopy.

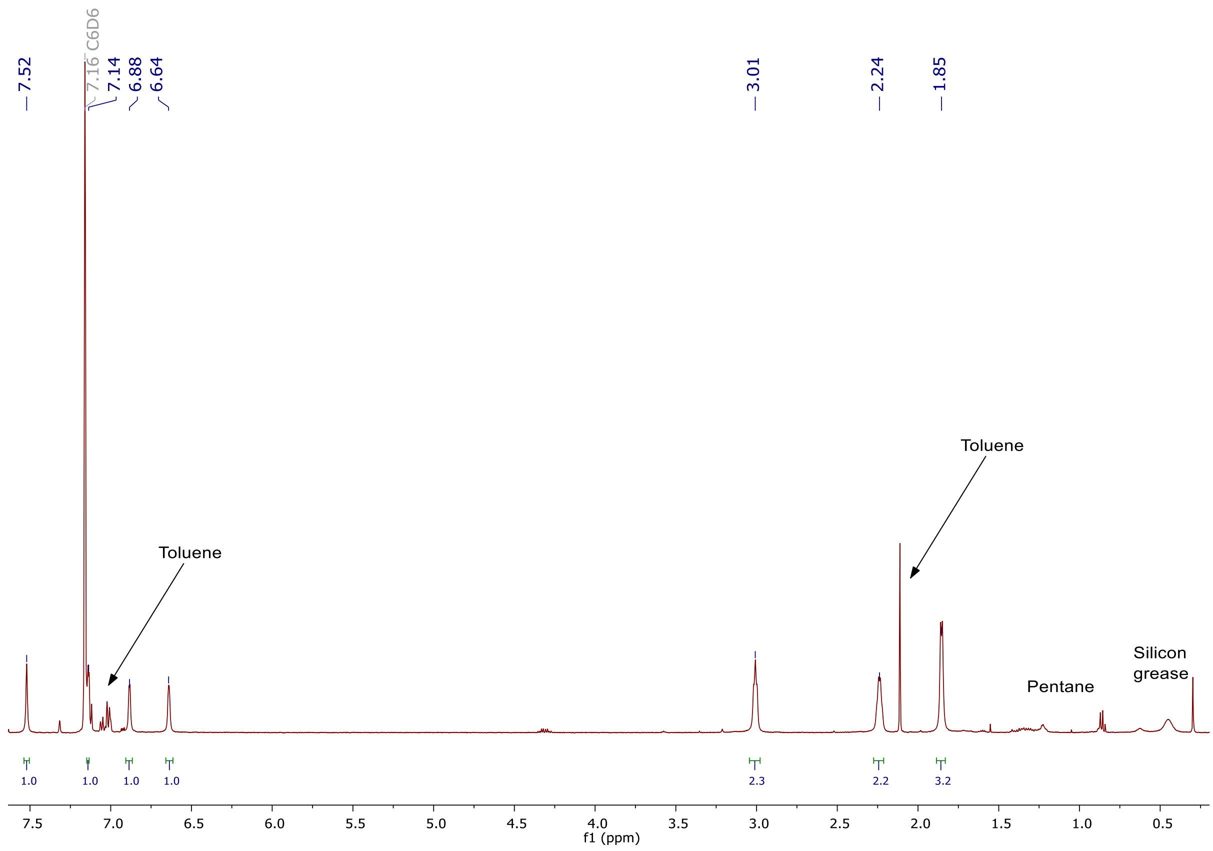


**Figure S11** $^{1}H$ NMR (500 MHz, 298 K, $C_6D_6$) spectrum of Zn(**3**)$_2$.

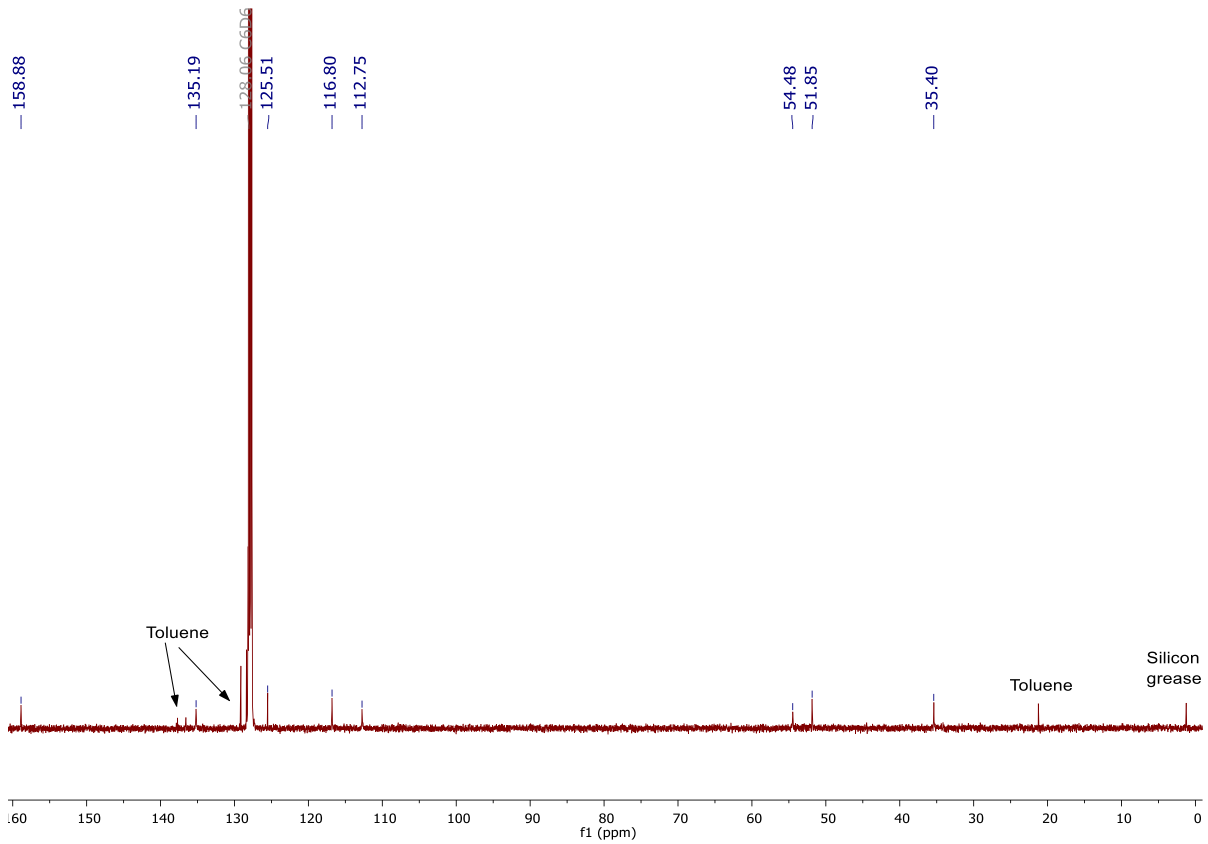


**Figure S12** $^{13}C\{^{1}H\}$ NMR (126 MHz, 298 K, $C_6D_6$) spectrum of Zn(**3**)$_2$.

*2.3 Crystallography Discussion*

The solid-state structure of Zn(**1**)$_2$ shows distorted tetrahedral geometry ($\tau_4'$ = 0.72) with coordination from both imine and pyrrole donors (**Figure S13**, **Table S1**). The pyrrole group is clearly deprotonated by the zinc precursor, further evidenced by a slightly shorter bond length compared to the zinc-imine bonds. The amine groups are not formally shown as bonded to the zinc centre; however, the measured distances are approximately the sum of the van der Waals radii, suggesting there could be a weak interaction.[1] The position of the amine groups suggest that they are influencing the geometry of the complex towards octahedral, accounting for the deviation from ideal tetrahedral angles. The largest coordination angle is from a pyrrole to an imine group and significantly exceeds the ideal tetrahedral angle (N(2) – Zn – N(4) = 129.48(8)°).

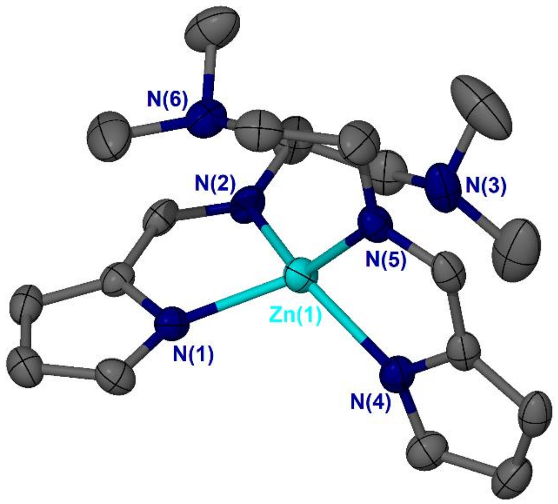


**Figure S13** Solid-state structure of Zn(**1**)$_2$. Ellipsoids are shown at the 50% probability level. Hydrogen atoms have been removed for clarity.

The solid-state structure of Zn(**2**)$_2$ (**Figure S14**) shows a 5-coordinate geometry with both pyrrole and imine groups bonding to the zinc centre alongside one pendant amine. This conformation is very similar to that seen with a dibromo zinc half salan complex, which constitutes one of the most effective catalysts in the literature for the degradation of multiple polymers.[2] The largest coordination angle lies between imine, N(2), and pyrrole, (N(4), and is very close to the ideal angle of 180 °C for apical donors. The largest equatorial angle is 132.50(10)° and this appears to correspond to space created by the free pendant amine. This could explain the deviation from perfect trigonal bipyramidal geometry ($\tau_5$ = 0.75). The coordinated amine is much closer to zinc than with Zn(**1**)$_2$ (Zn – N(3) = 2.172 Å) however the free amine is considerably farther away due to crowding of the coordination sphere (Zn – N(6) = 5.952 Å).

**Table S1.** Selected bond lengths (Å) and angles (°) for Zn(**1**)$_2$, Zn(**2**)$_2$ and Zn(**3**)$_2$CO$_2$.

| | Donor type | Zn(**1**)$_2$ | Zn(**2**)$_2$ | Zn(**3**)$_2$(CO$_2$) |
|---|---|---|---|---|
| $\tau_4$'[a] | - | 0.72 | - | - |
| $\tau_5$[a] | - | - | 0.75 | 0.84 |
| Zn – N(1) | Pyrrole | 2.0201(19) | 2.009(2) | 1.9929(12) |
| Zn – N(4) | | 2.024(2) | 2.104(3) | - |
| Zn – N(2) | Imine | 2.0481(19) | 2.193(2) | 2.1769(13) |
| Zn – N(5) | | 2.0546(19) | 2.090(3) | 2.2593(12) |
| Zn – N(3) | Amine | 2.954[b] | 2.172(3) | - |
| Zn – N(6) | | 3.055[b] | 5.952[b] | 2.593(12) |
| Zn – O(1) | Carbamate | - | - | 1.9879(10) |
| N(2) – Zn – N(4) | Imine – pyrrole | 129.48(8) | 177.32(9) | - |
| N(2) – Zn – N(5) | Imine – imine | 127.73(8) | 100.52(10) | 171.36(5) |
| N(1) – Zn – N(5) | Pyrrole – imine | 124.78(8) | 132.50(10) | 94.04(5) |

[a] Calculated from the two largest coordination angles. [b] Measured using Mercury ™ software.

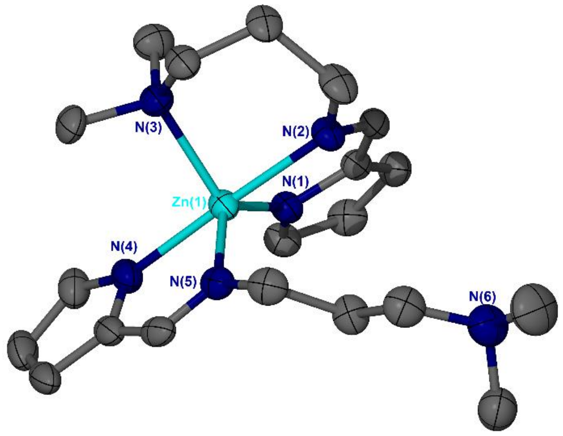


**Figure S14.** Solid-state structure of Zn(**2**)$_2$. Ellipsoids are shown at the 50% probability level. The propyl backbone (N2-N3) and methyl groups on N3 are disordered over 2 positions in a 60:40 ratio – the major form is shown. There is a molecule of MeOH in the unit cell which has been removed for clarity. Hydrogen atoms have also been removed for clarity.

Efforts to produce crystals suitable for single crystal XRD from Zn(**3**)$_2$ were unsuccessful under air-sensitive conditions. However, crystals were obtained in a vial from bench DCM and methanol (**Figure S15**). Interestingly, this structure contains a molecule of inserted $CO_2$ between Zn and one secondary amine group, resulting in a coordinated carbamate donor. This complex has been previously reported by Sousa *et al*. who used electrochemical methods to obtain the same product, but did not apply it as a catalyst.[3] The result is a *pseudo* trigonal bipyramidal structure ($\tau_5$ = 0.84) with imine groups in the axial positions and amine, pyrrole and carbamate adopting equatorial sites. One pyrrole group remains protonated and does not coordinate to the zinc in this case. It is unclear whether this is the case prior to $CO_2$ insertion or if the resulting carbamate displaces the pyrrole of the other ligand. The formation of the carbamate explains the insolubility of the complex after exposure to air.

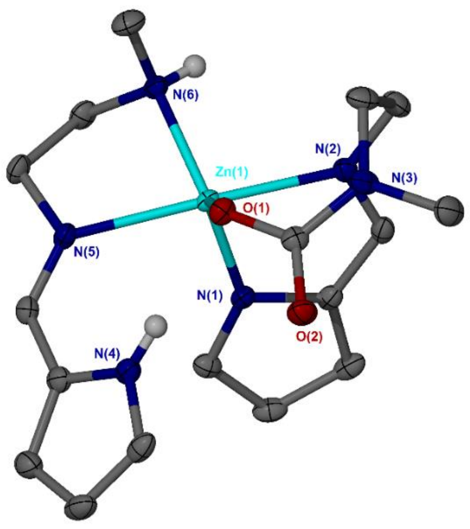


**Figure S15** Solid-state structure of Zn(**3**)$_2$($CO_2$). Ellipsoids are shown at the 50% probability level. Hydrogen atoms have been removed for clarity.

### *2.4 Air sensitiviy tests*

The stability of Zn(**1**–**3**)$_2$ in air was investigated by running $^1$H NMR samples under air sensitive conditions, then leaving the solvent to evaporate in a vial, before running the sample again in bench $C_6D_6$ (**Figure S16**–**18**)**.** For Zn(**1**)$_2$ and Zn(**2**)$_2$, the position and integrations of the complex peaks was unchanged, however a broadening was observed and splitting definition was lost. This is likely a result of hydrogen bonding with atmospheric $H_2O$. For Zn(**3**)$_2$, the resulting solid was insoluble in $C_6D_6$, suggesting a more significant structural change.

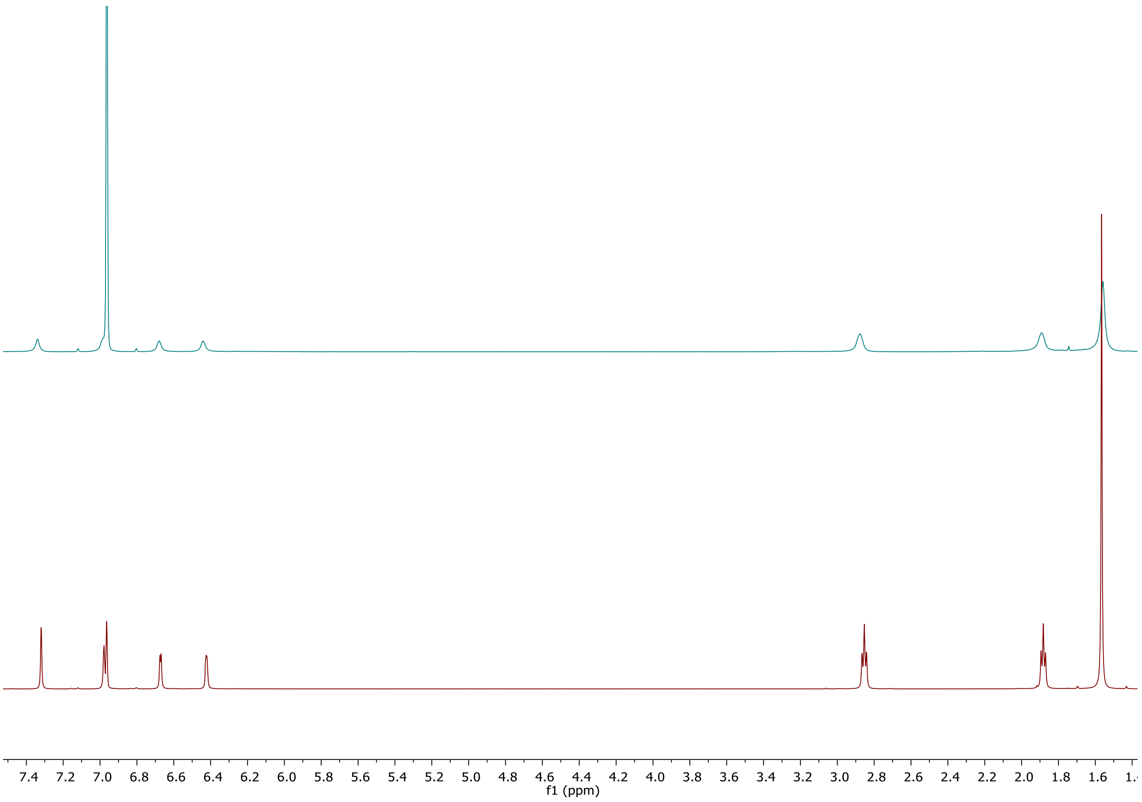


**Figure S16** $^1$H NMR (500 MHz, $C_6D_6$) spectrum of Zn(**1**)$_2$ under air sensitive conditions (bottom) and after exposure to air (top).

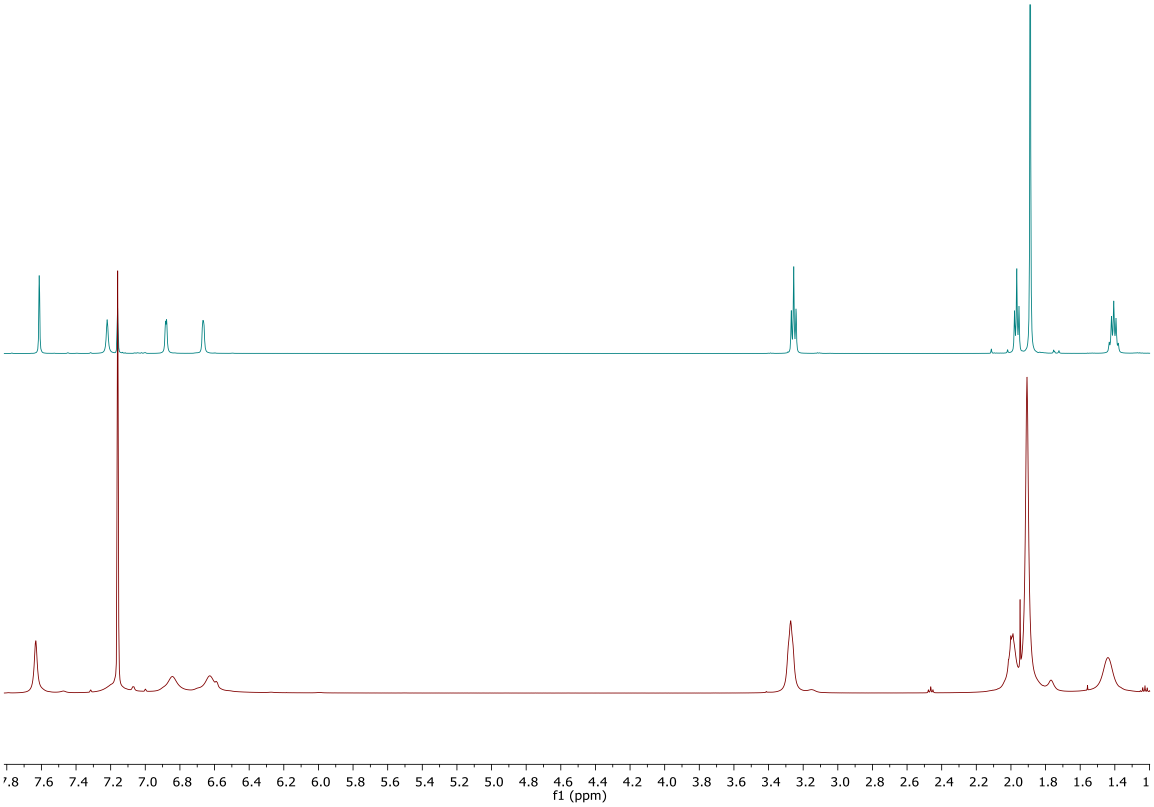


**Figure S17** $^{1}H$ NMR (500 MHz, $C_6D_6$) spectrum of Zn(**2**)$_2$ under air sensitive conditions (bottom) and after exposure to air (top).

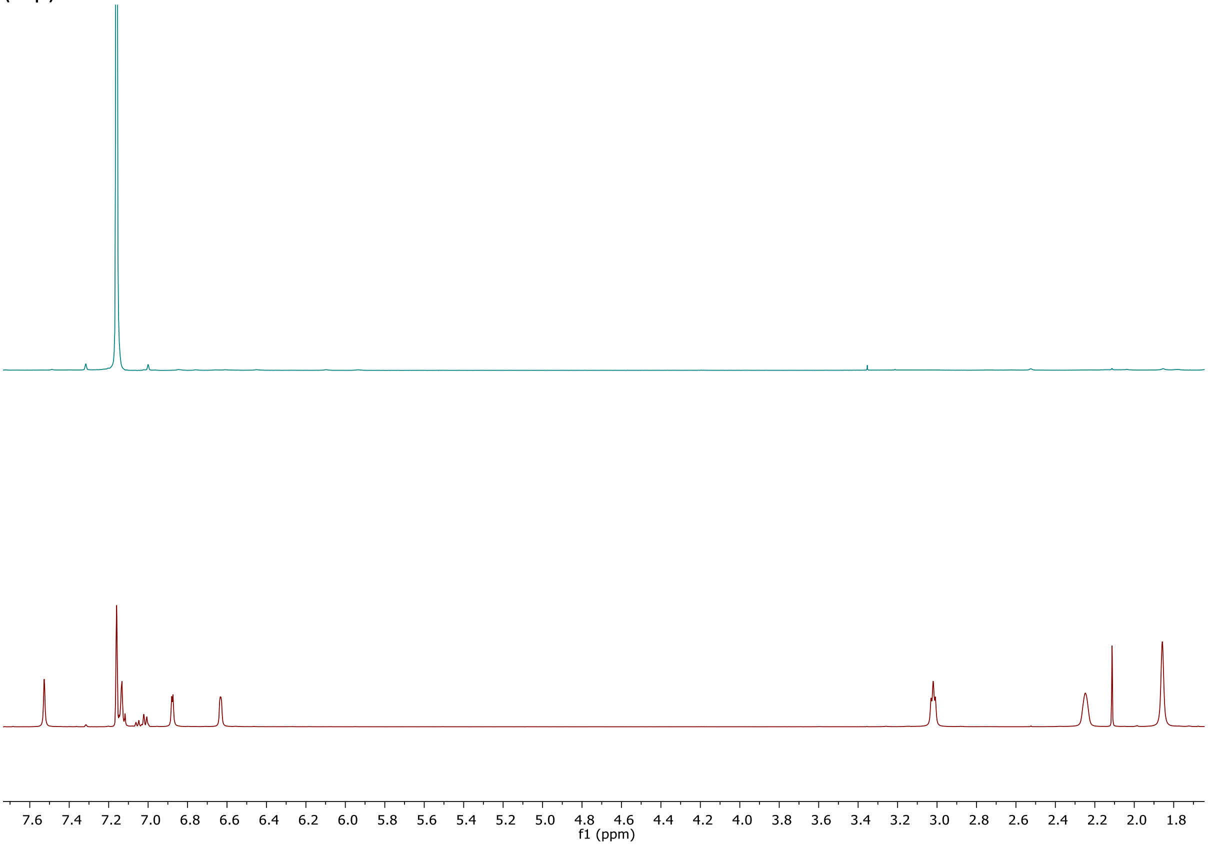


**Figure S18** $^{1}H$ NMR (500 MHz, $C_6D_6$) spectrum of Zn(**3**)$_2$ under air sensitive conditions (bottom) and after exposure to air (top).

# 3. Lactide Polymerisation

## *3.1 Solvent-free lactide ROP*

**Table S2** Solvent free ROP of *L*-lactide using Zn(**1**–**3**)$_2$ at 130 °C.

| Entry | Init. | [*L*-LA]/[Zn]/[BnOH] | Time [min] | Conv. [%][d] | $M_n$ [gmol$^{-1}$][e] | $M_{n\ theo.}$ [gmol$^{-1}$][f] | Đ[e] | TOF [h$^{-1}$][g] |
|---|---|---|---|---|---|---|---|---|
| 1 | Zn(**1**)$_2$ | 300 : 1 : 1[a] | 13 | 80 | 14150 | 34700 | 1.09 | 1100 |
| 2 | Zn(**2**)$_2$ | 300 : 1 : 1[a] | 1 | 94 | 24850 | 40750 | 1.18 | 16900 |
| 3 | Zn(**3**)$_2$ | 300 : 1 : 1[a] | 2 | 85 | 22250 | 36850 | 1.13 | 7650 |
| 4 | Zn(**1**)$_2$ | 3000 : 1 : 10[b] | 60 | 17 | 4450 | 7450 | 1.06 | 500 |
| 5 | Zn(**2**)$_2$ | 3000 : 1 : 10[b] | 2 | 71 | 29350 | 30800 | 1.05 | 63900 |
| 6 | Zn(**3**)$_2$ | 3000 : 1 : 10[b] | 6 | 63 | 31450 | 27350 | 1.07 | 18900 |
| 7 | Zn(**2**)$_2$ | 10000 : 1 : 30[c] | 60 | 17 | 7050 | 8300 | 1.10 | 1700 |
| 8 | Zn(**3**)$_2$ | 10000 : 1 : 30[c] | 60 | 44 | 28300 | 21250 | 1.04 | 4400 |

[a] Conditions: recrystallised *L*-lactide (1 g), [LA]/[Zn]/[BnOH] = 300 : 1 : 1, solvent free. [b] Conditions: recrystallised *L*-lactide (2.5 g), [LA]/[Zn]/[BnOH] = 3000 : 1 : 10, solvent free. [c] Conditions: recrystallised *L*-lactide (5 g), [LA]/[Zn]/[BnOH] = 10000 : 1 : 30, solvent free. [d] Calculated using $^1$H NMR spectroscopy. [e] Determined from GPC (in tetrahydrofuran) referenced against polystyrene standards. [f] Theoretical molecular weight calculated from conversion: {(conversion × 3 × $M_n$ [LA]) + $M_n$ [BnOH]}. [g] TOF = $[LA]_t/([Zn] \times t)$.

## *3.2 Selected lactide conversion examples*

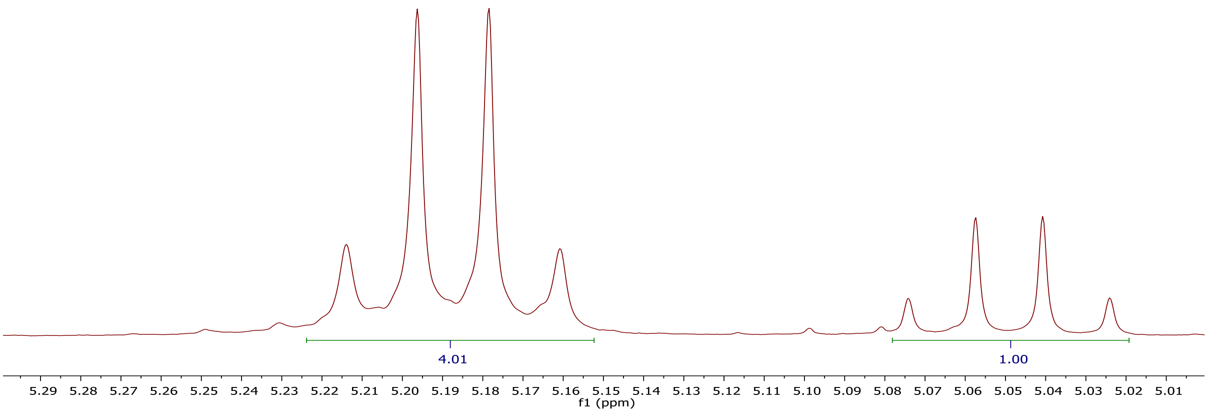


**Figure S19** $^1$H NMR (400 MHz, $CDCl_3$) spectrum of PLA from Zn(**1**)$_2$. [LA]/[Zn]/[BnOH] = 300 : 1 : 1. Conversion = 80%.

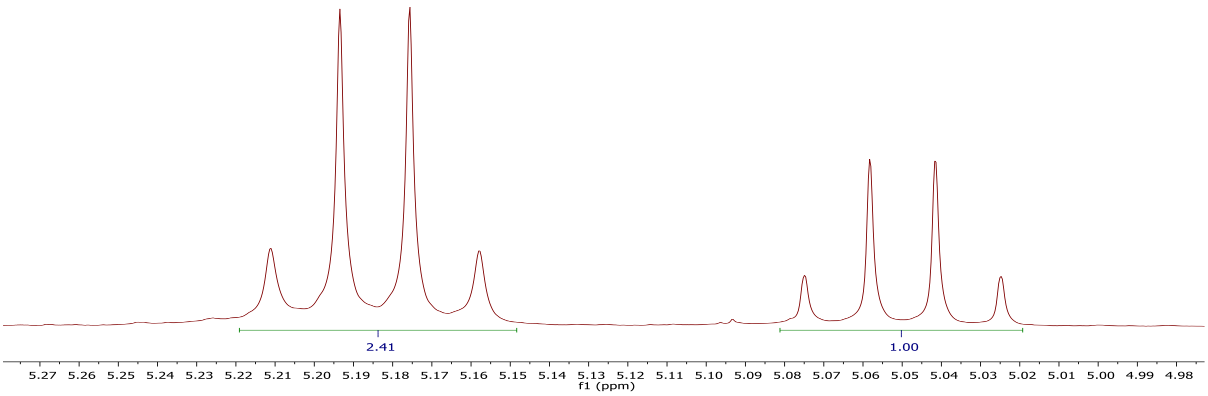


**Figure S20** $^1$H NMR (400 MHz, $CDCl_3$) spectrum of PLA from Zn(**2**)$_2$. [LA]/[Zn]/[BnOH] = 3000 : 1 : 10. Conversion = 71%.

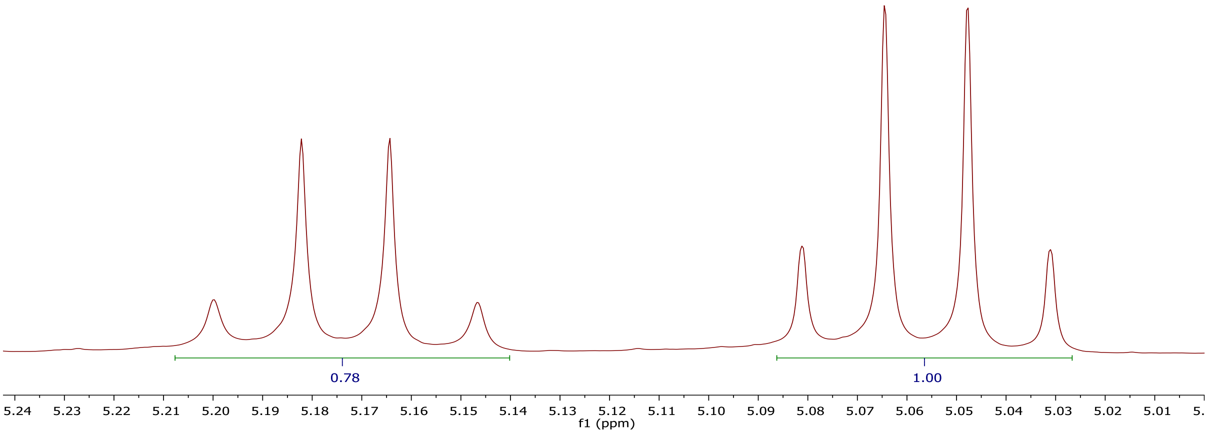


**Figure S21** $^1H$ NMR (400 MHz, $CDCl_3$) spectrum of PLA from Zn(**3**)$_2$. [LA]/[Zn]/[BnOH] = 10000 : 1 : 30. Conversion = 44%.

### *3.3 Selected GPC traces*

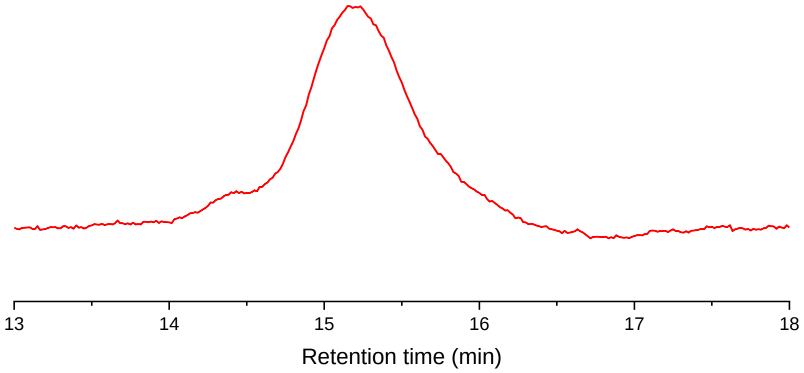


**Figure S22** GPC trace of PLA from Zn(**1**)$_2$ (130 °C, 60 mins). ([LA]/[I]/[BnOH] = 3000 : 1 : 10) in the melt. $M_{n,\,GPC}$ = 4450 gmol$^{-1}$, Đ = 1.06, $M_{n,\,theo.}$ = 7450 gmol$^{-1}$.

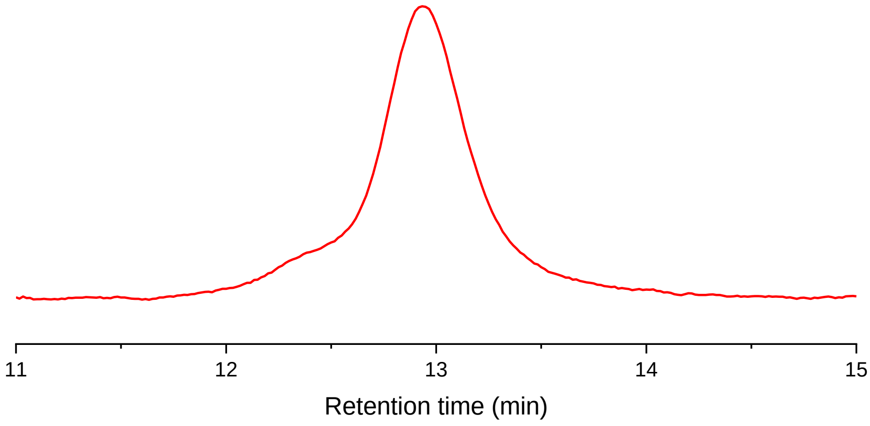


**Figure S23** GPC trace of PLA from Zn(**2**)$_2$ (130 °C, 1 min). ([LA]/[I]/[BnOH] = 300 : 1 : 1) in the melt. $M_{n,\,GPC}$ = 24850 gmol$^{-1}$, Đ = 1.18, $M_{n,\,theo.}$ = 40750 gmol$^{-1}$.

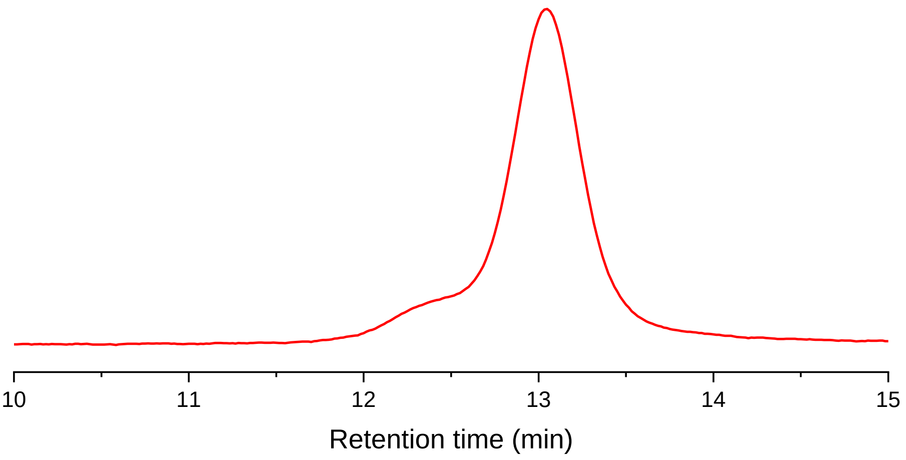


**Figure S24** GPC trace of PLA from Zn(**3**)$_2$ (130 °C, 6 mins). ([LA]/[I]/[BnOH] = 3000 : 1 : 10) in the melt. $M_{n,\,GPC}$ = 31450 gmol$^{-1}$, Đ = 1.07, $M_{n,\,theo.}$ = 27350 gmol$^{-1}$.

## 4. Solution alcoholysis of PLA and BPA-PC

### *4.1 PLA methanolysis – conversion, selectivity and yield*

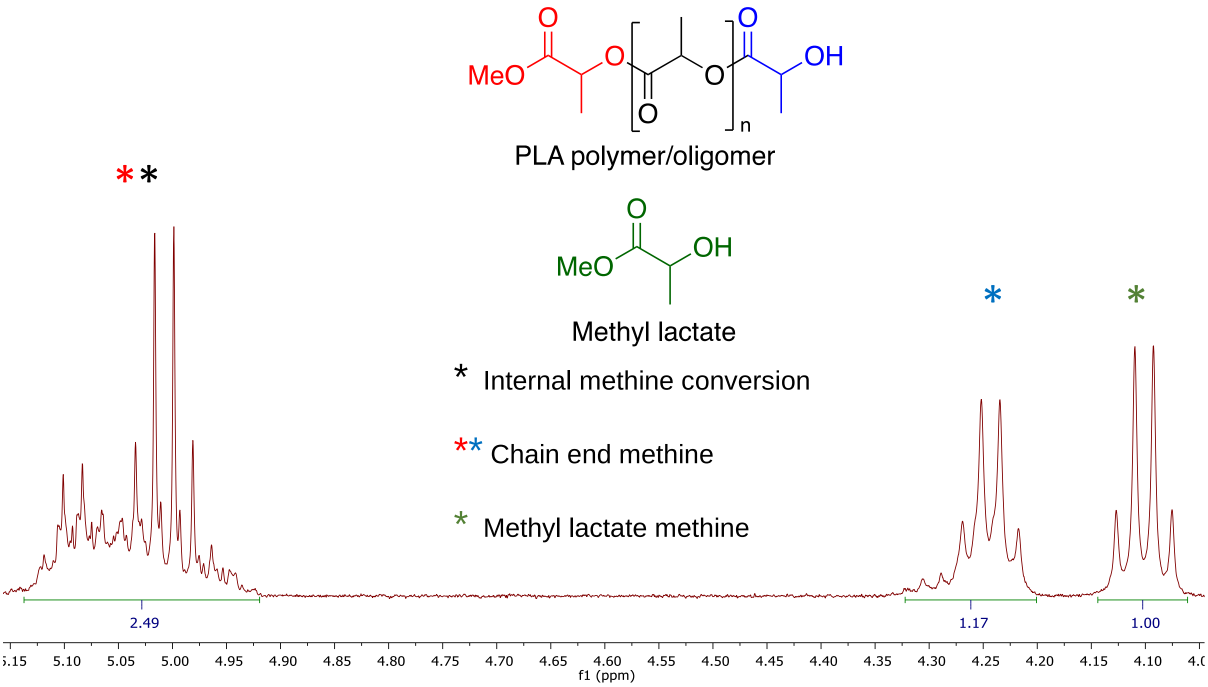


**Figure S25** $^1$H NMR ($CDCl_3$, 500MHz, δ = 5.2 – 4.0 ppm) spectrum of PLA degradation into MeLA using Zn(**3**)$_2$.

$$[Me-LA] = \frac{1}{(1+1.17+2.49)} x\ 100 = 21.5\%$$

$$[Int] = \frac{(2.49-1.17)}{(1+1.17+2.49)} x\ 100 = 28.3\%$$

$$[CE] = \frac{1.17}{(1+1.17+2.49)} x\ 100 = 25.1\%$$

$$[CE] = \frac{1.17}{(1+1.17+2.49)} x\ 100 = 25.1\%$$

Utilising the relative quantities shown in **Figure S25**, the following key parameters can be calculated from equations (1–3). The conversion of internal methine ($X_{int}$) represents the overall conversion of PLA to oligomers and methyl lactate. The methyl lactate yield ($Y_{MeLA}$) and selectivity ($S_{MeLA}$) are used to monitor the completion of the reaction to methyl lactate.

$$X_{int} = 1 - \frac{[int]}{[int]_0} \quad \textbf{(1)}$$

$$S_{Me-LA} = \frac{[Me-LA]}{[int]_0-[int]} \quad \textbf{(2)}$$

$$Y_{Me-LA} = X_{int}S_{Me-LA} \quad \textbf{(3)}$$

*4.2 PLA methanolysis – pseudo*-First Order Kinetics and *Conversion vs. Time plot*

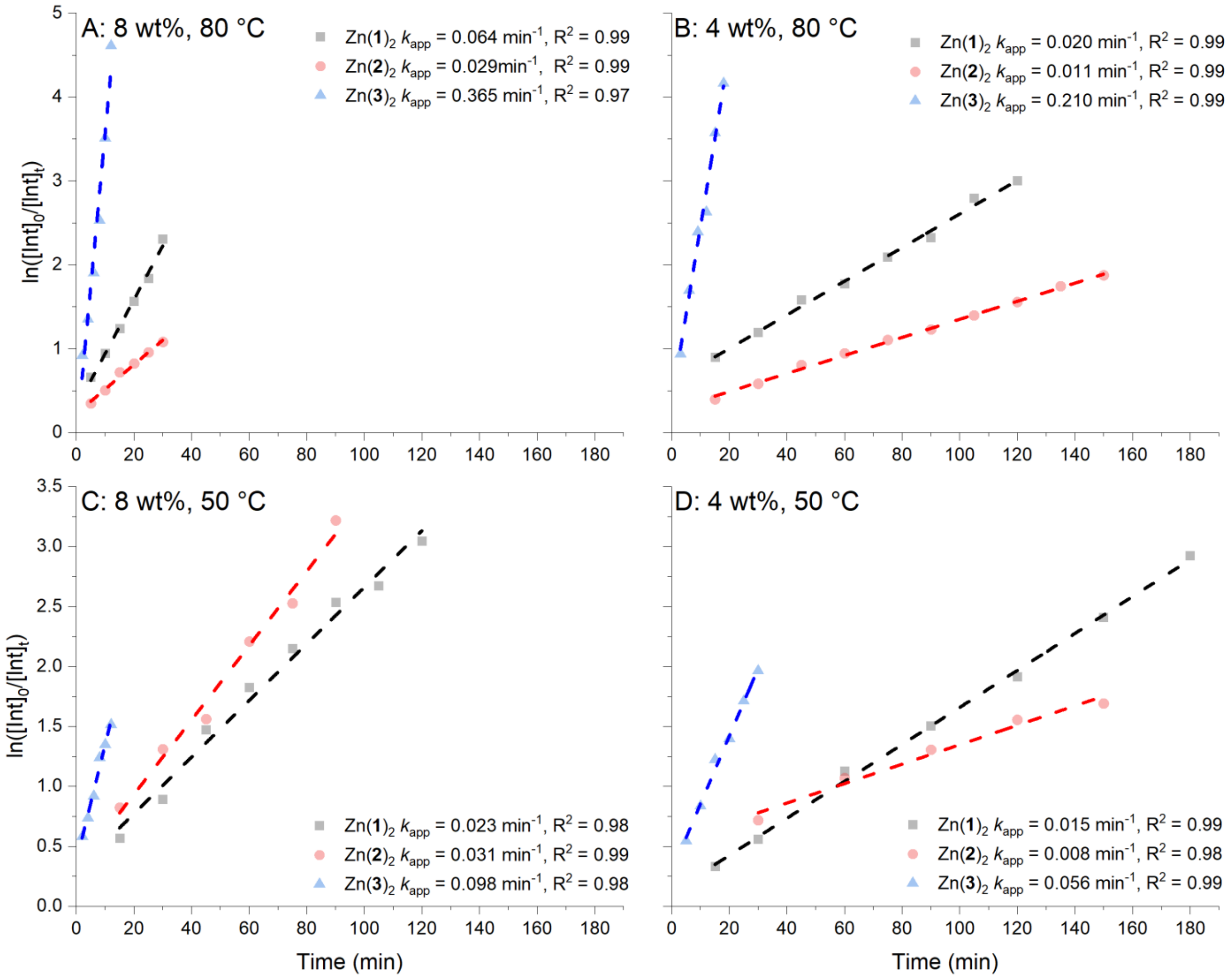


**Figure S26.** Semi logarithmic plots for PLA degradation with Zn(**1**–**3**)$_2$ at various conditions.

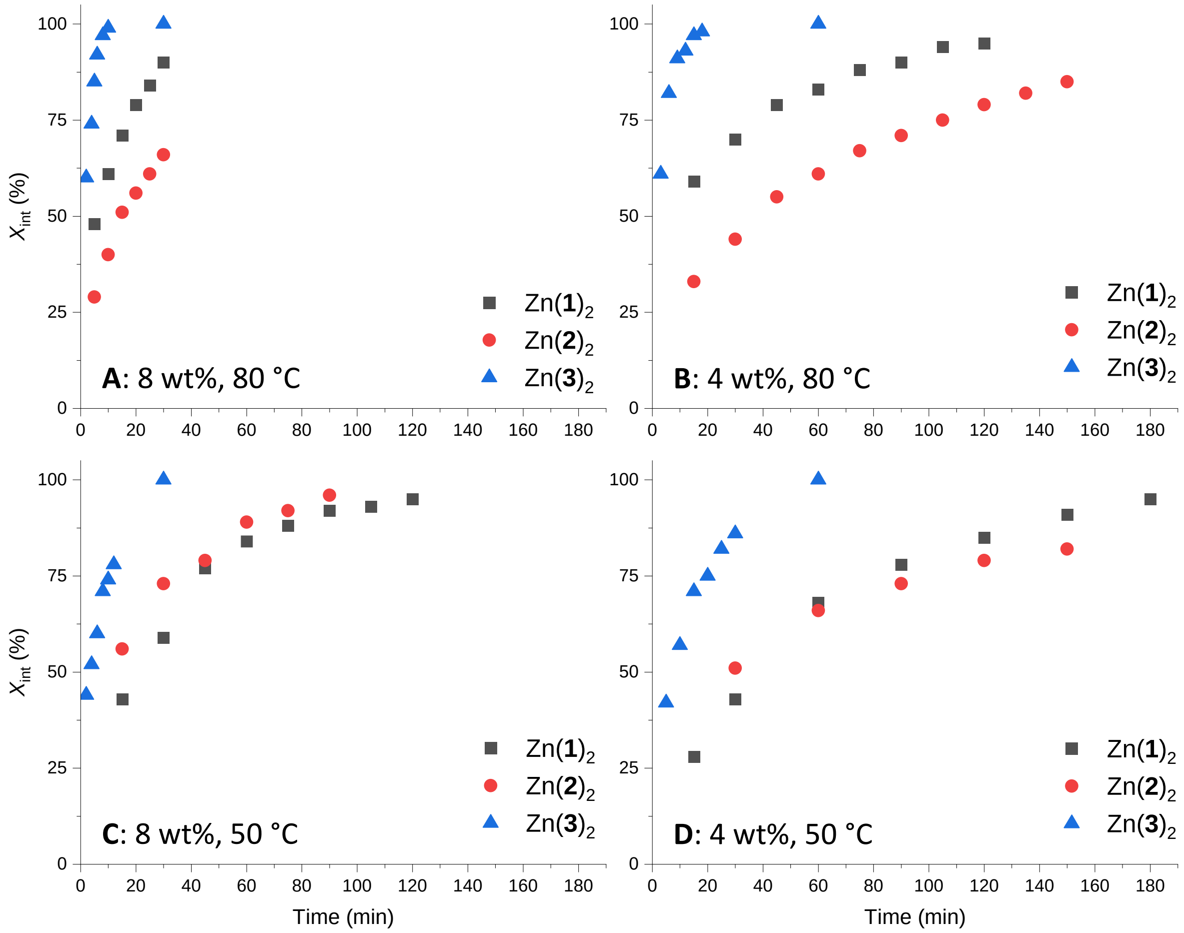


**Figure S27** Conversion *vs*. time plots for Zn(**1**–**3**)$_2$ at various conditions.

## *4.3 Ethanolysis and butanolysis of PLA*

**Table S3** Degradation of PLLA cup to ethyl lactate and butyl lactate using Zn(**1**)$_2$ and Zn(**3**)$_2$.[a]

| Cat. | Alcohol | Time [h] | Loading [wt%] | $Y_{Et/Bu\text{-}LA}$ [%][b] | $S_{Et/Bu\text{-}LA}$ [%][b] | $X_{int}$ [%][b] | $k_{app}$ [min$^{-1}$] |
|---|---|---|---|---|---|---|---|
| Zn(**1**)$_2$ | EtOH | 1 | 8 | 30 | 40 | 74 | - |
| Zn(**3**)$_2$ | EtOH | 1 | 8 | 70 | 70 | 100 | 0.127 |
| Zn(**1**)$_2$ | n-BuOH | 2 | 8 | 12 | 25 | 46 | - |
| Zn(**3**)$_2$ | n-BuOH | 1 | 8 | 42 | 49 | 87 | 0.073 |

[a] Reaction conditions: 0.25 g of PLLA cup ($M_n$ = 45,510 g mol$^{-1}$), $V_{THF}$:$V_{ALC}$ = 4:1, $n_{ALC}$:$n_{ester}$ = 7:1, 8 wt% cat. loading (10 mg, 1.4 – 1.6 mol% relative to ester linkages). [b] $^1$H NMR (400MHz, 298 K, $CDCl_3$) spectroscopy used to calculate $Y_{Et/Bu\text{-}LA}$, $S_{Et/Bu\text{-}LA}$ and $X_{Int}$. $^b$ Alcohol: ethanol, $^c$ Alcohol: *n*-butanol.

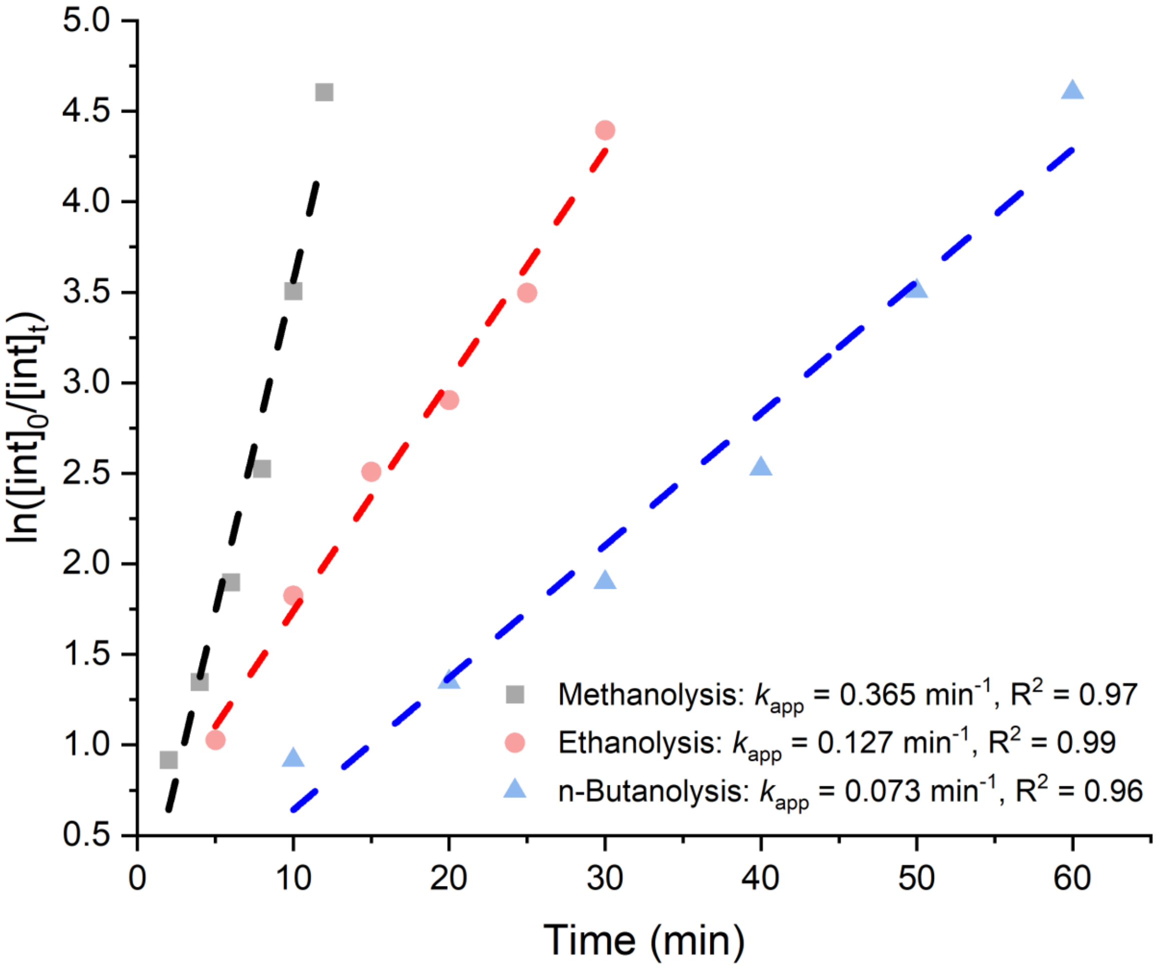


**Figure S28** Semi logarithmic plots for PLA alcoholysis with methanol, ethanol and n-butanol.

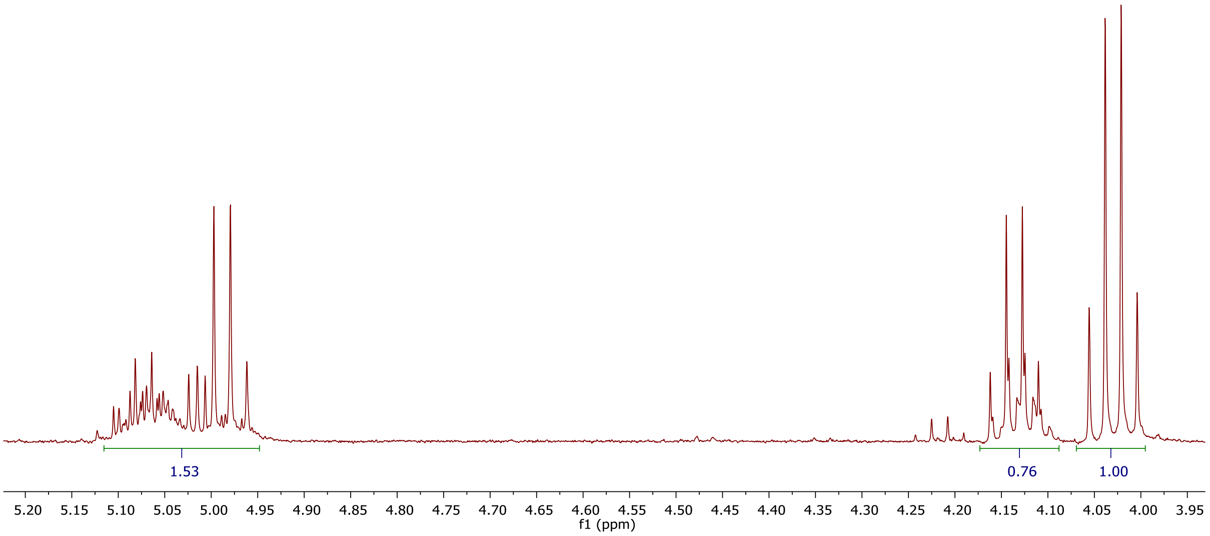


**Figure S29** $^1H$ NMR ($CDCl_3$, 500MHz, δ = 5.2 – 3.9 ppm) spectrum of PLA degradation into Et-LA using Zn(**1**)$_2$. (8 wt%, 80 ˚C, 1 hr).

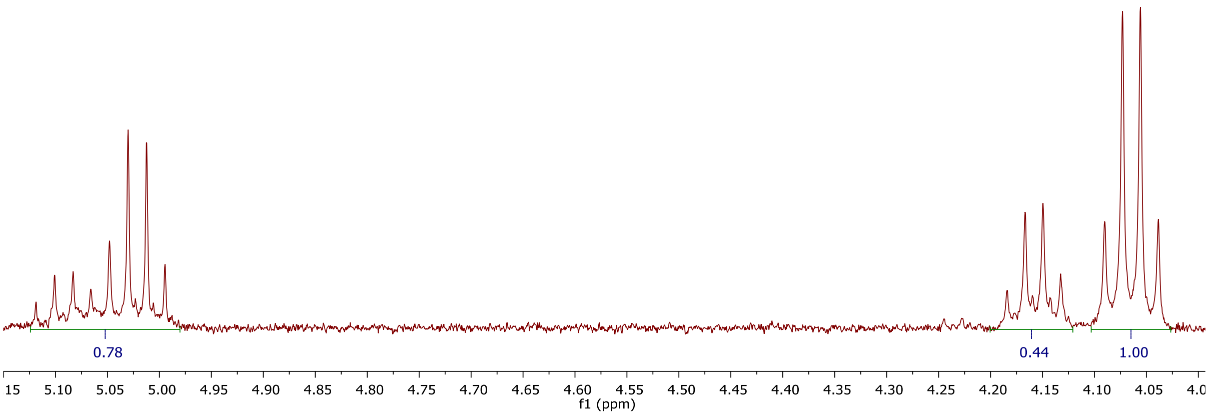


**Figure S30** $^1$H NMR ($CDCl_3$, 500MHz, δ = 5.2 – 4 ppm) spectrum of PLA degradation into nBu-LA using Zn(**3**)$_4$. (8 wt%, 80˚C, 1 hr).

### *4.4 Methanolysis of BPA-PC*

**Table S4** Degradation of BPA-PC to BPA *via* methanolysis using Zn(**1–3**)$_2$.[a]

| Cat. | Time [h] | Loading [wt%] | $S_{BPA}$ [%][b] | $S_{DC\text{-}BPA + MC\text{-}BPA}$ [%][b] | $Y_{BPA}$ (Isolated) [%][c] |
|---|---|---|---|---|---|
| Zn(**1**)$_2$ | 1 | 4 | 68 | 32 | 53 |
| Zn(**1**)$_2$ | 1 | 8 | 95 | 5 | 87 |
| Zn(**2**)$_2$ | 1 | 4 | 85 | 15 | 76 |
| Zn(**2**)$_2$ | 1 | 8 | 92 | 8 | 83 |
| Zn(**3**)$_2$ | 1 | 4 | 96 | 4 | 91 |
| Zn(**3**)$_2$ | 1 | 8 | 97 | 3 | 93 |

[a] Reaction conditions: 0.25 g BPA-PC pellets ($M_n$ = 45,000 gmol$^{-1}$), 17.5 equivalents of MeOH (relative to carbonate linkages), 4 – 8 wt% catalyst loading (10 – 20 mg, 2.4 – 5.2 mol% based on carbonate linkages. [b] Selectivities based on $^1$H NMR spectroscopy. [c] BPA was recrystallised from water and dried to constant weight.

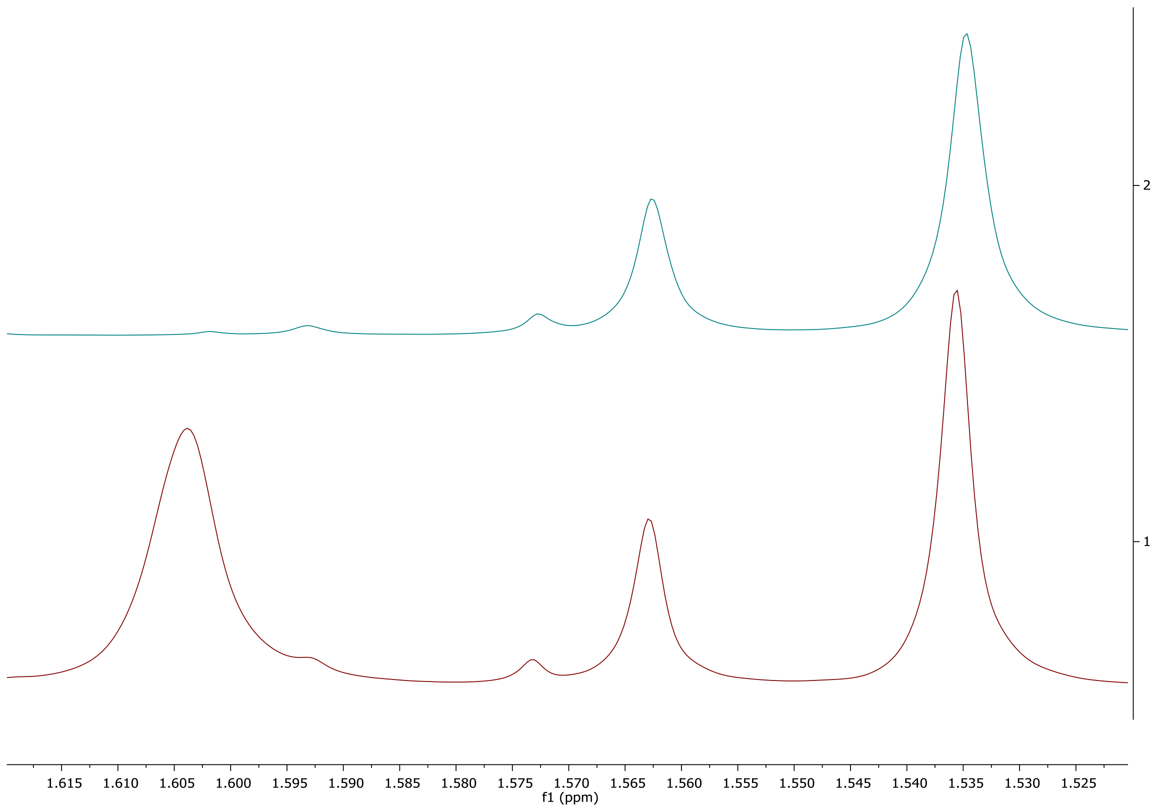


**Figure S31** $^1$H NMR ($CDCl_3$. 500 MHz) spectrum of BPA-PC methanolysis (**Table S3**, entry 1). Reaction aliquot (top) and reaction aliquot spiked with BPA-PC in $CDCl_3$ (bottom).

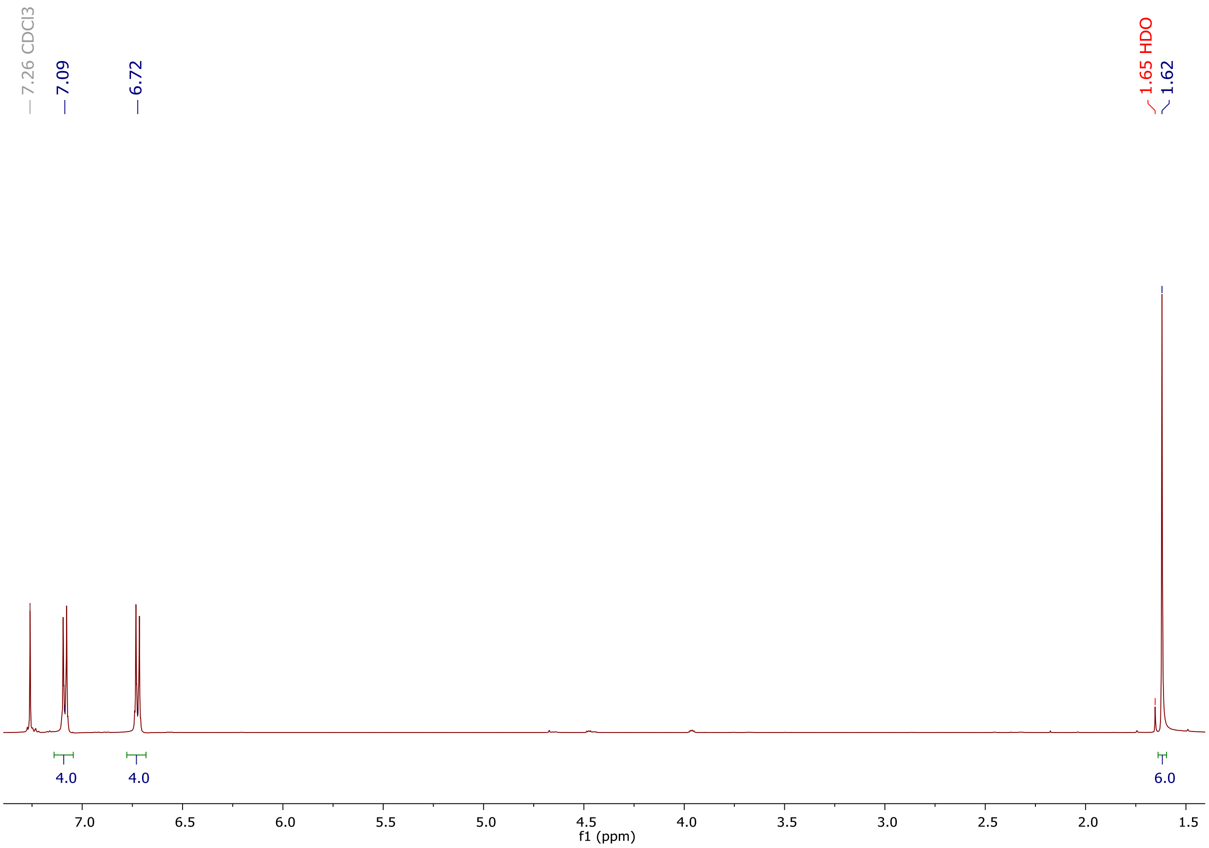


**Figure S32** Representative $^{1}$H NMR ($CDCl_3$, 500 MHz) spectrum of recrystallised BPA from BPA-PC methanolysis (**Table S3**, entry 6).

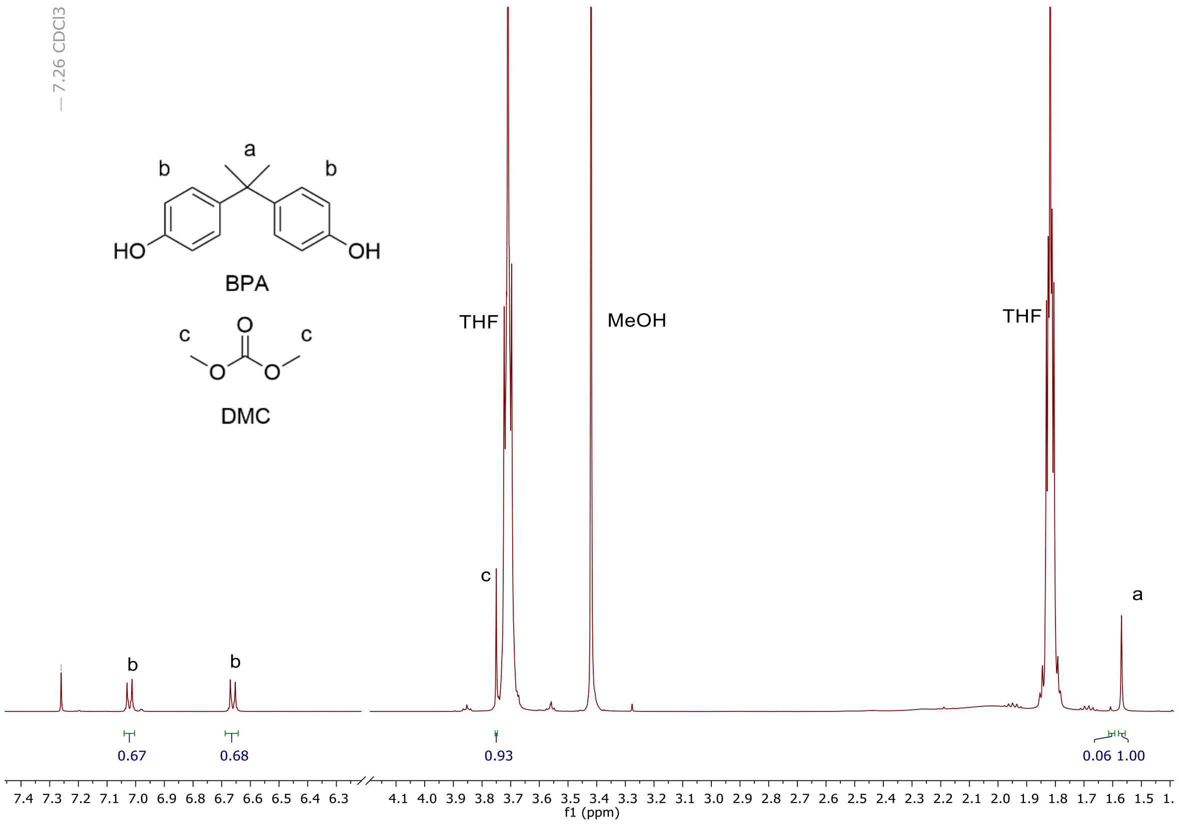


**Figure S33** $^{1}$H NMR ($CDCl_3$, 500 MHz) spectrum of BPA-PC ($M_n$ = 45,000 gmol$^{-1}$) methanolysis (17.5 equivalents MeOH) aliquot after one hour at 75 °C in THF. Selectivities calculated from literature peak examples from the methyl region (δ = 1.60 ppm).

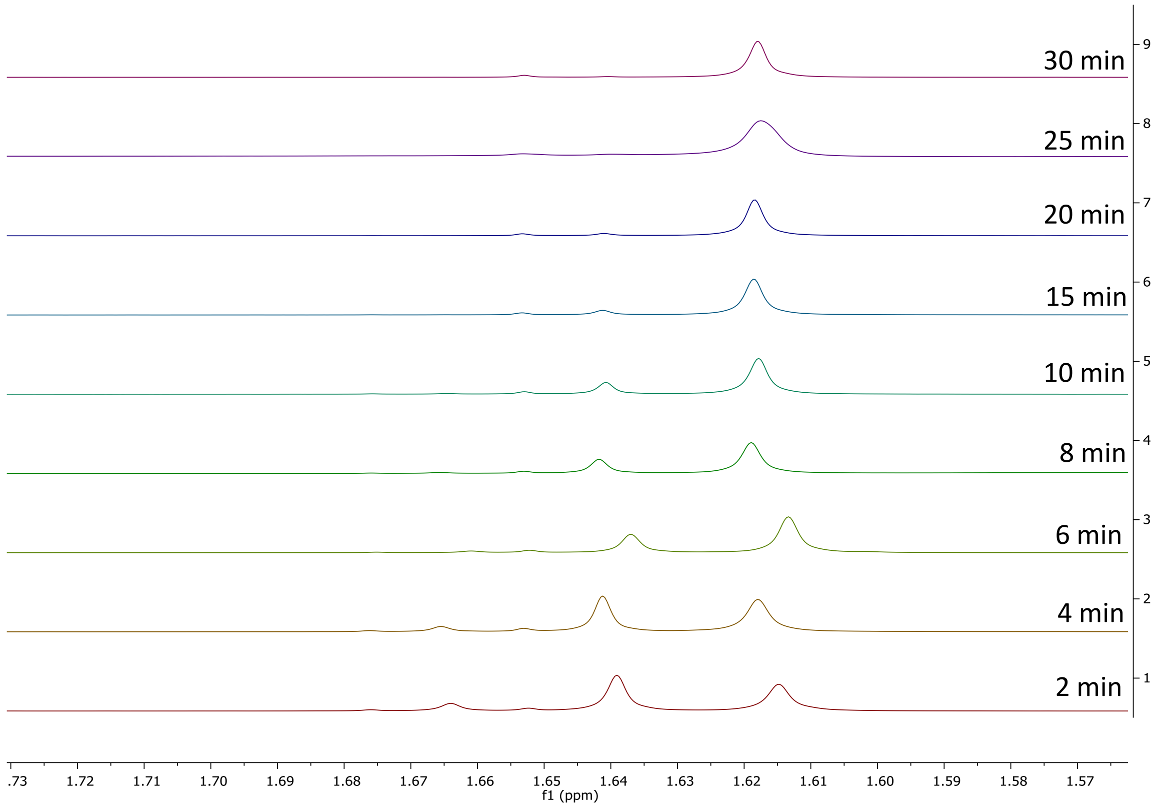


**Figure S34** Stacked $^{1}$H NMR spectra ($CDCl_3$,500 MHz) of BPA-PC methanolysis in THF at 75 °C with Zn(**3**)$_2$.

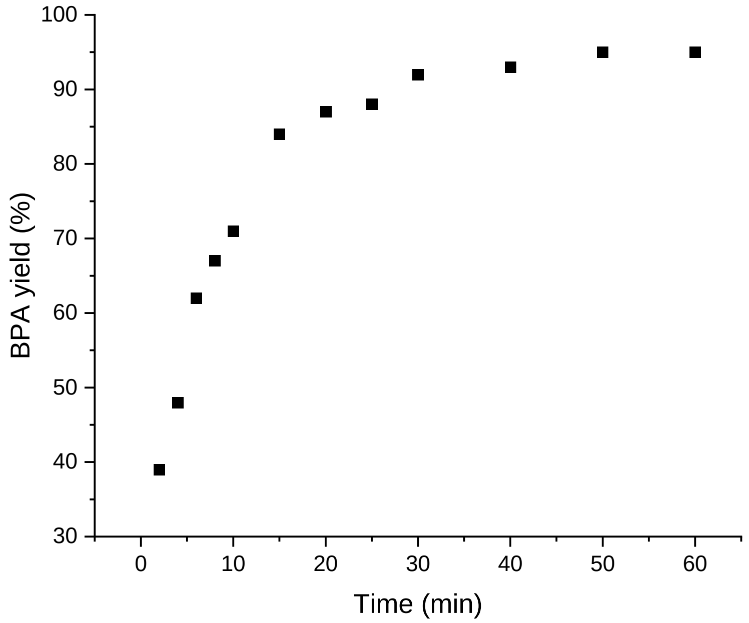


**Figure S35** Product evolution *vs.* time for BPA-PC methanolysis with Zn(**3**)$_2$ at 75 °C in THF.

# 5. Solvent-free alcoholysis of polymers

## *5.1 Solvent-free PLA methanolysis*

**Table S5** Control experiments for PLA methanolysis.[a]

| Entry | Temp. [°C] | Loading [wt%] | Time (min) | $X_{int}$ [%][b] | $S_{MeLA}$ [%][b] | $Y_{MeLA}$ [%][b] |
|---|---|---|---|---|---|---|
| 1 | 100 | 0 | 60 | 0 | 0 | 0 |
| 2 | 80 | 0 | 60 | 0 | 0 | 0 |
| 3 | 130 | 0 | 30 | 0 | 0 | 0 |
| 4 | 130 | 0 | 60 | 0 | 0 | 0 |

[a] Reaction conditions: 0.25 g of PLLA cup ($M_n$ = 45,510 g mol$^{-1}$), MeOH (2 mL, $n_{MeOH}/n_{ester}$ = 14 : 1). [b] $^1$H NMR (400 MHz, 298 K, $CDCl_3$) spectroscopy used to calculate $Y_{MeLA}$, $S_{MeLA}$ and $X_{Int}$.

**Table S6** Methanolysis of various PLA sources at 80 °C with 4 wt% Zn(**3**)$_2$.[a]

| Entry | PLA Source | Atm. | Time (min)[a] | $X_{int}$ [%][b] | $S_{MeLA}$ [%][b] | $Y_{MeLA}$ [%][b] |
|---|---|---|---|---|---|---|
| 1 | Cup | Argon | 11 | 100 | 98 | 98 |
| 2 | Fabric | Argon | 13 | 67 | 77 | 52 |
| 3 | Filament | Argon | 150 | 100 | 100 | 100 |
| 4 | Beads | Argon | 16 | 100 | 100 | 100 |
| 5 | Globe | Argon | 27 | 100 | 95 | 95 |
| 6 | Dragon | Argon | 13 | 100 | 95 | 95 |
| 7 | Filament[c] | Argon | 40 | 100 | 98 | 98 |
| 8 | Filament[d] | Argon | 13 | 100 | 98 | 98 |
| 9 | Cup[e] | Argon | 12 | 100 | 100 | 100 |

[a] Reaction conditions: 0.25 g of PLLA cup ($M_n$ = 45,510 g mol$^{-1}$), MeOH (2 mL, $n_{MeOH}/n_{ester}$ = 14 : 1). [b] $^1$H NMR (400 MHz, 298 K, $CDCl_3$) spectroscopy used to calculate $Y_{MeLA}$, $S_{MeLA}$ and $X_{Int}$. [c] Dissolved and solvent cast prior to reaction. [d] Dissolved, filtered and solvent cast prior to reaction. [e] Dissolved, filtered and solvent cast prior to reaction.

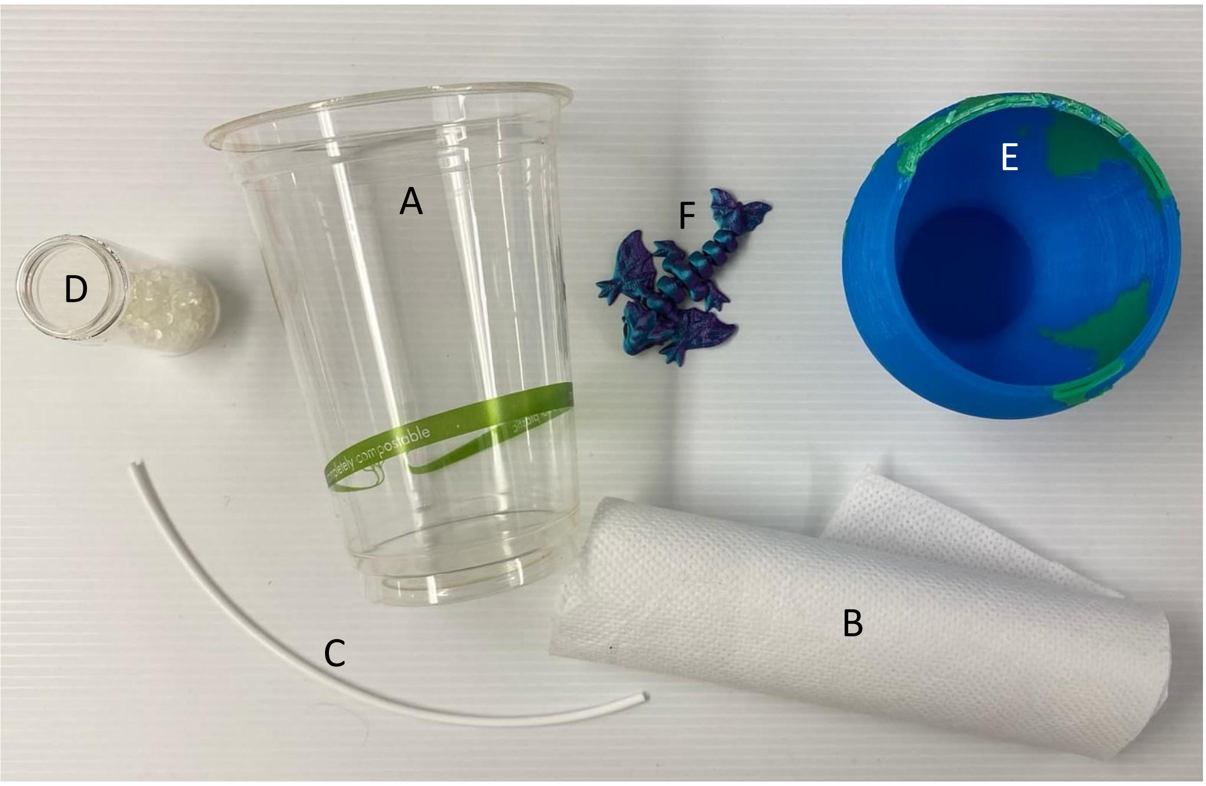


**Figure S36** PLA sources for solvent-free methanolysis. A: PLA cup, B: PLA fabric, C: PLA 3D printing filament, D: PLA 6060D beads, E: 3D printed PLA globe, F: PLA 3D printed dragon

## *5.2 Catalyst recycling*

**Table S7** Catalyst recycling experiments with Zn(**3**)$_2$ at 130 °C.[a]

| Run | Temp. [°C] | Loading [wt%] | Time (min)[b] | $X_{int}$ [%][c] | $S_{MeLA}$ [%][c] | $Y_{MeLA}$ [%][c] |
|---|---|---|---|---|---|---|
| 1 | 130 | 4 | 2 | 100 | 100 | 100 |
| 2 | 130 | 4 | 3 | 100 | 100 | 100 |
| 3 | 130 | 4 | 3 | 100 | 100 | 100 |
| 4 | 130 | 4 | 3 | 100 | 94 | 94 |
| 5 | 130 | 4 | 4 | 84 | 60 | 51 |
| 6 | 130 | 4 | 6 | 60 | 36 | 21 |
| 7 | 130 | 4 | 6 | 53 | 30 | 16 |
| 8 | 130 | 4 | 6 | 44 | 19 | 8 |
| 9 | 130 | 4 | 7 | 34 | 13 | 5 |
| 10 | 130 | 4 | 8 | 27 | 6 | 2 |
| 11 | 130 | 4 | 4 | 97 | 72 | 70 |
| 12 | 130 | 4 | 3 | 89 | 65 | 58 |
| 13 | 130 | 4 | 3 | 79 | 58 | 46 |
| 14 | 130 | 4 | 4 | 63 | 42 | 27 |
| 15 | 130 | 4 | 5 | 54 | 21 | 11 |

[a] Reaction conditions: 0.25 g of PLLA cup ($M_n$ = 45,510 g mol$^{-1}$), MeOH (2 mL, $n_{MeOH}/n_{ester}$ = 14 : 1), 4 wt% cat. loading (10 mg, 0.78 mol% relative to ester linkages). After each run, volatiles were removed and a fresh portion of PLLA cup (0.25 g) and methanol (2 mL) were added. Zn(**3**)$_2$ (10 mg) was added after run 10. [b] Time taken for disappearance of visible PLA. [c] $^1$H NMR (400 MHz, 298 K, $CDCl_3$) spectroscopy used to calculate $Y_{MeLA}$, $S_{MeLA}$ and $X_{Int}$.

**Table S8** Catalyst recycling experiments with Zn(**3**)$_2$ at 80 °C.[a]

| Run | Temp. [°C] | Loading [wt%] | Time (min)[b] | $X_{int}$ [%][c] | $S_{MeLA}$ [%][c] | $Y_{MeLA}$ [%][c] |
|---|---|---|---|---|---|---|
| 1 | 80 | 4 | 20 | 100 | 100 | 100 |
| 2 | 80 | 4 | 26 | 100 | 96 | 96 |
| 3 | 80 | 4 | 45 | 90 | 86 | 78 |
| 4 | 80 | 4 | 120 | 63 | 68 | 43 |

[a] Reaction conditions: 0.25 g of PLLA cup ($M_n$ = 45,510 g mol$^{-1}$), MeOH (2 mL, $n_{MeOH}/n_{ester}$ = 14 : 1), 4 wt% cat. loading (10 mg, 0.78 mol% relative to ester linkages). After each run, volatiles were removed and a fresh portion of PLLA cup (0.25 g) and methanol (2 mL) were added. [b] Time taken for disappearance of visible PLA. [c] $^1$H NMR (400 MHz, 298 K, $CDCl_3$) spectroscopy used to calculate $Y_{MeLA}$, $S_{MeLA}$ and $X_{Int}$.

## *5.3 Solvent and Product Isolation*

**Table S9** Solvent and MeLA isolation experiments with Zn(**3**)$_2$ at 80 °C.

| Entry | Temp. [°C] | Scale [g] | Loading [wt%] | Time (min)[c] | $X_{int}$ [%][d] | $S_{MeLA}$ [%][d] | $Y_{MeLA}$ [%][d] |
|---|---|---|---|---|---|---|---|
| 1[a] | 80 | 25 | 4 | 30 | 100 | 99 | 99 |
| 2[b] | 80 | 0.25 | 4 | 10 | 100 | 99 | 99 |

[a] Reaction conditions: 25 g of PLLA cup ($M_n$ = 45,510 g mol$^{-1}$), MeOH (200 mL, $n_{MeOH}/n_{ester}$ = 14 : 1), 4 wt% cat. loading (1 g, 0.78 mol% relative to ester linkages). [b] Reaction conditions: 0.25 g of PLLA cup ($M_n$ = 45,510 g mol$^{-1}$), reclaimed MeOH (2 mL, $n_{MeOH}/n_{ester}$ = 14 : 1), 4 wt% cat. loading (10 mg, 0.78 mol% relative to ester linkages). [c] Time taken for disappearance of visible PLA. [d] $^1$H NMR (400 MHz, 298 K, $CDCl_3$) spectroscopy used to calculate $Y_{MeLA}$, $S_{MeLA}$ and $X_{Int}$.

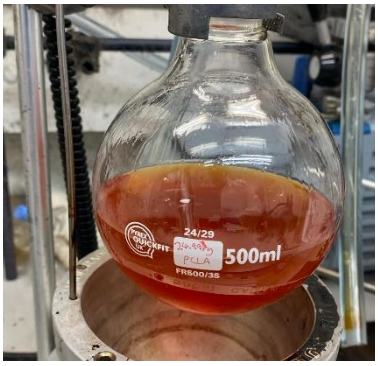


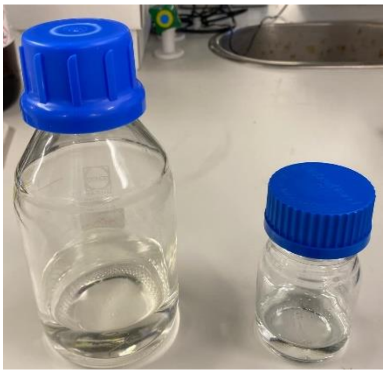

**Figure S37** Left: Final reaction mixture from 25 g scale, solvent-free PLA methanolysis (**Table S9, entry 1**). Right: Isolated methanol and methyl lactate. $[\alpha]_D$ = -8.0°. Consistent with literature value for pure *L*-methyl lactate ($[\alpha]_D$ = -8.3°).[4]

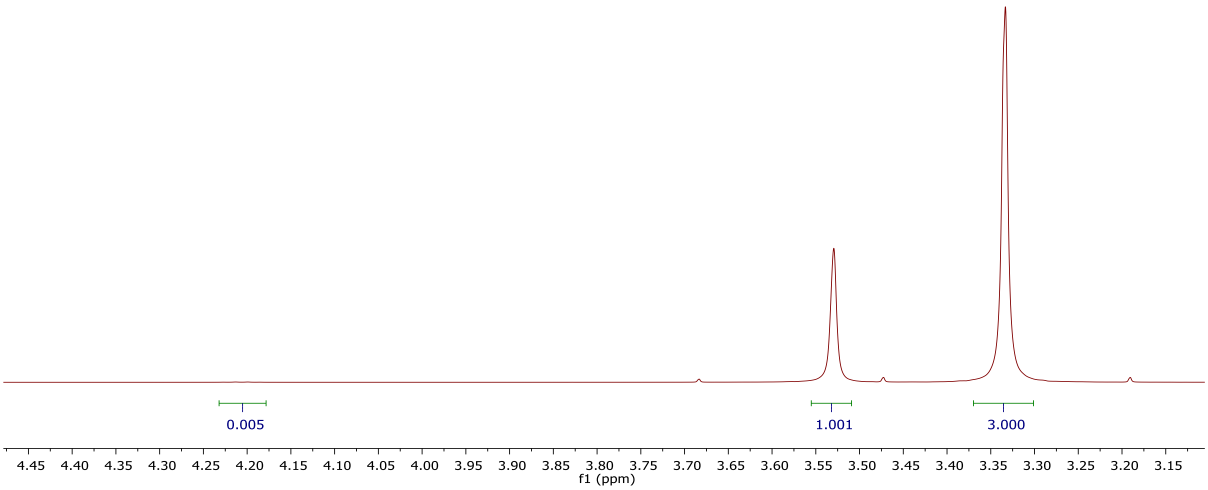


**Figure S38** $^{1}$H NMR spectrum ($CDCl_3$, 400 MHz) of reclaimed MeOH with trace MeLA.

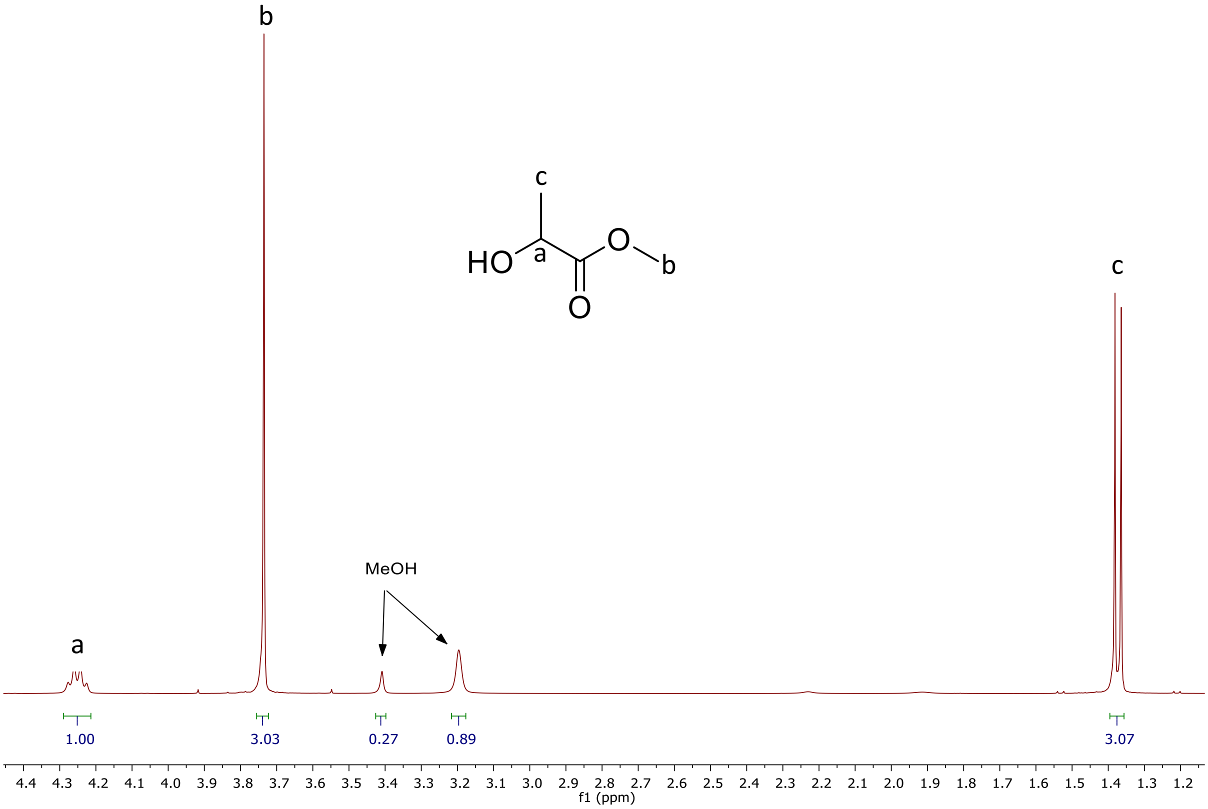


**Figure S39** $^{1}$H NMR spectrum ($CDCl_3$, 400 MHz) of isolated MeLA in a 3 : 1 molar ratio with MeOH.

## 5.3 PET glycolysis

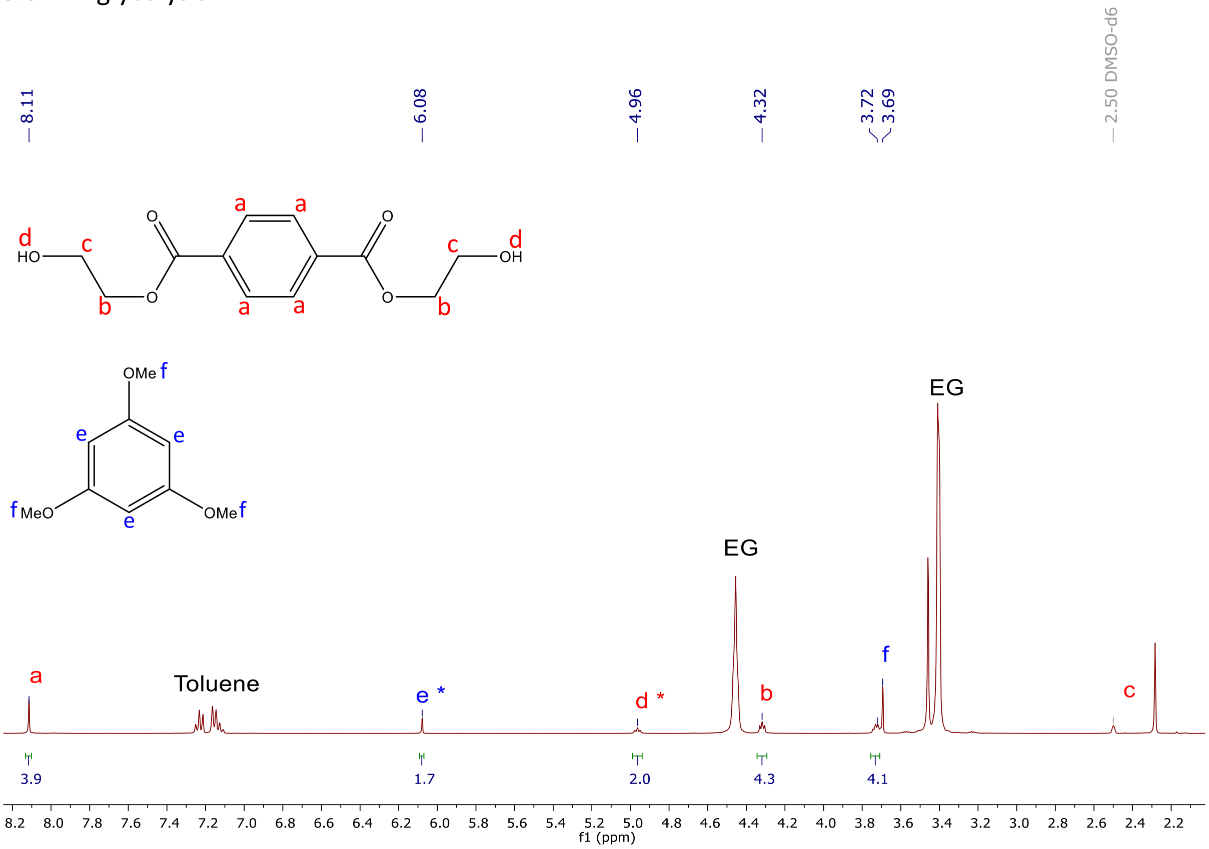


**Figure S40** $^{1}H$ NMR (400 MHz, DMSO-d6) spectrum for PET degradation (**Table 6**, **entry 1**) with internal standard (trimethoxybenzene in toluene) added. Starred peaks were used for determination of BHET yield.

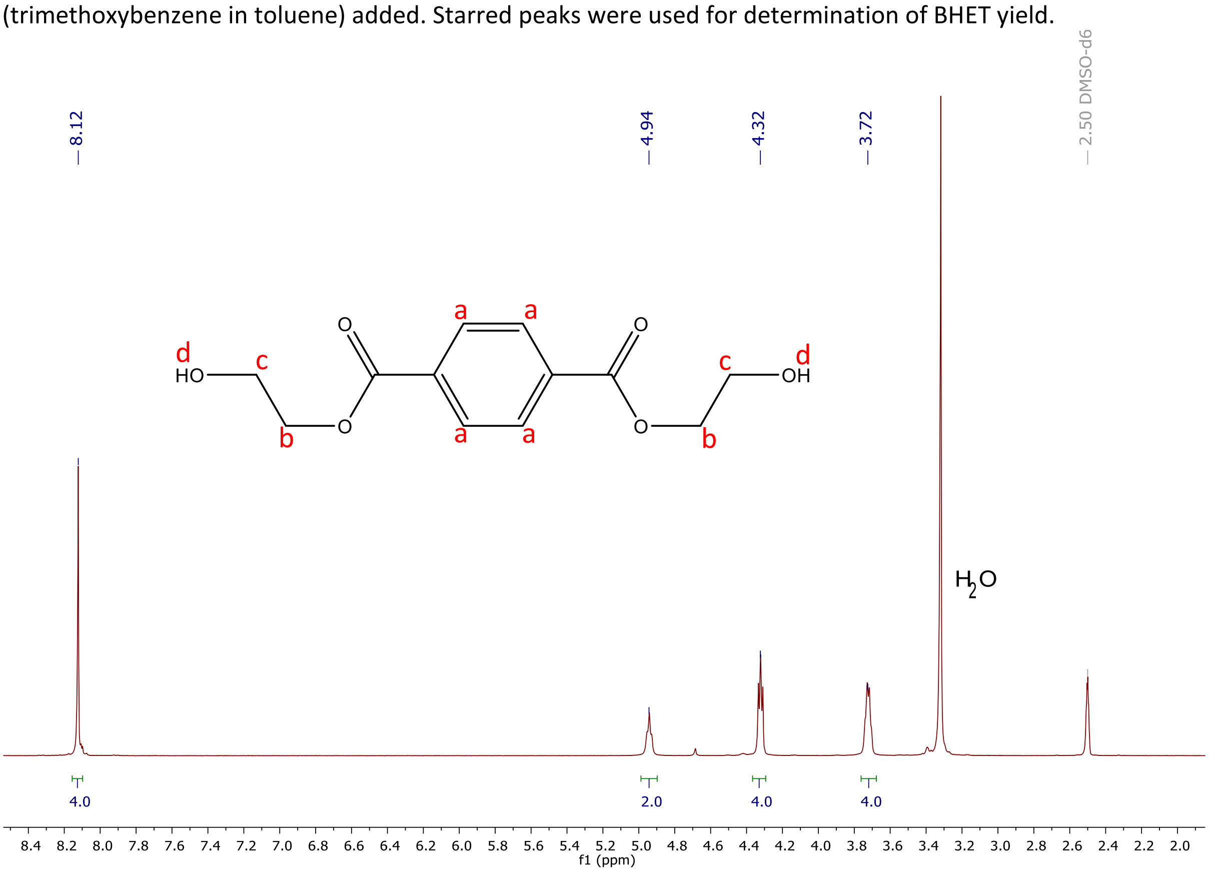


**Figure S41** $^{1}H$ NMR (400 MHz, DMSO-d6) spectrum for recrystallised BHET.

# 6 Mixed polymer degradation

## *6.1 Solution methanolysis of PLA/BPA-PC*

**Table S10** Investigation of selectivity PLA/BPA-PC methanolysis after 30 minutes.

| Entry | Cat. | Temp. [°C] | Loading [wt%] | ROH [eq.] | Alcohol | $X_{PLA}$ [%] | $Y_{MeLA}$ [%] | $X_{BPA\text{-}PC}$ [%] | $Y_{BPA}$ [%] |
|---|---|---|---|---|---|---|---|---|---|
| 1 | Zn(**1**)$_2$ | 50 | 4 | 9.5 | MeOH | 54 | 13 | 98 | 23 |
| 2 | Zn(**2**)$_2$ | 50 | 4 | 9.5 | MeOH | 60 | 14 | 100 | 34 |
| 3 | Zn(**3**)$_2$ | 50 | 4 | 9.5 | MeOH | 78 | 31 | 100 | 40 |
| 4 | Zn(**2**)$_2$ | 30 | 4 | 9.5 | MeOH | 41 | 5 | 99 | 13 |
| 5 | Zn(**2**)$_2$ | 80 | 4 | 9.5 | MeOH | 100 | 83 | 100 | 72 |
| 6 | Zn(**2**)$_2$ | 50 | 2 | 9.5 | MeOH | 58 | 12 | 100 | 30 |
| 7 | Zn(**2**)$_2$ | 50 | 8 | 9.5 | MeOH | 99 | 46 | 100 | 80 |
| 8 | Zn(**2**)$_2$ | 50 | 4 | 5 | MeOH | 51 | 10 | 100 | 27 |
| 9 | Zn(**2**)$_2$ | 50 | 4 | 15 | MeOH | 86 | 34 | 100 | 47 |
| 10 | Zn(**2**)$_2$ | 50 | 4 | 9.5 | EtOH | 60 | 33 | 100 | 8 |
| 11 | Zn(**2**)$_2$ | 50 | 4 | 9.5 | nBuOH | 52 | 30 | 97 | 7 |
| 12 | Zn(**2**)$_2$ | 30 | 4 | 5 | MeOH | 20 | 1 | 94 | 15 |

## *6.2 Example mixed polymer $^{1}$H NMR sepctra*

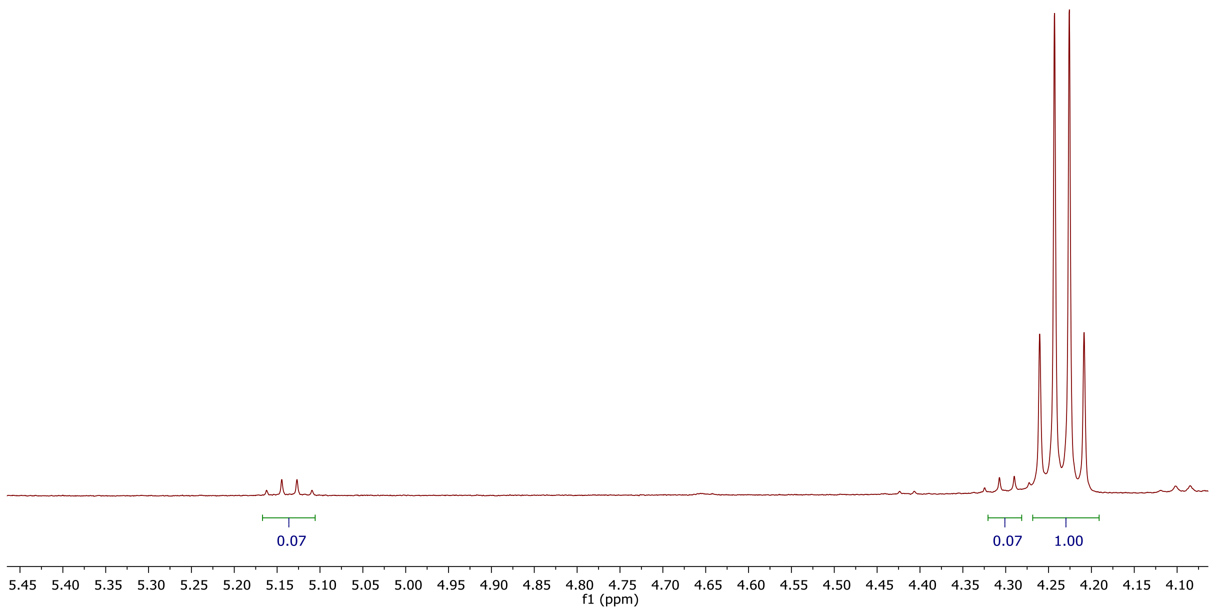


**Figure S42** PLA methanolysis in the presence of PET (**Table 7, entry 1**).

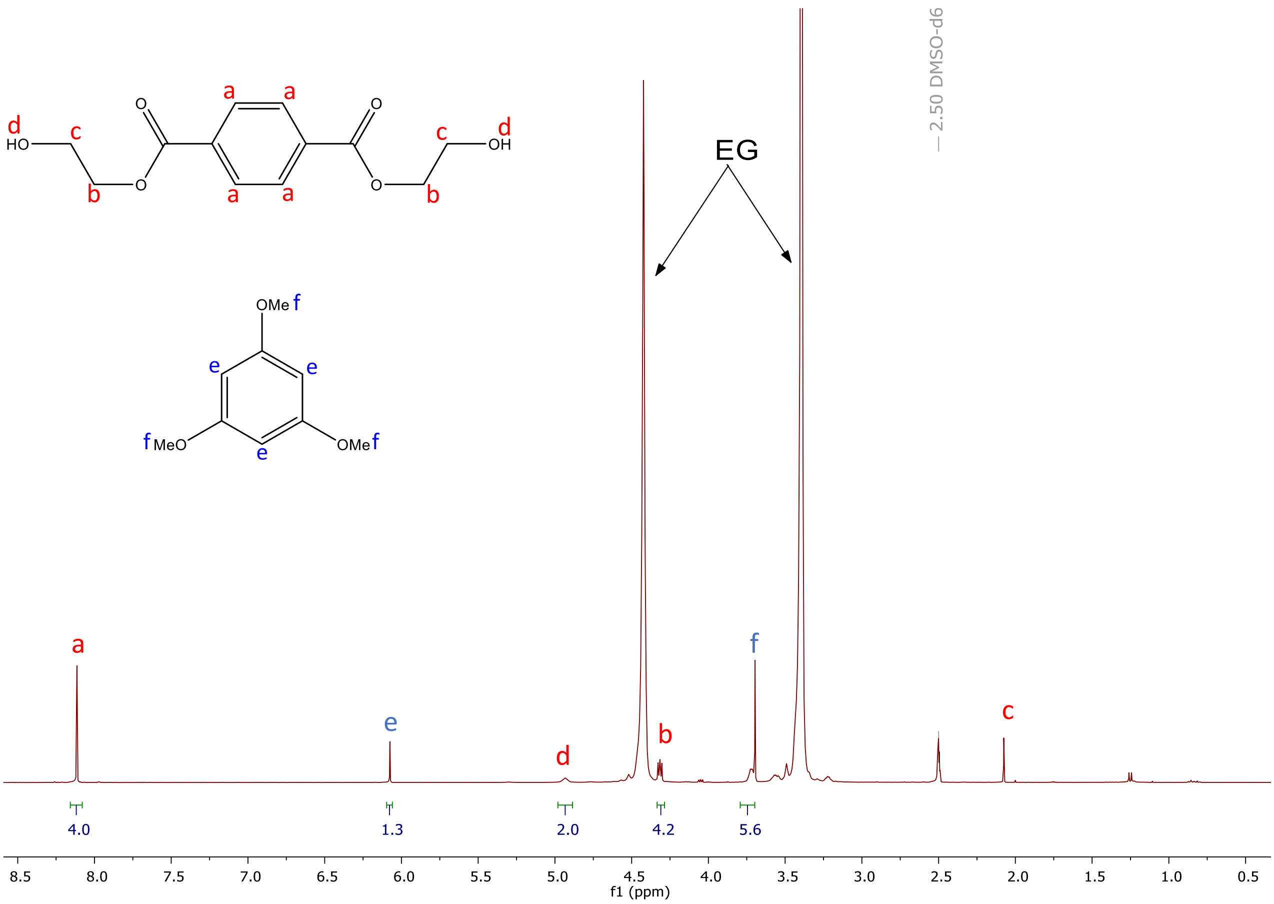


**Figure S43** PET glycolysis after PLA methanolysis (**Table 7, entry 1**).

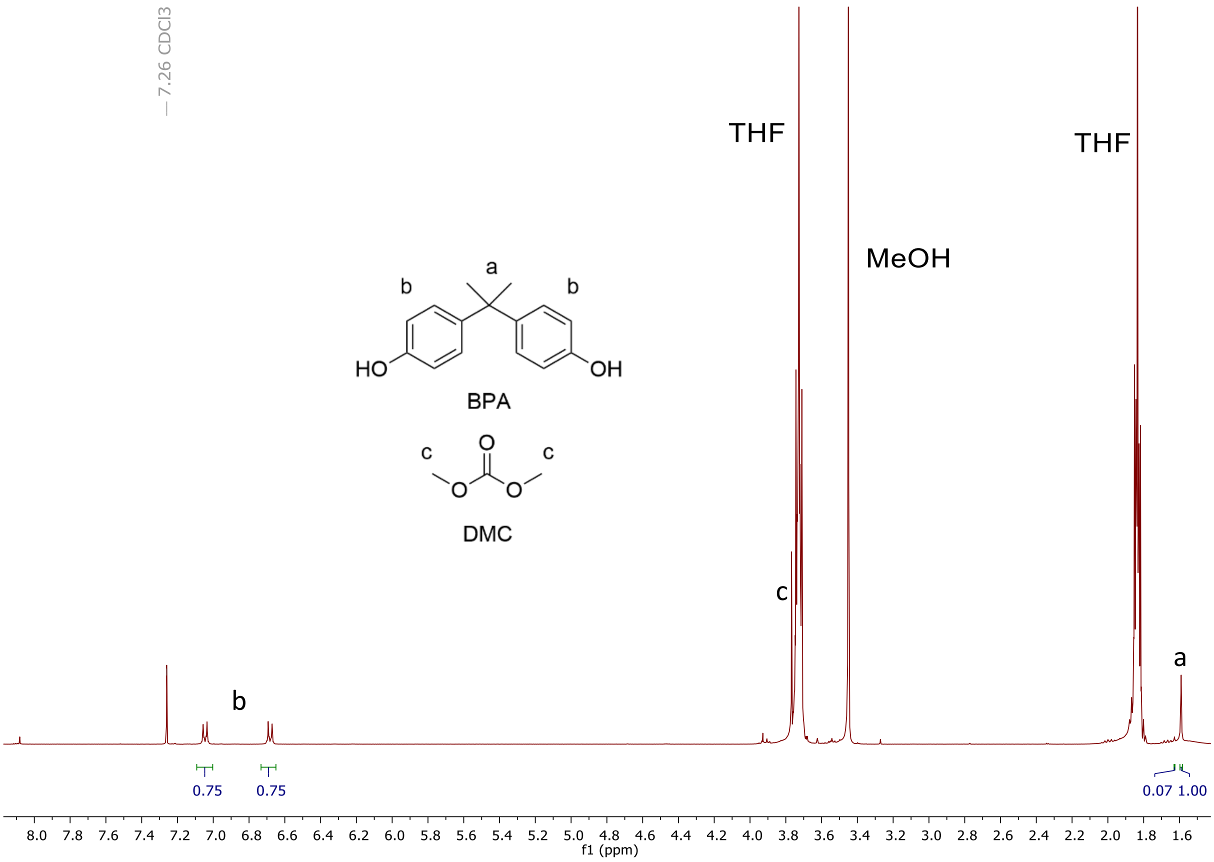


**Figure S44** BPA-PC methanolysis in the presence of PET (**Table 7, entry 3**).

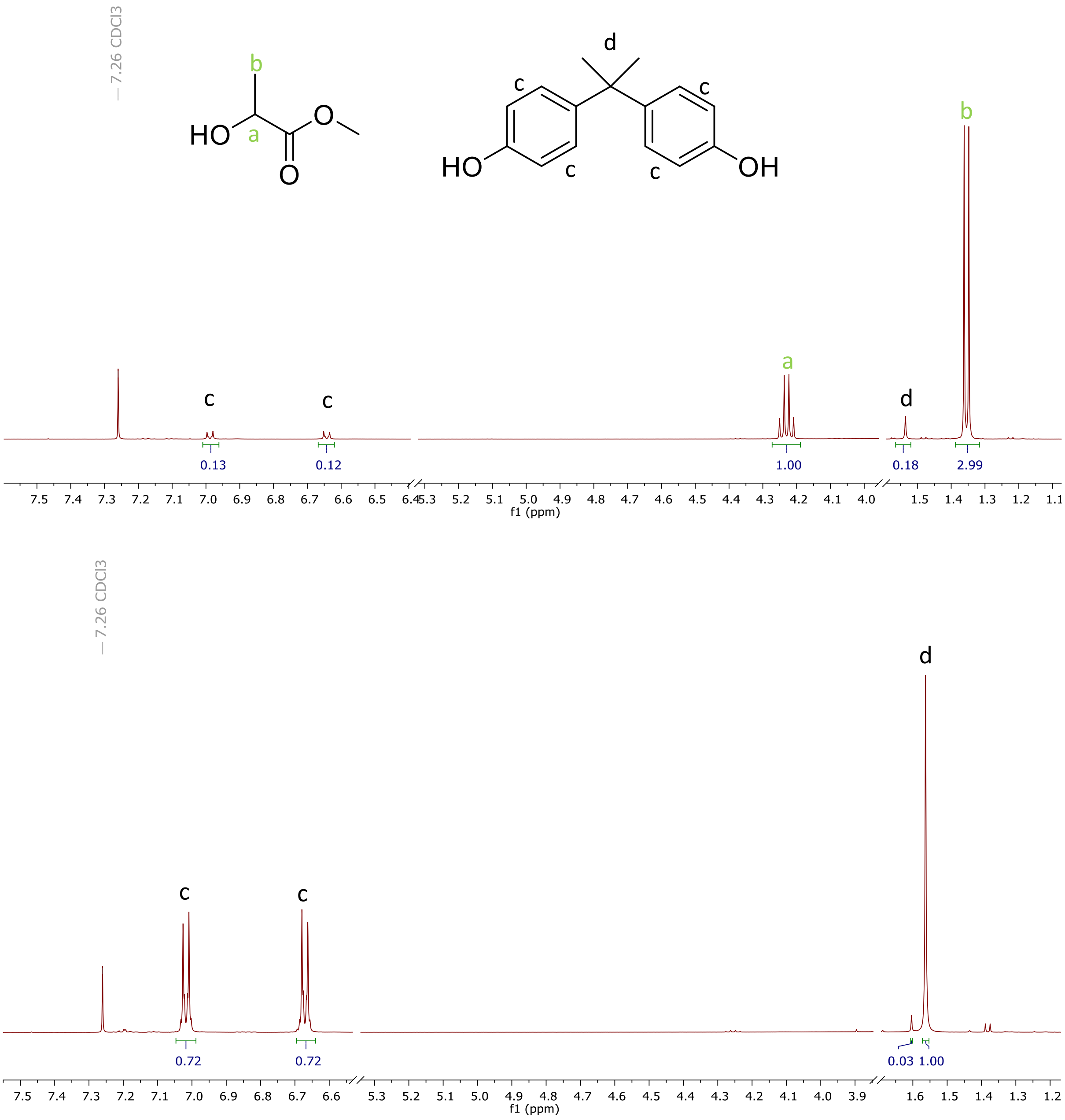


**Figure S45** $^{1}$H NMR spectra (400 MHz, CDCl3) of sequential PLA/BPA-PC degradation after PLA methanolysis (top) and BPA-PC methanolysis (bottom). Some dissolved BPA/BPA-PC can be observed in top spectrum.

## 7. Crystallography data

**Table S11** X-ray crystallographic parameters.[5]

| Compound reference | Zn(**1**)$_2$ | Zn(**2**)$_2$ | Zn(**3**)$_2$($CO_2$) |
|---|---|---|---|
| Chemical formula | $C_{18}H_{28}N_6Zn$ | $C_{21}H_{36}N_6OZn$ | $C_{17}H_{24}N_6O_2Zn$ |
| Formula Mass | 393.83 | 453.93 | 409.79 |
| Crystal system | Monoclinic | Monoclinic | Orthorhombic |
| *a*/Å | 8.85830(10) | 19.5252(2) | 14.7186(3) |
| *b*/Å | 19.0839(3) | 12.0366(2) | 13.7112(3) |
| *c*/Å | 11.7106(2) | 9.75260(10 | 17.6395(3) |
| *α*/° | 90 | 90 | 90 |
| *β*/° | 92.1640(10) | 93.0530(10) | 90 |
| *γ*/° | 90 | 90 | 90 |
| Unit cell volume/Å$^3$ | 1978.28(5) | 2288.77(5) | 3559.82(12) |
| Temperature/K | 150(2) | 150(2) | 150(2) |
| Space group | P $2_{1/n}$ | P $2_{1/c}$ | P b c a |
| No. of formula units per unit cell, *Z* | 4 | 4 | 8 |
| Radiation type | Cu Kα | Cu Kα | Cu Kα |
| Absorption coefficient, $\mu$/mm$^{-1}$ | 1.813 | 1.668 | 1.405 |
| No. of reflections measured | 37585 | 45278 | 37119 |
| No. of independent reflections | 3938 | 4575 | 4980 |
| $R_{int}$ | 0.0467 | 0.0448 | 0.0354 |
| Final $R_1$ values ($I > 2\sigma(I)$) | 0.0412 | 0.0513 | 0.0287 |
| Final $wR(F^2)$ values ($I > 2\sigma(I)$) | 0.1085 | 0.1375 | 0.0618 |
| Final $R_1$ values (all data) | 0.0436 | 0.0543 | 0.0385 |
| Final $wR(F^2)$ values (all data) | 0.1108 | 0.1379 | 0.0654 |
| Goodness of fit on $F^2$ | 1.052 | 1.136 | 1.045 |
| CCDC deposition number | 2421210 | 2421211 | 2421212 |

## 8. References


[1] J. Stewart, M. Fuchs, J. Payne, O. Driscoll, G. Kociok-Köhn, B. D. Ward, S. Herres-Pawlis, M. D. Jones, *RSC Adv.* **2022**, *12*, 1416–1424.

[2] J. M. Payne, M. Kamran, M. G. Davidson, M. D. Jones, *ChemSusChem* **2022**, *15*, e202200255.

[3] J. Castro, A. Castineiras, M. L. Duran, J. A. Garcia-Vazquez, A. Macias, J. Romero, A. Sousa, *Zeitschrift für Anorg. und Allg. Chemie* **1990**, *586*, 203–208.

[4] Lide, D.R. (ed). CRC Handbook of Chemistry and Physics. 74th ed. CRC Press, 1993-1994, p. 3-304.

[5] Deposition numbers 2421210 (for Zn(**1**)$_2$), 2421211 (for Zn(**2**)$_2$), and 2421212 (for Zn(**3**)$_2$($CO_2$)) contain the supplementary crystallographic data for this paper. These data are provided free of charge by the joint Cambridge Crystallographic Data Centre and Fachinformationszentrum Karlsruhe Access Structures service.
